# 1,2,4-Oxadiazole/2-Imidazoline Hybrids: Multi-target-directed Compounds for the Treatment of Infectious Diseases and Cancer

**DOI:** 10.3390/ijms20071699

**Published:** 2019-04-05

**Authors:** Anton Shetnev, Sergey Baykov, Stanislav Kalinin, Alexandra Belova, Vladimir Sharoyko, Anton Rozhkov, Lev Zelenkov, Marina Tarasenko, Evgeny Sadykov, Mikhail Korsakov, Mikhail Krasavin

**Affiliations:** 1 Pharmaceutical Technology Transfer Center, Ushinsky Yaroslavl State Pedagogical University, 108 Respublikanskaya St., Yaroslavl 150000, Russia; a.shetnev@yspu.org (A.S.); a.belova@yspu.org (A.B.); m.korsakov@yspu.org (M.K.); 2 Institute of Chemistry, Saint Petersburg State University, 26 Universitetskii Pr, Peterhof, Saint Petersburg 198504, Russia; stanislaw.k@mail.ru (S.K.); v.sharoyko@spbu.ru (V.S.); a.rozhkov@spbu.ru (A.R.); chemleo@protonmail.ch (L.Z.); m.krasavin@spbu.ru (M.K.); 3 Yaroslavl State Technical University, 88 Moscowsky Pr, Yaroslavl 150023, Russia; mkarunnaya@mail.ru; 4 A.E. Favorsky Irkutsk Institute of Chemistry, Siberian Branch Russian Academy of Science, 1 Favorsky Str, Irkutsk 664033, Russian; esadyk.chem@gmail.com

**Keywords:** 1,2,4-oxadiazole, 2-imidazolines, antibacterial, cytotoxicity, MAO inhibition

## Abstract

Replacement of amide moiety with the 1,2,4-oxadiazole core in the scaffold of recently reported efflux pump inhibitors afforded a novel series of oxadiazole/2-imidazoline hybrids. The latter compounds exhibited promising antibacterial activity on both Gram-positive (*Staphylococcus aureus*, *Bacillus subtilis*) and Gram-negative (*Escherichia coli*, *Pseudomonas* *fluorescens*) strains. Furthermore, selected compounds markedly inhibited the growth of certain drug-resistant bacteria. Additionally, the study revealed the antiproliferative activity of several antibacterial frontrunners against pancreas ductal adenocarcinoma (PANC-1) cell line, as well as their type-selective monoamine oxidase (MAO) inhibitory profile.

## 1. Introduction

The drug resistance of microbial pathogens is one of the greatest challenges to human health these days [1]. It is, therefore, of utmost importance to discover novel antibacterial scaffolds, as well as improve our knowledge of how we can employ already studied compounds and their analogs for the treatment of bacterial diseases.

There are few known mechanisms of bacterial drug resistance, each of which makes a pathogen invincible for commonly used agents [2]. Particularly, in some cases, deactivation of the antibiotic occurs through enzymatic degradation or modification of the antibiotic molecule rendering it inactive. Some bacteria employ protection, alteration or overexpression of the antibiotic target. Meanwhile, decreasing the expression of the porin or expressing more selective porin variant helps to prevent antibiotics from penetrating the cell membrane or cell wall. Finally, some pathogens efficiently use efflux systems, maintaining the intracellular concentration of the antibiotic lower than the minimum inhibitory levels.

**Scheme 1:**
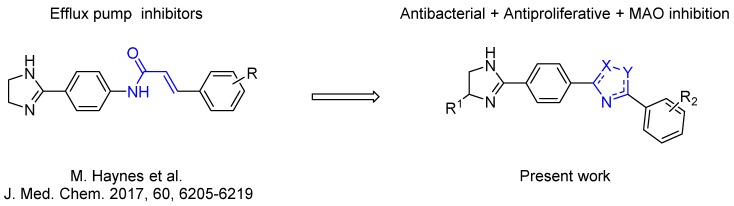
The design of a new series of antibacterial agents [5].

Considering this, intensive efforts have been made to find the possibility to specifically block the efflux function in prokaryotic cells. The latter effect, if achieved, causes the loss of the ability of bacteria to counter the effects of antibiotics, and thus restores the pathogens drug sensitivity [3,4]. A recent study based on this idea is the one reported by Heynes et al. who discovered a new class of efflux pump inhibitors in prokaryotic cells based on 2-imidazolines derivatives (Scheme 1) [5]. Although these compounds alone did not show pronounced antibacterial properties, they markedly potentiated the effect of Novobiocin on several *E. coli* strains, inhibiting the efflux pump expressed by these bacteria. Despite the intriguing activity exhibited by these compounds, the presence of amide moiety as the central “core” of the molecule can be considered a disadvantage of the newly discovered chemotype of inhibitors as they are expected to be rapidly hydrolyzed in vivo [6]. Thus, we came up with an idea to examine 1,2,4-oxadiazole ring as an alternative core for the promising 2-imidazoline-containing periphery, as it could provide novel two-heterocycles-hybrid based series of compounds for further diverse biological evaluation. These expectations were supported by several successful series of variously biologically active oxadiazoles synthesized and evaluated in our group [7,8,9,10], as well as by recent example of anti-MRSA (Methicillin-Resistant *Staphylococcus aureus*) and anti-VRE (Vancomycin-Resistant *Enterococcus*) active antibiotics incorporating 3-phenoxyphenyl-substituted 1,2,4-oxadiazoles, that have been recently reported [11,12,13,14,15,16,17].

## 2. Result and Discussion

### 2.1. Chemistry

**Scheme 2:**
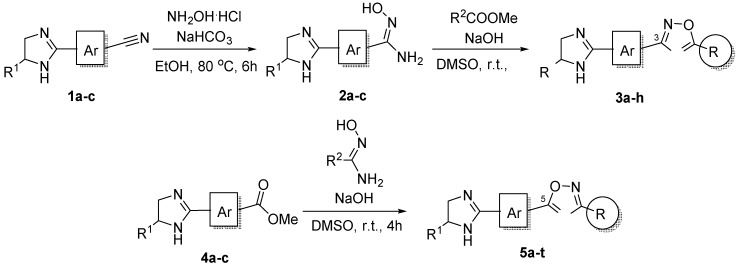
Synthesis of 1,2,4-oxadiazoles **3a-h** and **5a-t**.

The original approach to the synthesis of 1,2,4-oxadiazoles **3a-g** and **5a-q** has been previously described by our group (for more information see Supplementary Material) [9,18,19,20,21], and the four newly prepared compounds **3h** and **5r-t** were obtained in the similar manner (Scheme 2).

### 2.2. Antibacterial Activity

A screening of the antibacterial activity was carried out on compounds **3a-h** and **5a-t** against a number of both Gram-positive and Gram-negative nonpathogenic bacteria (*Staphylococcus aureus*, *Escherichia coli*, *Bacillus subtilis*, *Pseudomonas fluorescens*), as well as three pathogenic strains, including *E. coli* and *Enterobacter* spp.

Antimicrobial activities were determined by the microbroth dilution method using the Clinical Laboratory Standards Institute (CLSI) recommendations and are summarized in Table 1 (See Experimental) [22].

Evaluated compounds exhibited medium, weak or no antibacterial activity. The most potent antimicrobial agents turned out to have some degree of structural similarity, representing two motifs: halogenated aromatic ring (R^2^) and methyl substituted imidazoline (R^1^ = Me). The values of minimal inhibition concentrations (MICs) for the listed compounds were in the region between 8 and 32 µg/mL.

Obviously, the introduction of alkyl into the R^2^ position (**3a**, **3c**, and **5b**) dramatically decreased the antibacterial activity of compounds, resulting in the MIC values higher than 256 µg/mL; while the activity dropped even more upon substitution of alkyl group for the large aromatic moieties (**3g**, **5a**, **5m**, **5c**). Furthermore, no activity was observed for the rest of heteroaryl-substituted derivatives, that is, for those incorporating triazole, imidazole, and thiophene (**3f**, **5f**, **5l**, **5o**, **5q**) rings. Finally, the derivatives bearing alkyl substituted phenyl ring in R^2^ position (**3** and **5**) displayed no or poor activity (≥32 μg/mL) (**3b**, **3h**, **5d**, **5e**, **5j**, **5k**, **5n**, **5t**) with the exception of compound **5r**.

Most potent antimicrobial agents **3d**, **3e**, **5g**, **5k**, **5r**, and **5s** identified in the screening were additionally tested against ampicillin and rifampicin resistant strains of *E. coli*, as well as against clinically isolated poly-resistant strain of *Enterobacter* spp. The observed MIC values (µg/mL) are represented in Table 2.

Except for **5k**, studied compounds showed activity against all tested strains, and **3d** displayed MIC values in the range of 8–16 µg/mL, thus being a promising candidate for further development as an antibacterial agent.

### 2.3. MAO Inhibition Studies

It is well known that the use of antibiotics is often accompanied by some undesired effects. In particular, some antibiotics, such as linezolid [23] and furazolidone [24], are able to block the function of, monoamine oxidase (MAO) enzyme, increasing the risk of certain adverse effects, such as the “cheese reaction”, serotonin syndrome [25], etc. Among others, some 2-imidazolines are also known to inhibit MAOs [26]. Recently, we investigated the properties of MAO inhibition for the compounds **3a-g** and **5a-q** (Table 3) [9]. Selected results are listed in Table 3, demonstrating that some of the prepared 2-imidazolines are high potency inhibitors of human MAO-B, while comparatively lower potencies have been recorded for human MAO-A inhibition.

These compounds are thus specific inhibitors of MAO-B. Since adverse reactions, such as the “cheese reaction” or serotonin syndrome, are associated with the inhibition of MAO-A, the lower potency inhibition of MAO-A displayed by these compounds greatly reduces the risk for these undesired effects. We conclude that MAO inhibition exhibited by these compounds should not be a limitation for further development as antimicrobial agents.

### 2.4. Cytotoxicity Assay

**Figure 1:**
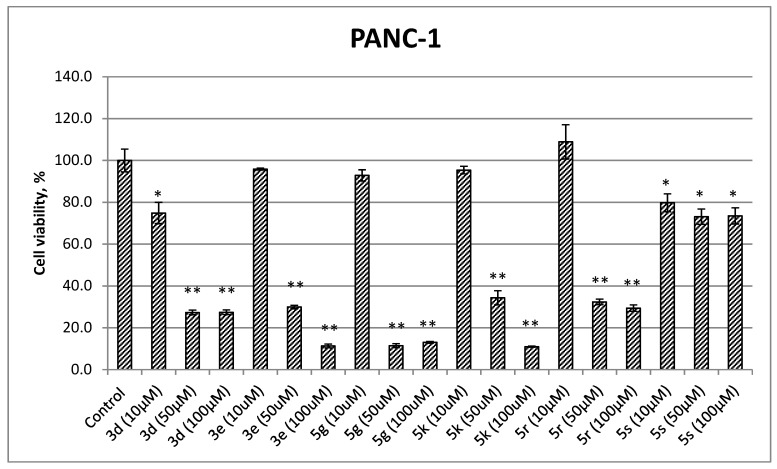
Cell viability MTT assay results for compounds **3d**, **3e**, **5g**, **5k**, **5r**, and **5s** at 10, 50, 100 µM concentrations against pancreas ductal adenocarcinoma (PANC-1) cell line (values are shown as the mean ± SEM of three experiments: (*) *p* < 0.05 and (**) *p* < 0.01 in comparison to control (0 µM)).

The ability of several antibacterial frontrunner compounds (with MIC ≤ 64 µg/mL) **3d**, **3e**, **5g**, **5k**, **5r**, and **5s** to affect the viability of pancreas ductal adenocarcinoma (PANC-1) cell line at 10, 50, and 100 µM concentrations was evaluated using the MTT assay (Figure 1) [27,28]. Interestingly, the experiment revealed no or mild cytotoxic effect of all the compounds shown at 10 µM concentration. However, at higher concentrations (50 and 100 µM), most compounds displayed pronounced effect on the survival of cancer cell, decreasing cell viability to the level of less than 35% in comparison to 0 µM inhibitor control. Meanwhile, increasing concentration of compound **5s** did not lead to substantial changes in the survival of PANC-1 culture cells. Thus, some of the prepared compounds exhibit antiproliferative and cytotoxic activity at a low micromolar range of concentrations. In combination with the antimicrobial activity displayed at the same level of concentrations, this data may gain the attention of medicinal chemists as the anticancer potential of some antibiotics has been thoroughly studied during the last years [29,30].

## 3. Materials and Methods

### 3.1. General Methods

All reagents and solvents were obtained from commercial sources and were used without purification. DMSO was dried over molecular sieves (4 Å). Reactions were monitored by analytical thin layer chromatography (Merck KGaA, Darmstadt, Germany) using Macherey-Nagel TLC sheets (Silufol UV-254), and the developed sheets were visualized under UV light. NMR spectra were recorded on Bruker AVANCE DPX 400 (RUKER Corporation, Billerica, MA, USA) at 400 MHz and 101 MHz for ^1^H and ^13^C, respectively. Chemical shifts are reported as parts per million (δ, ppm) and were referenced to the residual solvent peaks at 7.26 and 2.50 ppm for ^1^H in CDCl_3_ and DMSO-*d*_6_, respectively, and 77.16 and 39.52 ppm for ^13^C in CDCl_3_ and DMSO-*d*_6_, respectively. Multiplicities are abbreviated as follows: s, singlet; d, doublet; t, triplet; q, quartet; m, multiplet; br, broad. Coupling constant, J, is reported in Hertz (Hz). Melting points were determined in open capillary tubes with an Electrothermal IA 9300 series Digital Melting Point Apparatus (Electrothermal, Standfoshire, UK). High-resolution mass spectra (HRMS) were recorded with a Bruker maXis HRMS-ESI-QTOF (RUKER Corporation, Billerica, MA, USA) equipped with electrospray ionization source and MeOH was used as the solvents.

### 3.2. General Procedure for the Synthesis of 3,5-Disubstituted-1,2,4-oxadiazoles (**3a-h** and **5a-t**)

To a solution of the amidoxime (2 mmol) and the appropriate carboxylic ester (2 mmol) in DMSO (1 mL), 120 mg (3 mmol) powdered NaOH was rapidly added. The reaction mixture was stirred at room temperature for the required time (TLC or precipitation of the product). The reaction mixture was diluted with cold water (30–50 mL), and the resulting precipitate was collected by filtration, washed with water (30 mL), dried and purified by column chromatography (eluent: 5% methanol/95% chloroform).

**3-(4-(4,5-Dihydro-1H-imidazol-2-yl)phenyl)-5-(4-ethylphenyl)-1,2,4-oxadiazole (3h)**

White powder, 34% (216 mg) yield, m.p. 196–198 °C. ^1^H NMR (400 MHz, DMSO) δ 8.14 (m, 4H), 8.04 (d, *J* = 8.4 Hz, 2H), 7.53 (d, *J* = 8.2 Hz, 2H), 7.07 (br s, 1H), 3.66 (s, 4H), 2.75 (q, *J* = 7.6 Hz, 2H), 1.25 (t, *J* = 7.6 Hz, 3H). ^13^C NMR (101 MHz, DMSO) δ 176.1, 168.3, 163.4, 150.4, 133.9, 129.5, 128.5, 128.4, 128.1, 127.4, 121.3, 28.7, 15.5. HRMS (ESI) calcd for C_19_H_18_N_4_O [M+H]^+^ 319.1553, found 319.1573.

**5-(4-(5-Methyl-4,5-dihydro-1H-imidazol-2-yl)phenyl)-3-(p-tolyl)-1,2,4-oxadiazole (5r)**

Pale powder, 59% (330 mg) yield, m.p. 89–90 °C. ^1^H NMR (400 MHz, DMSO) δ 8.23 (d, *J* = 8.4 Hz, 2H), 8.08 (d, *J* = 8.4 Hz, 2H), 7.99 (d, *J* = 8.1 Hz, 2H), 7.41 (d, *J* = 8.0 Hz, 2H), 7.22 (br s, 1H), 4.09–3.98 (m, 1H), 3.81 (t, *J* = 11.1 Hz, 1H), 3.24 (dd, *J* = 11.8, 7.9 Hz, 1H), 1.20 (d, *J* = 6.3 Hz, 3H). ^13^C NMR (101 MHz, DMSO) δ 175.3, 168.8, 161.7, 142.1, 135.3, 130.3, 128.5, 128.3, 127.5, 125.0, 123.8, 22.4, 21.6. HRMS (ESI) calcd for C_19_H_18_N_4_O [M+H]^+^ 319.1553, found 319.1555.

**3-(3-Chlorophenyl)-5-(4-(5-methyl-4,5-dihydro-1H-imidazol-2-yl)phenyl)-1,2,4-oxadiazole (5s)**

White powder, 36% (243 mg) yield, m.p. 139–141 °C. ^1^H NMR (400 MHz, DMSO) δ 8.16 (m, 4H), 8.03 (d, *J* = 8.2 Hz, 2H), 7.83 (d, *J* = 8.1 Hz, 1H), 7.71 (t, *J* = 7.9 Hz, 1H), 7.18 (br s, 1H), 4.09–3.96 (m, 1H), 3.81 (t, *J* = 11.0 Hz, 1H), 3.23 (dd, *J* = 11.8, 7.9 Hz, 1H), 1.20 (d, *J* = 6.3 Hz, 3H). ^13^C NMR (101 MHz, DMSO) δ 174.8, 168.4, 161.9, 134.7, 134.1, 133.7, 132.1, 128.4, 127.9, 127.7, 127.4, 127.1, 125.7, 57.6, 56.9, 22.4. HRMS (ESI) calcd for C_18_H_15_ClN_4_O [M+H]^+^ 339.1007, found 339.1028.

**5-(4-(4,5-Dihydro-1H-imidazol-2-yl)phenyl)-3-(p-tolyl)-1,2,4-oxadiazole (5t).**

White powder, 39% (240 mg) yield, m.p. 246–247 °C. ^1^H NMR (400 MHz, DMSO) δ 8.25 (d, *J* = 8.3 Hz, 2H), 8.09 (d, *J* = 8.4 Hz, 2H), 8.01 (d, *J* = 8.1 Hz, 2H), 7.43 (d, *J* = 8.0 Hz, 2H), 7.12 (s, 1H), 3.88 (br s, 2H), 3.47 (br s, 2H), 2.42 (s, 3H). ^13^C NMR (126 MHz, DMSO) δ 175.5, 169.0, 163.3, 142.1, 135.6, 130.2, 128.6 128.2, 127.6, 125.3, 124.1, 21.5, 21.4. HRMS (ESI) calcd for C_16_H_18_N_4_O [M+H]^+^ 305.1397, found 305.1386.

### 3.3. Biological Evaluation

#### 3.3.1. In Vitro Antibacterial Activity

All the synthesized compounds **3a-h** and **5a-t** were evaluated for their in vitro antimicrobial activity against *Staphylococcus aureus* (ATCC 25923) and *Bacillus subtilis* (VCМ V3142D) as examples of Gram-positive bacteria, and *Escherichia coli* (ATCC 25922) and *Pseudomonas fluorescence* (Р218) as examples of Gram-negative bacteria. The most potent antibacterial agents of this study (**3d**, **3e**, **5g**, **5k**, **5r**, and **5s**) were further evaluated for activity against antibiotic-resistant strains *Escherichia coli* DH52 REF, *Escherichia coli* K802 Rif and clinical isolates of an antibiotic-resistant strain of *Enterobacter* spp. Overnight cultures were grown at 37 °C in Lysogeny broth (LB) and diluted to obtain an opacity equivalent to 0.5 on the McFarland scale. Screening vials were filled with solutions of the test compounds in 0.5% DMSO as prepared above with three replications for each treatment. Active pharmaceutical ingredient (API) pefloxacin (0.5–256 µg/mL) and 0.5% DMSO served as positive and negative controls, respectively. The entire vial was incubated at 35 ± 2 °C for 18 h. After incubation, the antibacterial activity of the test compounds was determined by measuring the absorption of the solution with a spectrophotometer at 500 nm.

#### 3.3.2. MIC Measurement

The MICs of the most active compounds were measured using the twofold serial broth dilution method. The test organisms were grown in suitable broth for 18 h at 37 °C. Twofold serial dilutions of solutions of the test compounds were prepared at 256, 128, 64, 32, 16, 8, 4, 2, 1, and 0.5 µg/mL. The tubes were then inoculated with the test microbe; each 5 mL received 0.1 mL of the above inoculums and were incubated at 37 °C. The vials were subsequently observed for the presence or absence of microbial growth. The MIC values of the prepared compounds are listed in Table 1.

### 3.4. Cell Viability Assay

Human cell lines were maintained at 37 °C in a humidified atmosphere containing air and 5% CO_2_ as previously described [31]. Pancreas ductal adenocarcinoma cells PANC-1 were obtained from Russian collection cell cultures at the RAS Institute of Cytology (Saint Petersburg, Russian). The cell line was grown in Dulbecco’s Modified Eagle’s Medium-F12 (BioloT) containing 10% (*v*/*v*) heat-inactivated fetal calf serum (FCS, HyClone Laboratories, UT, USA), 1% l-glutamine, 1% sodium pyruvate, 50 U/mL penicillin, and 50 μg/mL streptomycin (BioloT). The cytotoxicity of oxadiazoles and the prepared imidazolines was evaluated using a routine Colorimetric method with tetrazolium dye—3-(4,5-dimethylthiazol-2-yl)-2,5-diphenyltetrazolium bromide (MTT). The cell lines were incubated for 48 h with medium containing different concentrations of investigated compounds. Following treatment, Dulbecco’s Modified Eagle’s Medium-F12 (100 μL/ well) and 20 μL of a 2.5 mg/mL MTT solution were added, and cells were incubated for 1 h at 37 °C. The used cell density was 5 × 10^3^ cells/200 μL/well in 96-well microtiter plates. After aspiration of the supernatants, the MTT-formazan crystals formed by metabolically active cells were dissolved in dimethyl sulfoxide (100 μL/well), and the absorbance was measured at 540 nm and 690 nm in Varioskan LUX™ Multimode Microplate Reader (Thermo Scientific, Waltham, MA, USA). Values measured at 540 nm were subtracted for background correction at 690 nm, and the data were plotted as a percent of control DMSO-treated samples.

## 4. Conclusion

To sum up, herein we report antibacterial, anticancer, and MAO-inhibitory activity of newly synthesized, as well as earlier obtained in our group, 1,2,4-oxadiazole/2-imidazoline hybrids. Some of the investigated compounds exhibited attractive antibacterial profile reaching MIC values between 8 and 32 µg/mL which makes them undoubtedly promising for further evaluation. Several frontrunners (**3d**, **3e**, **5g**, **5k**, **5r**, and **5s**) have been studied in more details and showed pronounced bacteriostatic properties against drug-resistant strains, as well as substantial antiproliferative activity against pancreas ductal adenocarcinoma (PANC-1) cell line. In the meantime, type-selective MAO inhibitory profile revealed for the most active compounds is favorable for them to demonstrate good side-effect characteristics in further development. Finally, we strongly believe that this scaffold may gain the attention of medicinal chemists as easily accessible privileged structure serving as a starting point for novel libraries of biologically active compounds.

## Figures and Tables

**Table 1:** Antibacterial activity compounds **3a-h** and **5a-t**.

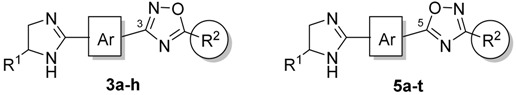							
**ID**	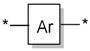	**R^1^**	**R^2^**	**MIC, µg/mL**			
				**Gram-positive**		**Gram-negative**	
				***S*** **.** ***aureus*** **(ATCC 25923)**	***B. Subtillis* (VCM V3142D)**	***E. Coli* (ATCC 25922)**	***P. fluorescens*** **(P218)**
**3a**	*p*-phenylene	H	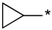	>256	>256	>256	>256
**3b**	*p*-phenylene	H	4-MeC_6_H_4_	>256	>256	>256	>256
**3c**	*p*-phenylene	H	Me	>256	>256	>256	>256
**3d**	*p*-phenylene	Me	3,4-diClC_6_H_3_	8	8	8	16
**3e**	*m*-phenylene	H	3-ClC_6_H_4_	128	32	128	64
**3f**	*m*-phenylene	H	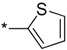	>256	>256	>256	>256
**3g**	*m*-phenylene	H	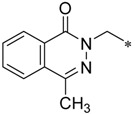	>256	>256	>256	>256
**3h**	*p*-phenylene	H	4-EtC_6_H_4_	256	>256	256	256
**5a**	*p*-phenylene	H	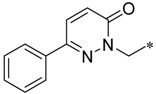	256	>256	256	>256
**5b**	*p*-phenylene	H	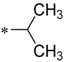	>256	>256	>256	>256
**5c**	*p*-phenylene	H	PhOCH_2_	256	>256	256	>256
**5d**	*p*-phenylene	H	4-*t*-BuC_6_H_4_	256	>256	256	>256
**5e**	*p*-phenylene	H	4-PhOC_6_H_4_	256	256	256	>256
**5f**	*p*-phenylene	H	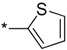	>256	>256	>256	>256
**5g**	*p*-phenylene	H	3-ClC_6_H_4_	16	8	16	32
**5h**	*p*-phenylene	H	2-ClC_6_H_4_	256	128	128	128
**5i**	*p*-phenylene	H	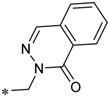	>256	>256	>256	>256
**5j**	*p*-phenylene	H	3,4-diMeOC_6_H_3_	>256	>256	>256	>256
**5k**	*p*-phenylene	H	3-MeOC_6_H_4_	128	64	32	128
**5l**	*p*-phenylene	H	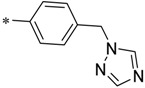	128	256	256	>256
**5m**	*p*-phenylene	H	4-MeC_6_H_4_CH_2_	>256	>256	>256	>256
**5n**	*p*-phenylene	H	4-PhC_6_H_4_	256	>256	256	>256
**5o**			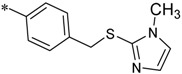	>256	>256	>256	>256
**5p**	*p*-phenylene	Me	3,4-diClC_6_H_3_	>256	>256	>256	>256
**5q**	*p*-phenylene	Me	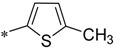	256	>256	256	>256
**5r**	*p*-phenylene	Me	4-MeC_6_H_4_	16	32	8	32
**5s**	*p*-phenylene	Me	3-ClC_6_H_4_	32	32	64	64
**5t**	*p*-phenylene	H	4-MeC_6_H_4_	>256	>256	>256	>256
Ref	Pefloxacine			0.008	<0.5	0.008	<0.5

* Measurements performed in triplicates. MIC: Minimal Inhibition Concentration; API: Active Pharmaceutical Ingredient.

**Table 2:** Antibacterial activity of compounds **3d**, **3e**, **5g**, **5k**, **5r**, and **5s**.

ID	MIC, µg/mL		
	*E. coli* (dhs2)	*E. coli* (k802)	*Enterobacter* spp.
**3d**	16	16	8
**3e**	64	64	32
**5g**	32	16	32
**5k**	>64	>64	>64
**5r**	32	64	64
**5s**	32	64	64
Pefloxacin	<0.5	<0.5	<0.5

* Measurements performed in triplicates. MIC: Minimal Inhibition Concentrations.

**Table 3:** The human monoamine oxidase (MAO) inhibition potencies of oxadiazole derivatives (**3d**, **3e**, **5g**, **5k**) and known antibiotics.

ID	IC_50_ (µM)	
	MAO-A	MAO-B
**3d**	14.7 ± 1.85	0.160 ± 0.0096
**3e**	1.39 ± 0.067	0.030 ± 0.0025
**5g**	5.47 ± 0.518	0.064 ± 0.0054
**5k**	12.0 ± 0.205	0.216 ± 0.019
Linezolid	46	2.1
Tedizolid	8.7	5.7

## Supplementary Materials

Supplementary materials can be found at https://www.mdpi.com/1422-0067/20/7/1699/s1.

## Abbreviations

MIC	Minimal Inhibition Concentrations
MAO	Monoamine Oxidase
MRSA	Methicillin-Resistant *Staphylococcus aureus*
VRE	Vancomycin-Resistant *Enterococcus*
API	Active Pharmaceutical Ingredient
