# Supplementary material for: 1,2,4-Oxadiazole/2-Imidazoline Hybrids: Multi-target-directed Compounds for the Treatment of Infectious Diseases and Cancer
Source: Int J Mol Sci. 2019 Apr 5;20(7):1699. doi: 10.3390/ijms20071699 (PMC6480344; doi:10.3390/ijms20071699)

SUPPLEMENTARY MATERIAL

1,2,4-Oxadiazole/2-imidazoline hybrids: Multi-target-directed compounds for the treatment of infectious diseases and cancer

Anton Shetnev [1], Sergey Baykov [2]*, Stanislav Kalinin [2], Alexandra Belova [1], Vladimir Sharoyko [2], Anton Rozhkov [2], Lev Zelenkov [2], Marina Tarasenko [3], Evgeny Sadykov [4], Mikhail Korsakov [1] and Mikhail Krasavin [2]

[1] Pharmaceutical Technology Transfer Center, Ushinsky Yaroslavl State Pedagogical University, 108 Respublikanskaya St., Yaroslavl 150000, Russian Federation; a.shetnev@yspu.org (A. Sh.); a.belova@yspu.org (A.B.); m.korsakov@yspu.org (M.K.)

[2] Institute of Chemistry, Saint Petersburg State University, 26 Universitetskii Prospect, Peterhof, Saint Petersburg 198504, Russian Federation; s.kalinin@spbu.ru (S.K.); v.sharoyko@spbu.ru (V.Sh.); a.rozhkov@spbu.ru (A.R.); chemleo@protonmail.ch (L.Z.); m.krasavin@spbu.ru (M.K.)

[3] Yaroslavl State Technical University, 88 Moscowsky Pr, Yaroslavl 150023, Russian Federation; mkarunnaya@mail.ru

[4] A.E. Favorsky Irkutsk Institute of Chemistry, Siberian Branch Russian Academy of Science, 1 Favorsky Str, Irkutsk 664033, Russian Federation; esadyk.chem@gmail.com

*Correspondence: s.baykov@spbu.ru

Content

S1. General information..3

S2. Synthesis and characterization of 1,2,4-oxadiazoles 4

S3. Copies of ^{1}H and ^{13}C NMR spectra 12

S1. General information

All reagents and solvents were obtained from commercial sources and were used without purification. DMSO was dried over molecular sieves (4 Å). Reactions were monitored by analytical thin layer chromatography (TLC) using Macherey-Nagel TLC sheets (Silufol UV-254) and the developed sheets were visualized under UV light. NMR spectra were recorded on Bruker AVANCE DPX 400 at 400 MHz and 101 MHz for ^1H and ^{13}C, respectively. Chemical shifts are reported as parts per million (δ, ppm) and were referenced to the residual solvent peaks at 7.26 and 2.50 ppm for ^1H in $CDCl_3$ and DMSO-d_6 respectively, and 39.52 and 77.16 ppm for ^{13}C in DMSO-d_6 and $CDCl_3$ respectively. Multiplicities are abbreviated as follows: s, singlet; d, doublet; t, triplet; q, quartet; m, multiplet; br, broad. Coupling constants, J, are reported in Hertz (Hz). Melting points were determined in open capillary tubes with an Electrothermal IA 9300 series Digital Melting Point Apparatus. High-resolution mass spectra (HRMS) were recorded with a Bruker maXis HRMS-ESI-QTOF (ESI mode).

S2. Synthesis and characterization of 1,2,4-oxadiazoles

General procedure for the synthesis of 3,5-disubstituted-1,2,4-oxadiazoles (3a-h, 5a-t)

To a solution of the amidoxime (2 mmol) and the appropriate carboxylic ester (2 mmol) in DMSO (1 mL), 120 mg (3 mmol) powdered NaOH was rapidly added. The reaction mixture was stirred at room temperature for the required time (TLC or precipitation of the product). The reaction mixture was diluted with cold water (30–50 mL). The resulting precipitate was collected by filtration, washed with water (30 mL), dried and purified by column chromatography (eluent: 5% methanol / 95% chloroform).

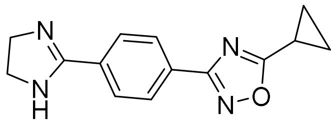

5-Cyclopropyl-3-(4-(4,5-dihydro-1*H*-imidazol-2-yl)phenyl)-1,2,4-oxadiazole (3a)

White solid, 89 % (391 mg) yield, m.p. 170–172 °C. ^{1}H NMR (DMSO-d_6, 300 MHz): δ 7.99 (q, J_1 = 8.6 Hz, J_2 = 14.9 Hz, 2H), 7.36 – 7.04 (br s, 1H), 3.64 (s, 4H), 2.46 – 2.37 (m, 1H), 1.26-1.33 (m, 2H), 1.23 – 1.16 (m, 2H). ^{13}C NMR (DMSO-d_6, 100 MHz): δ 182.5, 167.6, 163.4, 133.7, 128.3, 128.1, 127.3, 50.0, 10.5, 7.7. HRMS (ESI) calcd for $C_{14}H_{14}N_4O$ $[M+H]^+$ 255.1240, found 255.1253.

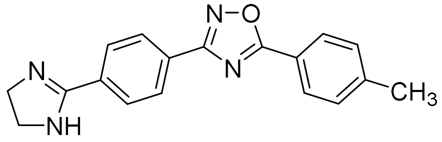

3-(4-(4,5-Dihydro-1*H*-imidazol-2-yl)phenyl)-5-p-tolyl-1,2,4-oxadiazole (3b)

White solid, 77 % yield, m.p. 184–186 °C. ^{1}H NMR (DMSO-d_6, 300 MHz): δ 8.11 (dd, J_1 = 7.8 Hz, J_2 = 16.4 Hz, 4H), 8.03 (d, J = 7.8 Hz, 2H), 7.48 (d, J = 7.3 Hz, 2H), 7.05 (s, 1H), 3.89 – 3.79 (m, 2H), 3.39-3.49 (m, 2H), 2.44 (s, 3H). ^{13}C NMR (DMSO-d_6, 100 MHz): δ 179.2, 175.0, 156.8, 142.6, 130.5, 130.2, 130.1, 127.4, 127.0, 126.9, 124.8, 119.5, 55.9, 32.0. HRMS (ESI) calcd for $C_{16}H_{18}N_4O$ $[M+H]^+$ 305.1397, found 305.1387.

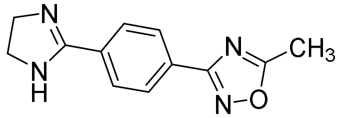

3-(4-(4,5-Dihydro-1*H*-imidazol-2-yl)phenyl)-5-methyl-1,2,4-oxadiazole (3c)

White solid, 63 % (156 mg) yield, m.p. 168–170 °C. ^{1}H NMR (DMSO-d_6, 300 MHz): δ 8.06 – 7.95 (m, 4H), 7.06 (s, 1H), 3.63 (s, 4H), 2.67 (s, 3H). ^{13}C NMR (DMSO-d_6, 100 MHz): δ 178.1, 167.7, 163.4, 133.8, 128.3, 128.2, 127.3, 55.6, 12.5. HRMS (ESI) calcd for $C_{12}H_{12}N_4O$ $[M+H]^+$ 229.1084, found 229.1093.

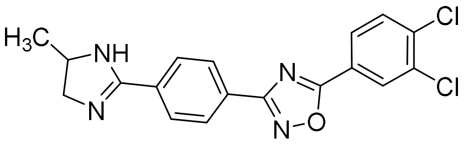

5-(3,4-Dichlorophenyl)-3-(4-(5-methyl-4,5-dihydro-1*H*-imidazol-2-yl)phenyl)-1,2,4-oxadiazole (3d)

White powder, 52 % (195 mg) yield, m.p. 190–192 °C. ^{1}H NMR (400 MHz, DMSO) δ 8.18 (m, 3H), 8.05 (d, *J* = 8.4 Hz, 2H), 8.00 (dd, *J* = 8.4, 1.9 Hz, 1H), 7.84 (d, *J* = 8.4 Hz, 1H), 4.09-3.99 (m, 1H), 3.85 – 3.77 (m, 1H), 3.24 (dd, *J* = 12.1, 7.9 Hz, 1H), 1.20 (d, *J* = 6.3 Hz, 3H). ^{13}C NMR (101 MHz, DMSO) δ 175.8, 167.1, 161.7, 135.4, 134.9, 132.7, 132.2, 129.1, 128.5, 128.3, 127.5, 127.0, 124.7, 58.0, 57.0, 22.3. HRMS (ESI) calcd for $C_{18}H_{14}Cl_2N_4O$ $[M+H]^+$ 373.0617, found 373.0633.

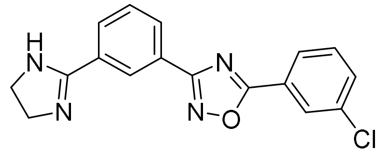

5-(3-Chlorophenyl)-3-(3-(4,5-dihydro-1*H*-imidazol-2-yl)phenyl)-1,2,4-oxadiazole (3e)

White powder, 78 % (271 mg) yield, m.p. 210–211 °C. ^{1}H NMR (400 MHz, DMSO) δ 8.58 (s, 1H), 8.16 (m, 3H), 8.05 (d, *J* = 7.2 Hz, 1H), 7.79 (d, *J* = 7.1 Hz, 1H), 7.72-7.63 (m, 7.6 Hz, 2H), 6.87 (s, 1H), 3.68 (s, 4H). ^{13}C NMR (101 MHz, DMSO) δ 174.9, 168.8, 163.4, 134.8, 133.5, 132.4, 131.9, 130.6, 129.6, 129.1, 127.9, 127.1, 126.6, 126.5, 125.9. HRMS (ESI) calcd for $C_{17}H_{13}ClN_4O$ $[M+H]^+$ 325.0851, found 325.0874.

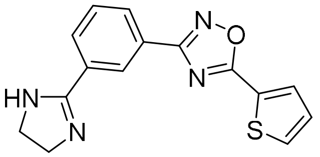

3-(3-(4,5-Dihydro-1*H*-imidazol-2-yl)phenyl)-5-(thiophen-2-yl)-1,2,4-oxadiazole (3f)

White solid, 79 % (302 mg) yield, m.p. 215–217 °C. ^{1}H NMR (400 MHz, DMSO) δ 8.53 (s, 1H), 8.16 – 8.10 (m, 3H), 8.03 (d, J = 7.9 Hz, 1H), 7.65 (t, J = 7.8 Hz, 1H), 7.44 – 7.33 (m, 1H), 7.26 (s, 1H), 3.65 (s, 4H). ^{13}C NMR (101 MHz, DMSO) δ 171.7, 168.3, 163.4, 134.7, 133.4, 132.0, 130.6, 129.8, 129.2, 126.4, 126.4, 125.7, 124.9, 50.0. HRMS (ESI) calcd for $C_{15}H_{12}N_4OS$ $[M+H]^+$ 297.0805, found 297.0807.

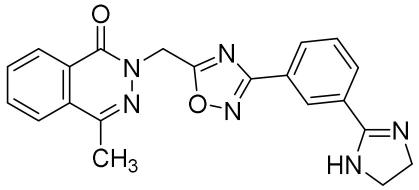

2-((3-(3-(4,5-Dihydro-1*H*-imidazol-2-yl)phenyl)-1,2,4-oxadiazol-5-yl)methyl)-4-methylphthalazin-1(2*H*)-one (3g)

White powder, 57 % (230 mg) yield, m.p. 151–153 °C. ^{1}H NMR (400 MHz, DMSO) δ 8.44 (s, 1H), 8.33 (d, *J* = 7.8 Hz, 1H), 8.08 – 7.95 (m, 4H), 7.94 – 7.88 (m, 1H), 7.59 (t, *J* = 7.8 Hz, 1H), 5.71 (s, 2H), 3.64 (s,

4H), 2.59 (s, 3H). ^{13}C NMR (101 MHz, DMSO) δ 176.4, 168.2, 163.4, 159.0, 145.1, 134.3, 132.6, 132.4, 130.5, 130.1, 129.6, 129.0, 127.3, 126.8, 126.5, 126.4, 126.3, 47.2, 41.3, 18.8. HRMS (ESI) calcd for $C_{21}H_{18}N_6O_2$ $[M+H]^+$ 387.1564, found 387.1589.

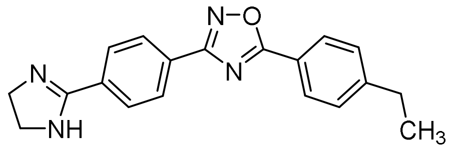

3-(4-(4,5-Dihydro-1H-imidazol-2-yl)phenyl)-5-(4-ethylphenyl)-1,2,4-oxadiazole (3h)

White powder, 34 % (216 mg) yield, m.p. 196–198 °C. ^{1}H NMR (400 MHz, DMSO) δ 8.14 (m, 4H), 8.04 (d, *J* = 8.4 Hz, 2H), 7.53 (d, *J* = 8.2 Hz, 2H), 7.07 (br s, 1H), 3.66 (s, 4H), 2.75 (q, *J* = 7.6 Hz, 2H), 1.25 (t, *J* = 7.6 Hz, 3H). ^{13}C NMR (101 MHz, DMSO) δ 176.1, 168.3, 163.4, 150.4, 133.9, 129.5, 128.5, 128.4, 128.1, 127.4, 121.3, 28.7, 15.5. HRMS (ESI) calcd for $C_{19}H_{18}N_4O$ $[M+H]^+$ 319.1553, found 319.1573.

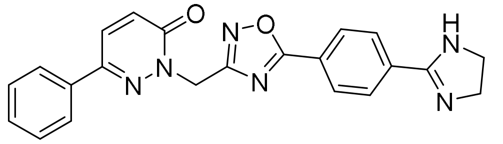

2-((5-(4-(4,5-Dihydro-1*H*-imidazol-2-yl)phenyl)-1,2,4-oxadiazol-3-yl)methyl)-6-phenylpyridazin-3(2*H*)-one (5a)

Light brown solid, 82 % (256 mg) yield, m.p. 216–218 °C. ^{1}H NMR (DMSO-d_6, 300 MHz): δ 8.13 (dd, *J*= 6.16 Hz, *J*= 8.8 Hz, 3H), 8.02 (d, *J* = 8.3 Hz, 2H), 7.88 (d, *J* = 6.6 Hz, 2H), 7.53 – 7.42 (m, 3H), 7.16 (d, *J* = 9.8 Hz, 1H), 5.59 (s, 2H, CH_2), 3.64 (s, 4H). ^{13}C NMR (DMSO-d_6, 100 MHz): δ 175.7, 167.7, 163.2, 159.2, 144.8, 135.5, 134.7, 131.9, 130.4, 130.0, 129.3, 128.6, 128.2, 126.4, 124.9, 50.3, 47.5. Anal. Calcd for $C_{22}H_{18}N_6O_2$ (398.43): C, 66.32; H, 4.55; N, 21.09; found: C, 66.12; H, 4.56; N, 20.99.

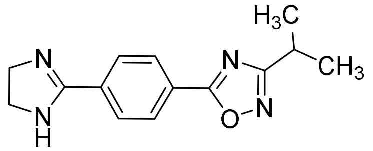

5-(4-(4,5-Dihydro-1*H*-imidazol-2-yl)phenyl)-3-isopropyl-1,2,4-oxadiazole (5b)

White solid, 77 % (337 mg) yield, m.p. 164–166 °C. ^{1}H NMR (DMSO-d_6, 300 MHz): δ 8.12 (d, *J* = 8.3 Hz, 2H), 8.03 (d, *J* = 8.3 Hz, 2H), 3.64 (s, 4H), 3.19 – 3.08 (m, 1H), 1.32 (d, *J* = 7.1 Hz, 6H). ^{13}C NMR (DMSO-d_6, 75 MHz): δ 175.8, 174.8, 163.2, 135.0, 128.5, 128.1, 125.2, 50.2, 26.6. 20.8. Anal. Calcd for $C_{14}H_{16}N_4O$ (256.31): C, 65.61; H, 6.29; N, 21.86; found: C, 65.41; H, 6.30; N, 21.75.

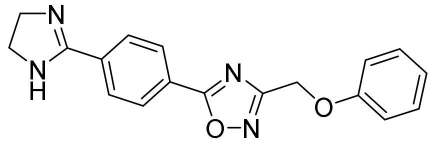

5-(4-(4,5-Dihydro-1*H*-imidazol-2-yl)phenyl)-3-(phenoxymethyl)-1,2,4-oxadiazole (5c)

White solid, 64 % (278 mg) yield, m.p. 190–192 °C. ^{1}H NMR (DMSO-d_6, 300 MHz): δ 8.18 (d, J = 7.8 Hz, 2H), 8.05 (d, J = 7.3 Hz, 2H), 7.38 – 7.28 (m, 2H), 7.08 (d, J = 8.1 Hz, 2H), 6.95-7.04 (m, 1H), 5.36 (s, 2H), 3.92 – 3.73 (br s, 2H), 3.36-3.55 (br s, 2H). ^{13}C NMR (DMSO-d_6, 75 MHz): δ 175.6, 168.1, 163.2, 158.1, 135.4, 130.1, 128.6, 128.3, 124.8, 124.7, 122.0, 115.3, 61.2. Anal. Calcd for $C_{18}H_{16}N_4O_2$ (320.35): C, 67.49; H, 5.03; N, 17.49; found: C, 67.29; H, 5.04; N, 17.58.

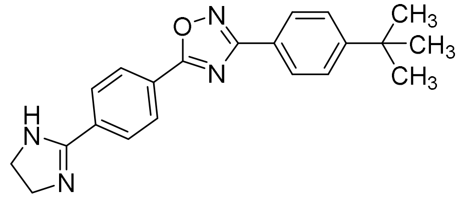

3-(4-*Tert*-butylphenyl)-5-(4-(4,5-dihydro-1*H*-imidazol-2-yl)phenyl)-1,2,4-oxadiazole (5d)

White solid, 79 % (338 mg) yield, m.p. 196–198 °C. ^{1}H NMR (DMSO-d_6, 300 MHz): δ 8.23 (d, J = 8.3 Hz, 2H), 8.10 – 8.00 (m, 4H), 7.62 (d, J = 8.3 Hz, 2H), 7.12 (s, 1H), 3.91 – 3.79 (m, 2H), 3.40-3.51 (m, 2H), 1.33 (s, 9H). ^{13}C NMR (DMSO-d_6, 100 MHz): δ 175.3, 168.7, 163.2, 155.0, 135.3, 128.6, 128.3, 127.4, 126.6, 125.1, 123.8, 55.2, 35.2, 31.4. Anal. Calcd for $C_{21}H_{22}N_4O$ (346.44): C, 72.81; H, 6.40; N, 16.17; found: C, 72.59; H, 6.41; N, 16.09.

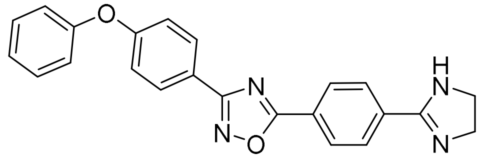

5-(4-(4,5-Dihydro-1*H*-imidazol-2-yl)phenyl)-3-(4-phenoxyphenyl)-1,2,4-oxadiazole (5e)

White powder, 74 % (363 mg) yield, m.p. 241–242 °C. ^{1}H NMR (400 MHz, DMSO) δ 8.41 (d, J = 8.4 Hz, 2H), 8.34 (d, J = 8.4 Hz, 2H), 8.11 (d, J = 8.7 Hz, 2H), 7.48 (t, J = 7.9 Hz, 2H), 7.25 (t, J = 7.4 Hz, 1H), 7.17 (dd, J = 11.9, 8.4 Hz, 4H), 4.05 (s, 4H). ^{13}C NMR (101 MHz, DMSO) δ 174.6, 168.5, 164.4, 160.5, 155.8, 130.8, 130.3, 129.8, 129.0, 128.5, 126.6, 125.1, 120.9, 120.3, 118.8, 45.0. HRMS (ESI) calcd for $C_{23}H_{18}N_4O_2$ $[M+H]^+$ 383.1503, found 383.1505.

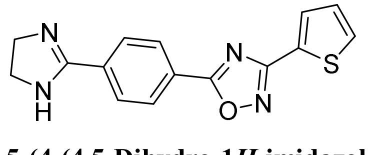

5-(4-(4,5-Dihydro-1*H*-imidazol-2-yl)phenyl)-3-(thiophen-2-yl)-1,2,4-oxadiazole (5f)

White powder, 84 % (320 mg) yield, m.p. 182–184 °C. ^{1}H NMR (400 MHz, DMSO) δ 8.07 (m, 6H), 7.37 (t, J = 4.1 Hz, 1H), 3.67 (s, 4H). ^{13}C NMR (101 MHz, DMSO) δ 171.8, 168.4, 163.5, 134.4, 134.2, 133.2, 129.6, 128.4, 127.9, 127.4, 125.1, 50.3. HRMS (ESI) calcd for $C_{15}H_{12}N_4OS$ $[M+H]^+$ 297.0805, found 297.0806.

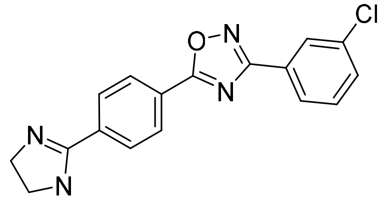

3-(3-Chlorophenyl)-5-(4-(4,5-dihydro-1*H*-imidazol-2-yl)phenyl)-1,2,4-oxadiazole (5g)

White solid, 62 % (258 mg) yield, m.p. 187–188 °C. ^{1}H NMR (400 MHz, DMSO) δ 8.25 (d, *J* = 8.3 Hz, 1H), 8.15 – 7.99 (m, 2H), 7.72 (d, *J* = 8.3 Hz, 1H), 7.65 (t, *J* = 7.8 Hz, 1H), 7.17 (s, 1H), 3.66 (s, 2H). ^{13}C NMR (101 MHz, DMSO) δ 179.6, 175.8, 173.8, 167.8, 135.3, 134.5, 134.3, 134.1, 131.9, 128.6, 128.5, 127.2, 126.3, 125.1, 50.1. HRMS (ESI) calcd for $C_{20}H_{16}N_6O_2$ $[M+H]^+$ 325.0851, found 325.0856.

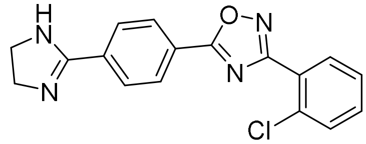

3-(2-Chlorophenyl)-5-(4-(4,5-dihydro-1*H*-imidazol-2-yl)phenyl)-1,2,4-oxadiazole (5h)

White powder, 79 % (329 mg) yield, m.p. 179–181 °C. ^{1}H NMR (400 MHz, DMSO) δ 8.24 (d, *J* = 8.3 Hz, 1H), 8.08 (d, *J* = 8.3 Hz, 1H), 8.01 (d, *J* = 6.4 Hz, 1H), 7.72 (d, *J* = 7.8 Hz, 1H), 7.64 (t, *J* = 6.9 Hz, 1H), 7.58 (t, *J* = 7.5 Hz, 1H), 7.13 (s, 1H), 3.84 (s, 1H), 3.46 (s, 1H).^{13}C NMR (101 MHz, DMSO) δ 175.0, 167.7, 163.2, 135.4, 133.2, 132.7, 132.3, 131.3, 128.6, 128.4, 128.2, 125.9, 124.8, 40.9. HRMS (ESI) calcd for $C_{17}H_{13}ClN_4O$ $[M+H]^+$ 325.0851, found 325.0849.

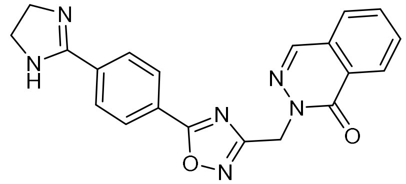

2-((5-(4-(4,5-Dihydro-1*H*-imidazol-2-yl)phenyl)-1,2,4-oxadiazol-3-yl)methyl)phthalazin-1(2*H*)-one (5i)

White solid, 87 % (361 mg) yield, m.p. 229–230 °C. ^{1}H NMR (400 MHz, DMSO) δ δ 8.46 (s, 1H), 8.31 (d, J = 7.9 Hz, 1H), 8.09 (d, J = 8.5 Hz, 2H), 8.02 (d, J = 8.3 Hz, 2H), 7.97 (t, J = 2.7 Hz, 2H), 7.93 – 7.87 (m, 1H), 6.85 (s, 1H, NH), 5.58 (s, 2H, CH2), 3.65 (s, 4H). ^{13}C NMR (101 MHz, DMSO) δ 175.6, 168.1, 163.2, 159.0, 139.1, 135.6, 134.3, 132.7, 130.1, 128.5, 128.2, 127.5, 126.4, 124.9, 46.5. HRMS (ESI) calcd for $C_{20}H_{16}N_6O_2$ $[M+H]^+$ 322.1408, found 321.1307.

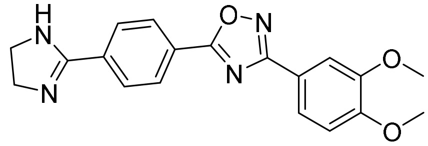

5-(4-(4,5-Dihydro-1*H*-imidazol-2-yl)phenyl)-3-(3,4-dimethoxyphenyl)-1,2,4-oxadiazole (5j)

White solid, 59 % (223 mg) yield, m.p. 194–195 °C. ^{1}H NMR (400 MHz, DMSO) δ 8.21 (d, J = 8.0 Hz, 2H), 8.06 (d, J = 8.1 Hz, 2H), 7.68 (d, J = 8.3 Hz, 2H), 7.55 (s, 1H), 7.14 (d, J = 8.6 Hz, 2H), 3.87 - 3.76 (m, 8H), 3.46 (s, 2H). ^{13}C NMR (75 MHz, DMSO) δ 175.1, 168.6, 163.2, 152.1, 149.5, 135.2, 128.5, 128.3, 125.1, 121.0, 118.8, 112.4, 110.2, 56.1, 55.2, 45.0. HRMS (ESI) calcd for $C_{19}H_{18}N_4O_3$ $[M+H]^+$ 351.1452, found 351.1454.

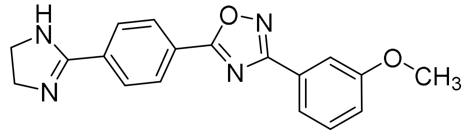

5-(4-(4,5-Dihydro-1*H*-imidazol-2-yl)phenyl)-3-(3-methoxyphenyl)-1,2,4-oxadiazole (5k)

White solid, 72 % (296 mg) yield, m.p. 194–195 °C. ^{1}H NMR (400 MHz, DMSO) δ 8.22 (d, *J* = 8.1 Hz, 2H), 8.09 (d, *J* = 8.1 Hz, 2H), 7.69 (d, *J* = 7.6 Hz, 1H), 7.61 (d, *J* = 2.6 Hz, 1H), 7.52 (t, *J* = 8.0 Hz, 1H), 7.19 (dd, *J* = 8.3, 2.7 Hz, 1H), 6.89 (s, 1H), 3.68 (s, 4H). ^{13}C NMR (101 MHz, DMSO) δ 190.3, 175.6, 168.9, 163.2, 160.4, 135.6, 130.9, 128.6, 128.3, 128.0, 125.1, 120.0, 118.1, 112.9, 56.0, 47.4. HRMS (ESI) calcd for C18H16N4O2 [M+H] 321.1346, found 321.1349.

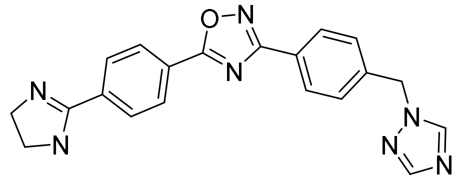

3-(4-((1*H*-1,2,4-Triazol-1-yl)methyl)phenyl)-5-(4-(4,5-dihydro-1*H*-imidazol-2-yl)phenyl)-1,2,4-oxadiazole (5l)

White powder, 33 % (130 mg) yield, m.p. 243–245 °C. ^{1}H NMR (400 MHz, DMSO) δ 8.65 (s, 1H), 8.22 (d, *J* = 8.3 Hz, 2H), 8.09 (d, *J* = 8.2 Hz, 4H), 7.99 (s, 1H), 7.50 (d, *J* = 8.1 Hz, 2H), 5.54 (s, 2H), 3.70 (s, 4H). ^{13}C NMR (101 MHz, DMSO) δ 175.6, 168.7, 163.3, 152.3, 144.9, 140.3, 135.2, 129.0, 128.6, 128.3, 128.0, 126.4, 125.3, 52.4, 50.1. HRMS (ESI) calcd for $C_{20}H_{17}N_7O$ $[M+H]^+$ 372.1567, found 372.1579.

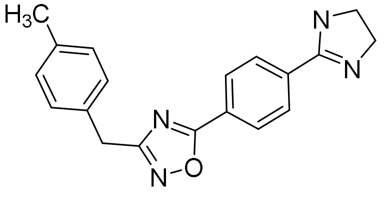

5-(4-(4,5-Dihydro-1*H*-imidazol-2-yl)phenyl)-3-(4-methylbenzyl)-1,2,4-oxadiazole (5m)

White powder, 65 % (225 mg) yield, m.p. 181–183 °C. ^{1}H NMR (400 MHz, DMSO) δ 8.11 (d, J = 8.5 Hz, 2H), 8.04 (d, J = 8.4 Hz, 2H), 7.25 (d, J = 7.8 Hz, 2H), 7.15 (d, J= 7.8 Hz, 2H), 4.12 (s, 2H), 3.69 (s, 4H), 2.29 (s, 3H). ^{13}C NMR (101 MHz, DMSO) δ 175.1, 170.7, 163.3, 136.5, 134.8, 133.1, 129.6, 129.2, 128.6, 128.1, 125.4, 50.0, 31.6, 21.0. HRMS (ESI) calcd for $C_{19}H_{18}N_4O$ $[M+H]^+$ 319.1554, found 319.1544.

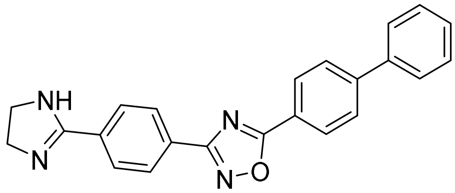

5-([1,1'-Biphenyl]-4-yl)-3-(4-(4,5-dihydro-1*H*-imidazol-2-yl)phenyl)-1,2,4-oxadiazole (5n)

White solid, 82 % (385 mg) yield, m.p. 238–240 °C. ^{1}H NMR (400 MHz, DMSO) δ 8.44 (d, *J* = 6.3 Hz, 2H), 8.36 (d, *J* = 5.9 Hz, 2H), 8.20 (d, *J* = 6.9 Hz, 2H), 7.92 (d, *J* = 6.7 Hz, 2H), 7.77 (d, *J* = 5.8 Hz, 2H), 7.61 – 7.48 (m, 2H), 7.45 (d, *J* = 6.1 Hz, 1H), 4.07 (s, 4H). ^{13}C NMR (101 MHz, DMSO) δ 172.0, 164.8, 161.4, 130.3, 129.9, 129.5, 129.3, 129.2, 129.0, 128.7, 128.6, 128.3, 128.0, 127.3, 125.4, 45.2. HRMS (ESI) calcd for $C_{23}H_{18}N_4O$[M+H]$^+$ 367.1554, found 367.1555.

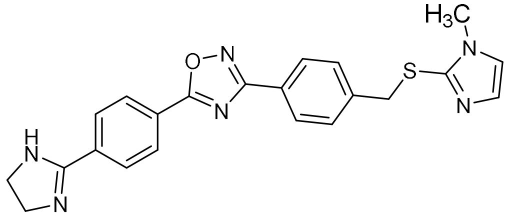

5-(4-(4,5-Dihydro-1*H*-imidazol-2-yl)phenyl)-3-(4-(((1-methyl-1*H*-imidazol-2-yl)thio)methyl)phenyl)-1,2,4-oxadiazole (5o)

White powder, 54 % (293 mg) yield, m.p. 166–168 °C. ^{1}H NMR (400 MHz, DMSO) δ 8.23 (d, *J* = 8.3 Hz, 2H), 8.08 (d, *J* = 8.3 Hz, 2H), 7.99 (d, *J* = 8.1 Hz, 2H), 7.40 (d, *J* = 8.1 Hz, 2H), 7.23 (s, 1H), 7.00 (s, 1H), 4.26 (s, 2H), 3.67 (s, 4H), 3.39 (s, 3H). ^{13}C NMR (101 MHz, DMSO) δ 175.4, 168.6, 163.2, 142.6, 139.4, 135.2, 130.1, 129.4, 128.6, 128.3, 127.6, 125.3, 125.0, 124.0, 38.6, 33.3. HRMS (ESI) calcd for $C_{22}H_{20}N_6OS$ [M+H]$^+$ 417.1492, found 417.1507.

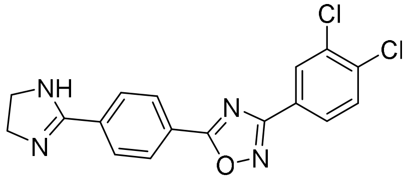

5-(3,4-Dichlorophenyl)-3-(4-(4,5-dihydro-1*H*-imidazol-2-yl)phenyl)-1,2,4-oxadiazole (5p)

Light brown crystal, 64 % (299 mg) yield, m.p. 271–273 °C. ^{1}H NMR (400 MHz, DMSO) δ 8.25 (d, *J* = 7.3 Hz, 2H), 8.12 – 7.99 (m, 2H), 7.90 (d, *J* = 8.4 Hz, 2H), 7.20 (s, 1H), 3.66 (s, 4H). ^{13}C NMR (101 MHz, DMSO) δ 175.4, 168.6, 163.3, 142.6, 139.4, 134.5, 134.1, 130.1, 129.4, 128.7, 128.4, 127.6, 125.3, 124.0, 49.8. HRMS (ESI) calcd for $C_{17}H_{12}Cl_2N_4O$ [M+H]$^+$ 359.0461, found 359.0461.

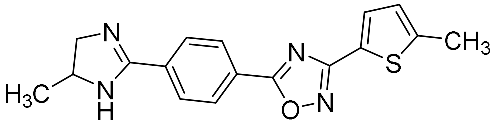

5-(4-(5-Methyl-4,5-dihydro-1*H*-imidazol-2-yl)phenyl)-3-(5-methylthiophen-2-yl)-1,2,4-oxadiazole (5q)

Pale powder, 61 % (201 mg) yield, m.p. 159–161 °C. ^{1}H NMR (400 MHz, DMSO) δ 8.18 (d, *J* = 8.4 Hz, 2H), 8.06 (d, *J* = 8.4 Hz, 2H), 7.69 (d, *J* = 3.6 Hz, 1H), 7.19 (s, 1H), 6.99 (dd, *J* = 3.7, 1.4 Hz, 1H), 4.18 – 3.65 (m, 2H), 2.56 – 2.52 (m, 3H), 1.18 (d, *J* = 6.0 Hz, 3H). ^{13}C NMR (101 MHz, DMSO) δ 175.2, 164.8, 161.7, 145.3, 135.5, 130.9, 128.5, 128.3, 127.5, 125.2, 124.8, 51.8, 22.4, 15.6. HRMS (ESI) calcd for $C_{17}H_{16}N_4OS$ $[M+H]^+$ 325.1118, found 325.1119.

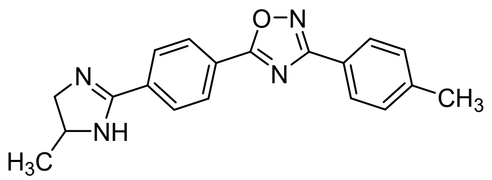

5-(4-(5-Methyl-4,5-dihydro-1H-imidazol-2-yl)phenyl)-3-(p-tolyl)-1,2,4-oxadiazole (5r)

Pale powder, 59 % (330 mg) yield, m.p. 89–90 °C. ^{1}H NMR (400 MHz, DMSO) δ 8.23 (d, *J* = 8.4 Hz, 2H), 8.08 (d, *J* = 8.4 Hz, 2H), 7.99 (d, *J* = 8.1 Hz, 2H), 7.41 (d, *J* = 8.0 Hz, 2H), 7.22 (br s, 1H), 4.09 – 3.98 (m, 1H), 3.81 (t, *J* = 11.1 Hz, 1H), 3.24 (dd, *J* = 11.8, 7.9 Hz, 1H), 1.20 (d, *J* = 6.3 Hz, 3H). ^{13}C NMR (101 MHz, DMSO) δ 175.3, 168.8, 161.7, 142.1, 135.3, 130.3, 128.5, 128.3, 127.5, 125.0, 123.8, 22.4, 21.6. HRMS (ESI) calcd for $C_{19}H_{18}N_4O$ $[M+H]^+$ 319.1553, found 319.1555.

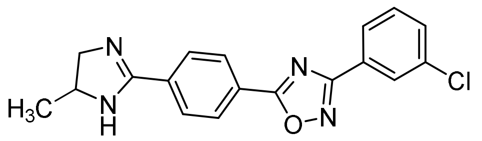

3-(3-chlorophenyl)-5-(4-(5-methyl-4,5-dihydro-1H-imidazol-2-yl)phenyl)-1,2,4-oxadiazole (5s)

White powder, 36 % (243 mg) yield, m.p. 139–141 °C. ^{1}H NMR (400 MHz, DMSO) δ 8.16 (m, 4H), 8.03 (d, *J* = 8.2 Hz, 2H), 7.83 (d, *J* = 8.1 Hz, 1H), 7.71 (t, *J* = 7.9 Hz, 1H), 7.18 (br s, 1H), 4.09 – 3.96 (m, 1H), 3.81 (t, *J* = 11.0 Hz, 1H), 3.23 (dd, *J* = 11.8, 7.9 Hz, 1H), 1.20 (d, *J* = 6.3 Hz, 3H). ^{13}C NMR (101 MHz, DMSO) δ 174.8, 168.4, 161.9, 134.7, 134.1, 133.7, 132.1, 128.4, 127.9, 127.7, 127.4, 127.1, 125.7, 57.6, 56.9, 22.4. HRMS (ESI) calcd for $C_{18}H_{15}ClN_4O$ $[M+H]^+$ 339.1007, found 339.1028.

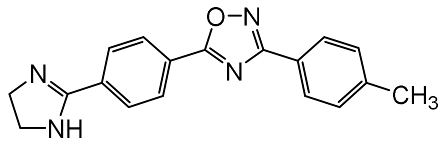

5-(4-(4,5-dihydro-1H-imidazol-2-yl)phenyl)-3-(p-tolyl)-1,2,4-oxadiazole (5t).

White powder, 39 % (240 mg) yield, m.p. 246–247 °C. ^{1}H NMR (400 MHz, DMSO) δ 8.25 (d, *J* = 8.3 Hz, 2H), 8.09 (d, *J* = 8.4 Hz, 2H), 8.01 (d, *J* = 8.1 Hz, 2H), 7.43 (d, *J* = 8.0 Hz, 2H), 7.12 (s, 1H), 3.88 (br. s, 2H), 3.47 (br s, 2H), 2.42 (s, 3H). ^{13}C NMR (126 MHz, DMSO) δ 175.5, 169.0, 163.3, 142.1, 135.6, 130.2, 128.6, 128.2, 127.6, 125.3, 124.1, 21.5, 21.4. HRMS (ESI) calcd for $C_{16}H_{18}N_4O$ $[M+H]^+$ 305.1397, found 305.1386.

S3. Copies of ^{1}H and ^{13}C NMR spectra

^{1}H and ^{13}C NMR spectra of 5-cyclopropyl-3-(4-(4,5-dihydro-1*H*-imidazol-2-yl)phenyl)-1,2,4-oxadiazole (**3a**)

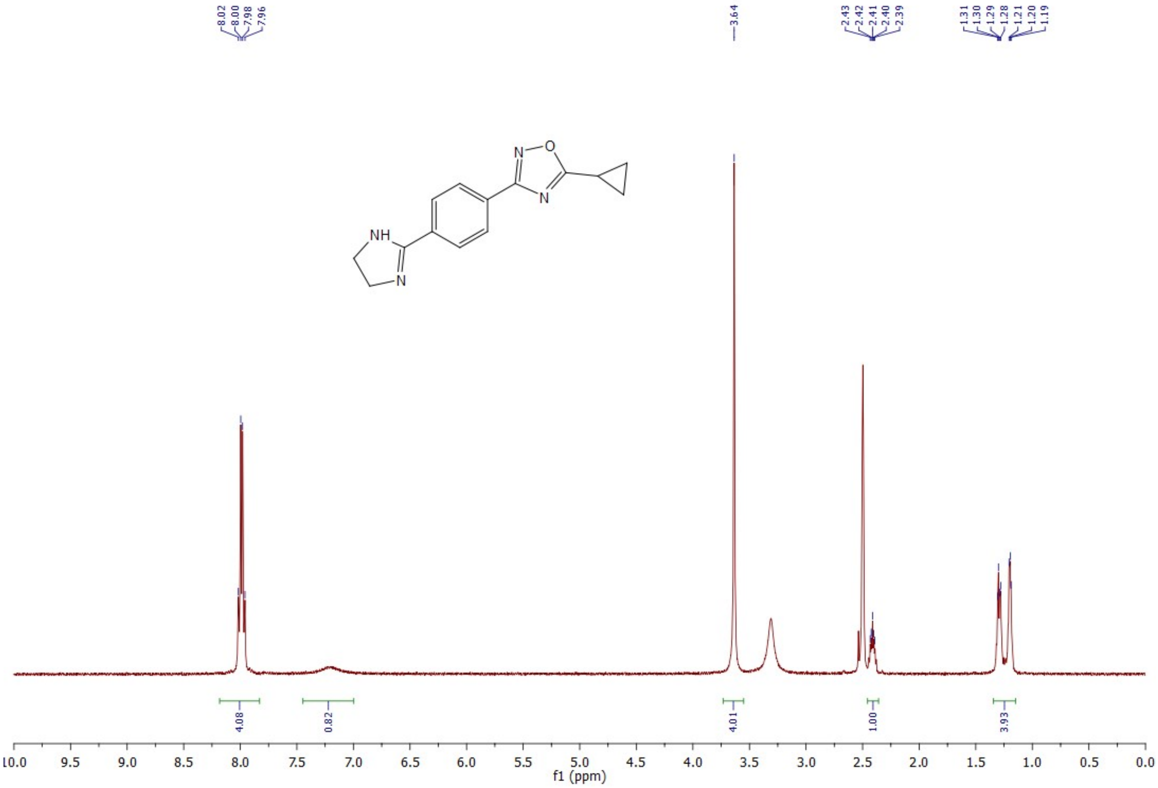

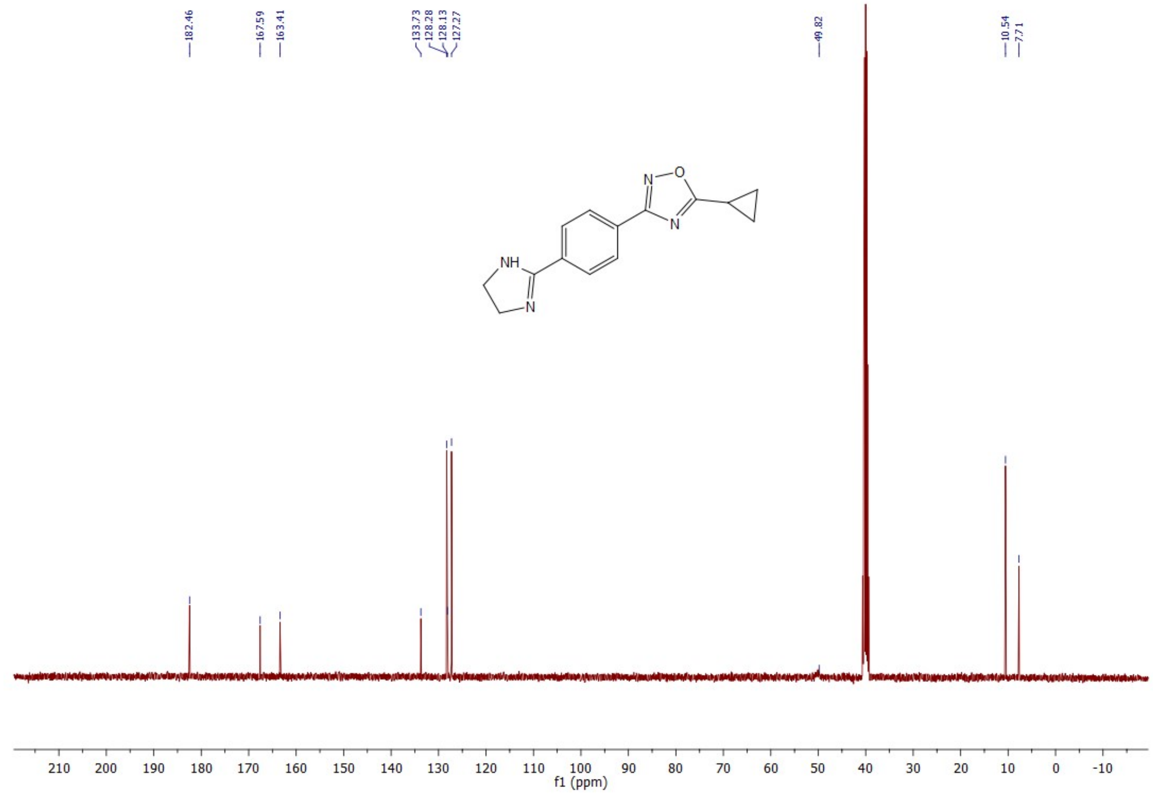

^{1}H and ^{13}C NMR spectra of 3-(4-(4,5-Dihydro-1*H*-imidazol-2-yl)phenyl)-5-p-tolyl-1,2,4-oxadiazole (**3b**)

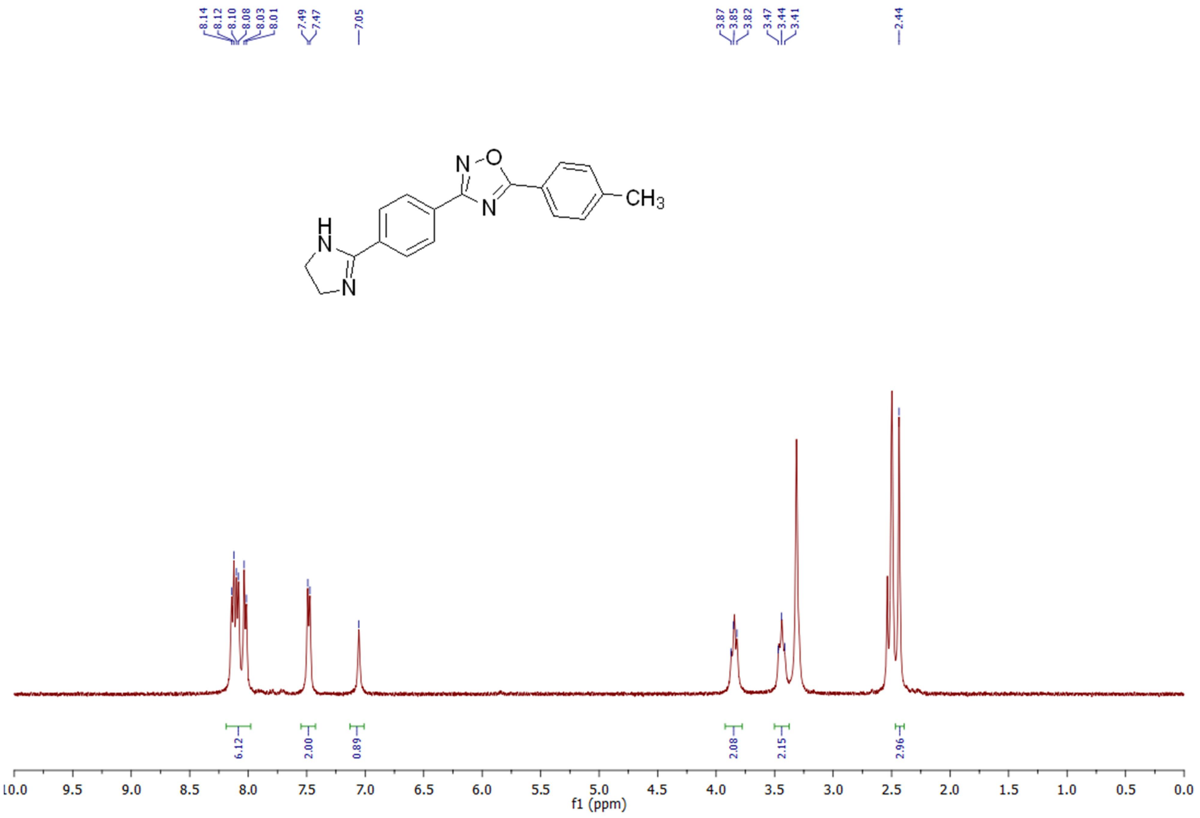

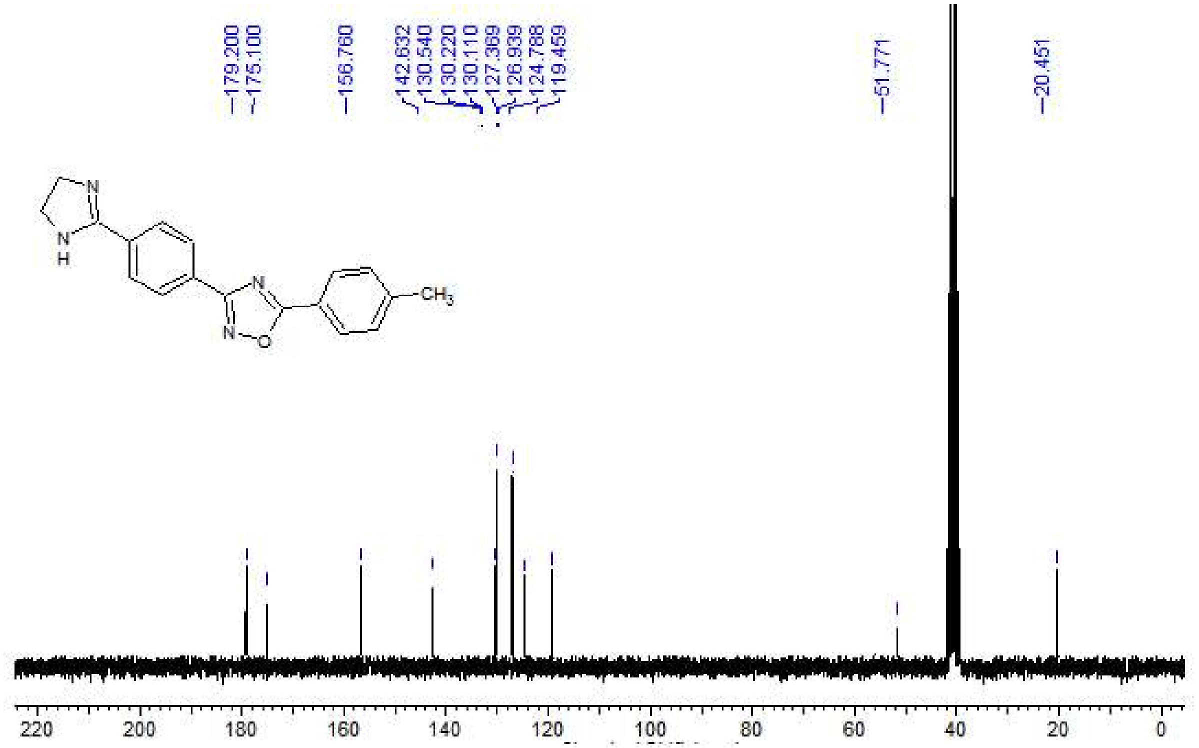

^{1}H and ^{13}C NMR spectra of 3-(4-(4,5-dihydro-1*H*-imidazol-2-yl)phenyl)-5-methyl-1,2,4-oxadiazole (**3c**)

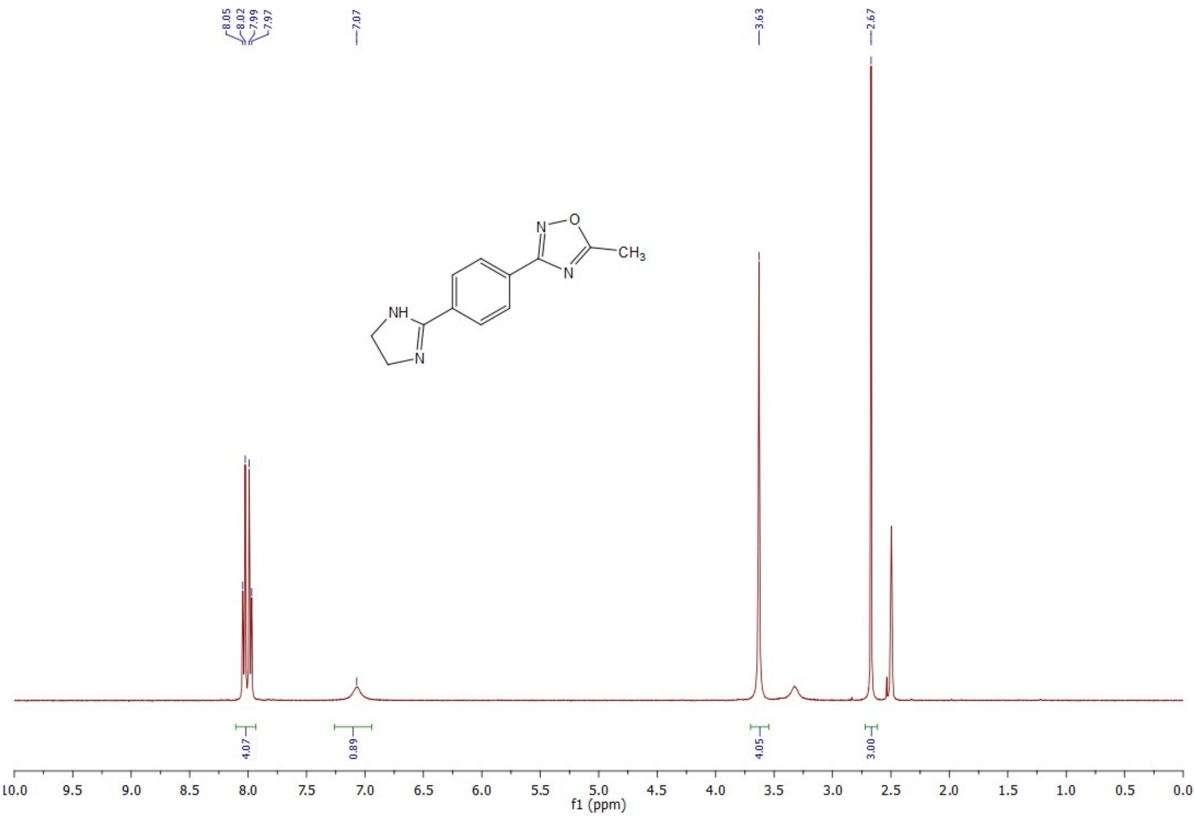

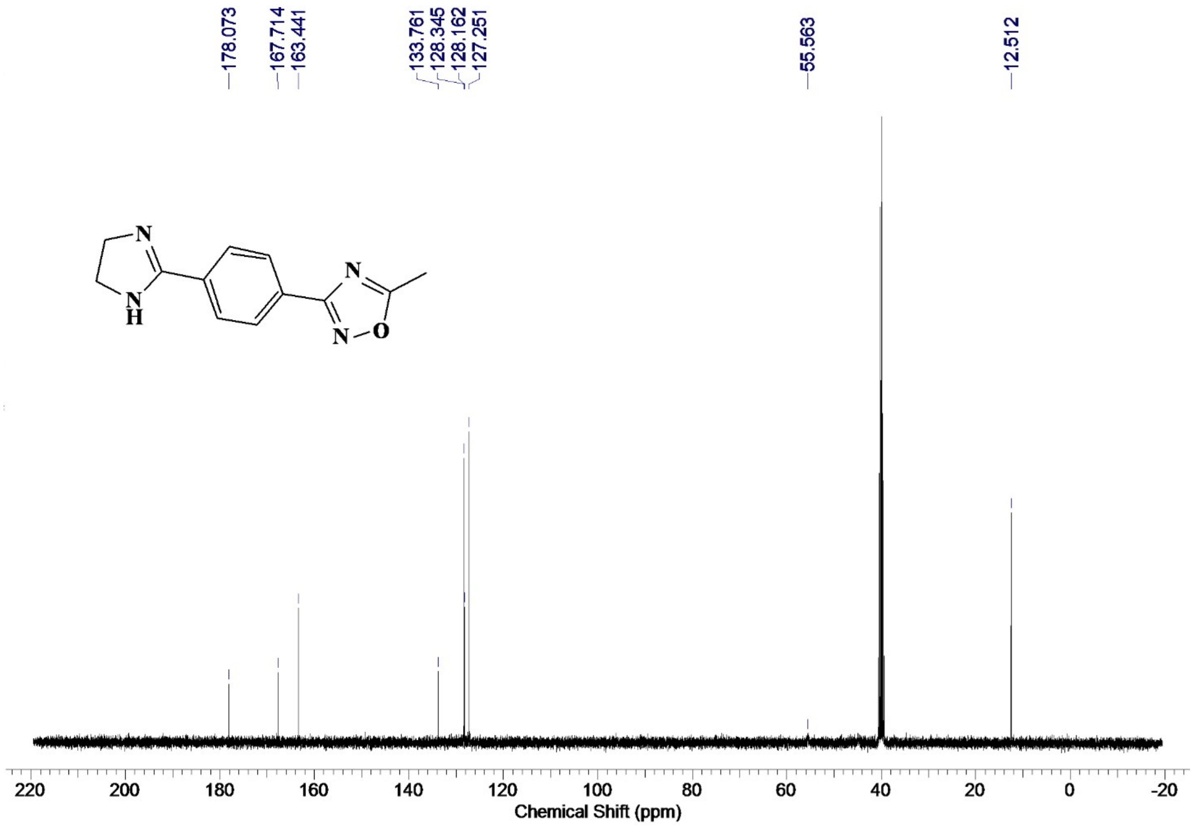

^{1}H and ^{13}C NMR spectra of 5-(3,4-dichlorophenyl)-3-(4-(5-methyl-4,5-dihydro-1*H*-imidazol-2-yl)phenyl)-1,2,4-oxadiazole (**3d**)

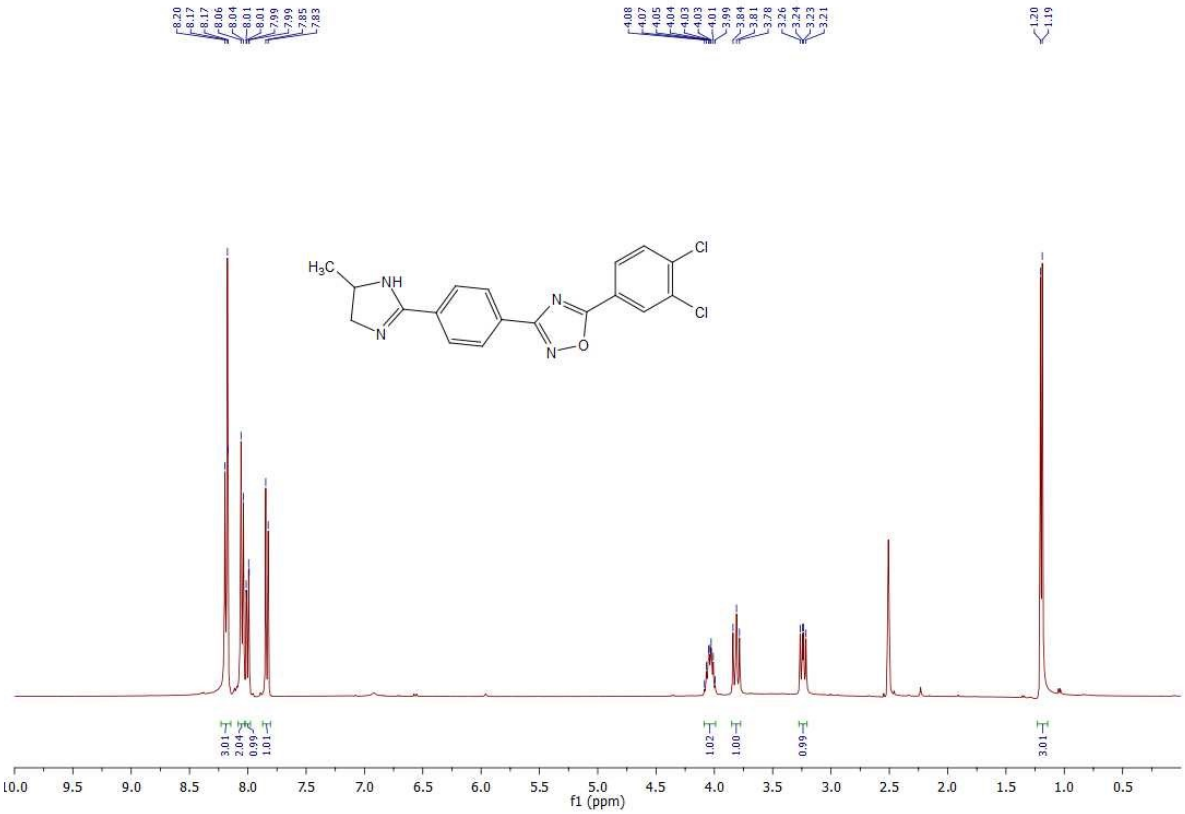

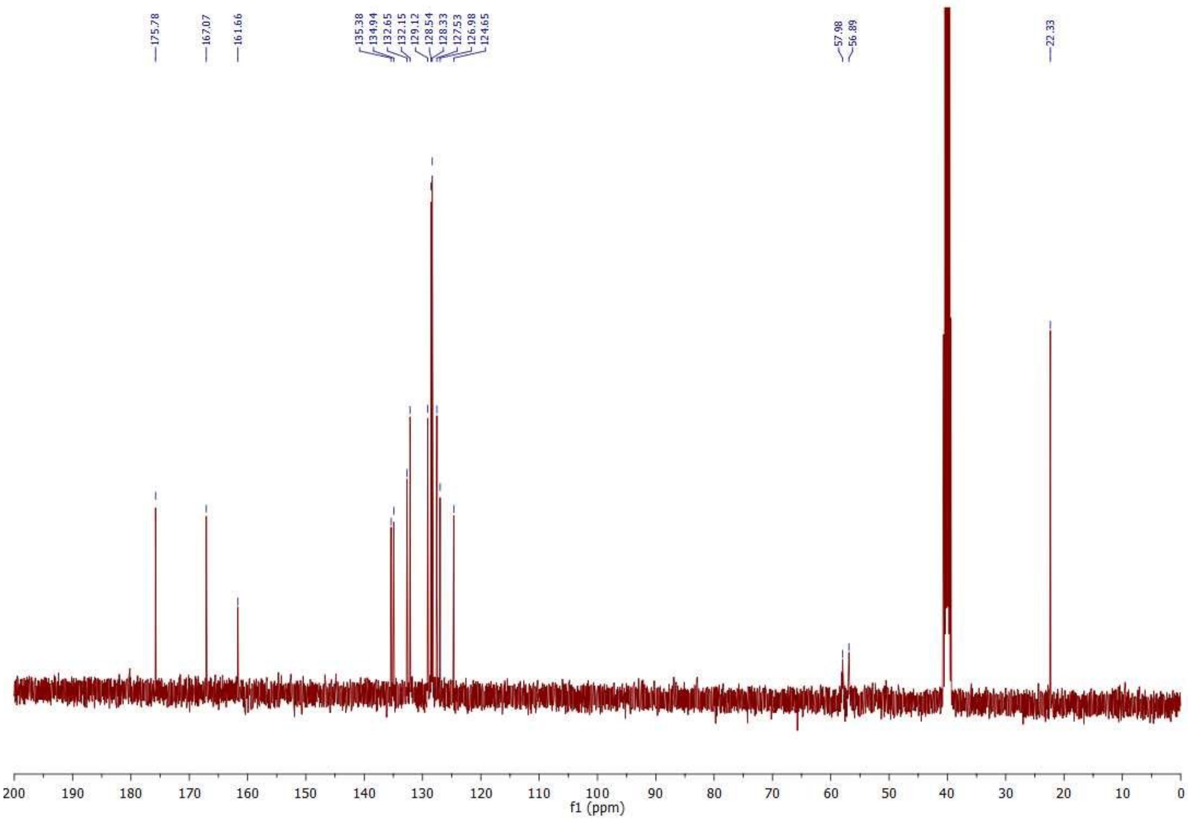

^{1}H and ^{13}C NMR spectra of 5-(3-chlorophenyl)-3-(3-(4,5-dihydro-1*H*-imidazol-2-yl)phenyl)-1,2,4-oxadiazole (**3e**)

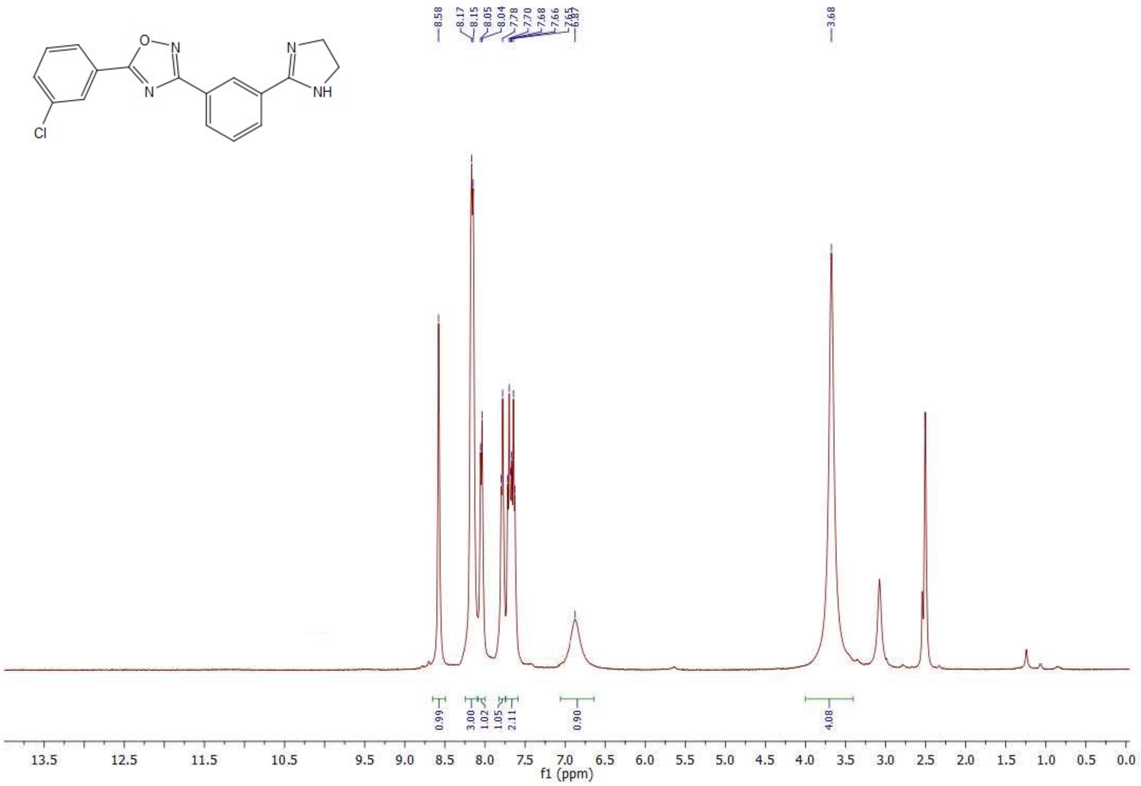

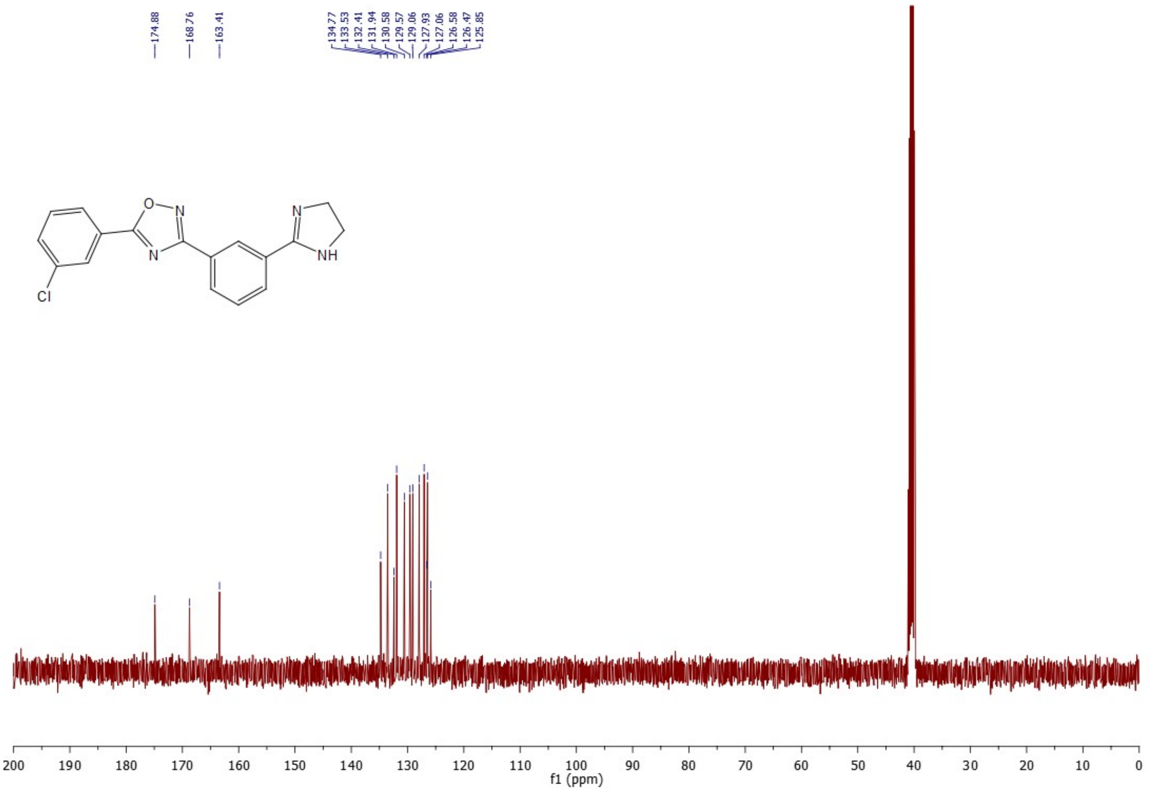

^{1}H and ^{13}C NMR spectra of 3-(3-(4,5-dihydro-1*H*-imidazol-2-yl)phenyl)-5-(thiophen-2-yl)-1,2,4-oxadiazole (**3f**)

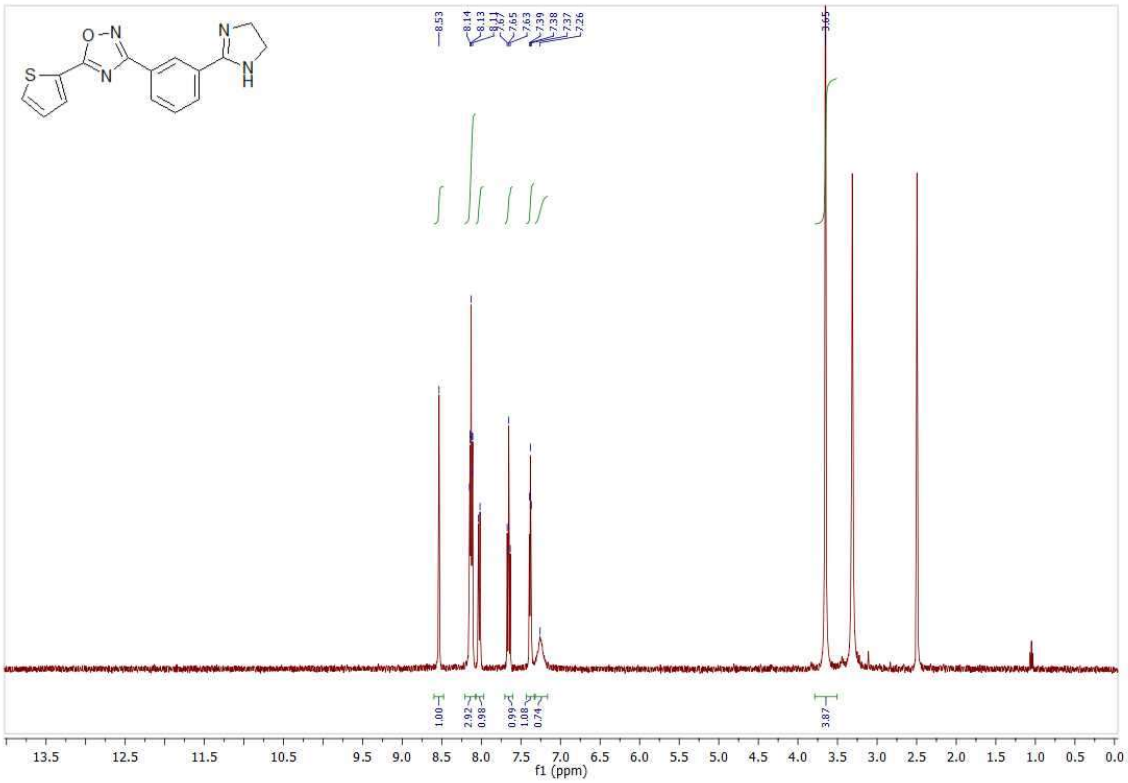

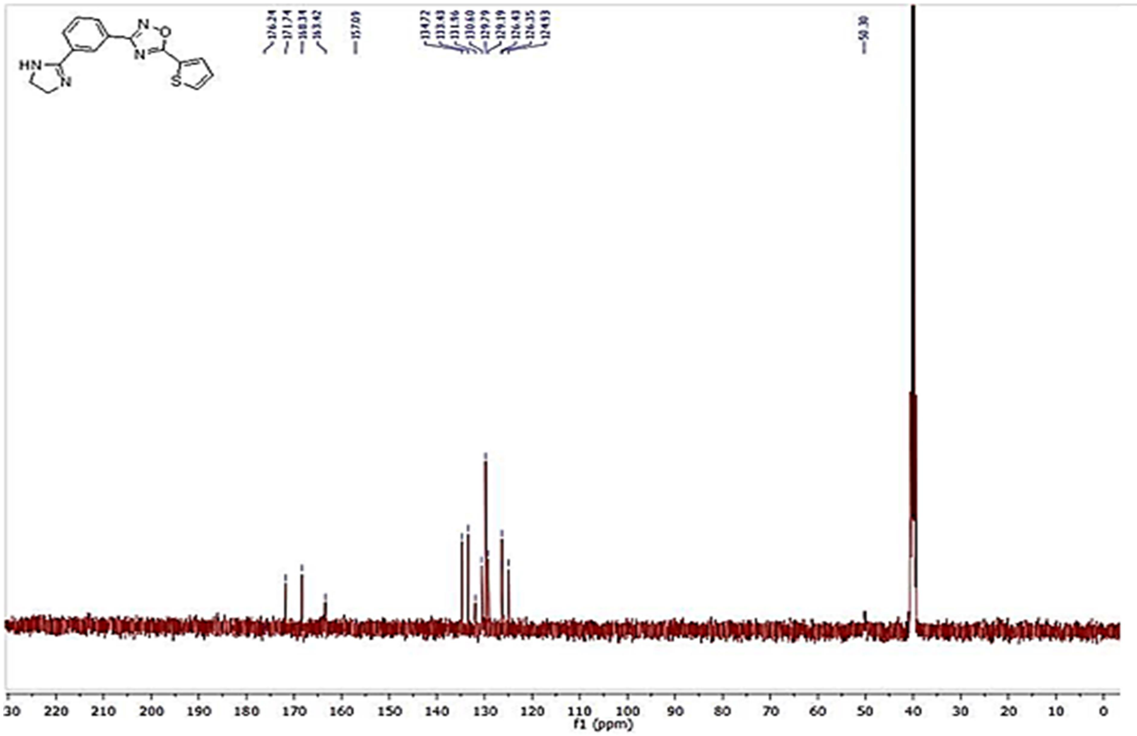

^{1}H and ^{13}C NMR spectra 2-((3-(3-(4, 5-dihydro-1*H*-imidazol-2-yl)phenyl)-1,2,4-oxadiazol-5-yl)methyl)-4-methylphthalazin-1(2*H*)-one (**3g**)

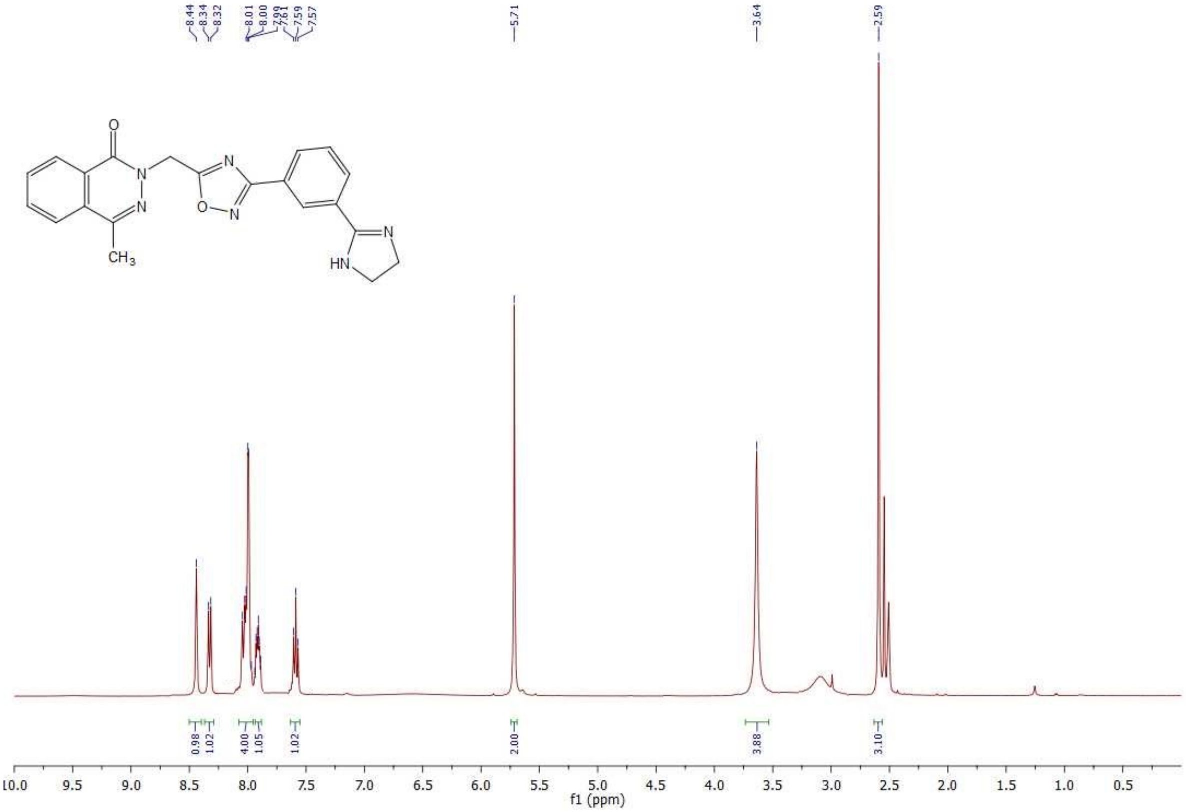

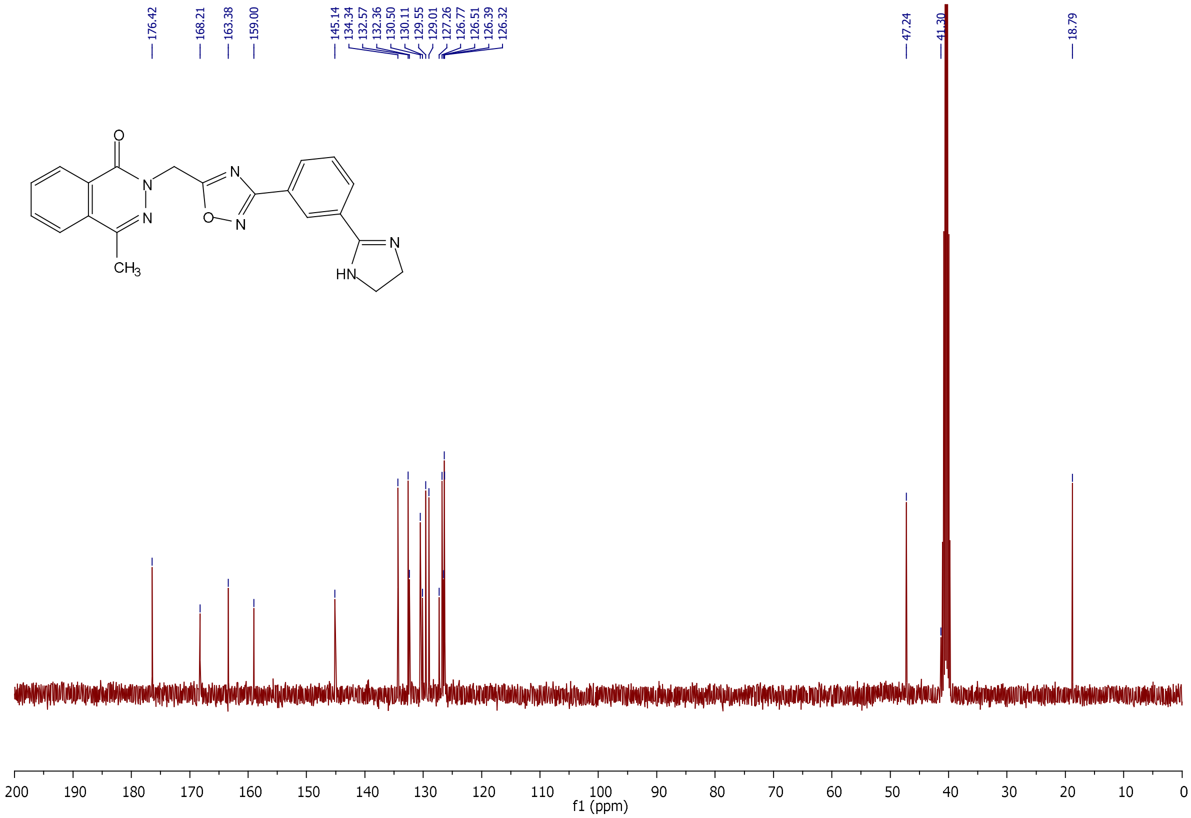

^{1}H and ^{13}C NMR spectra of 3-(4-(4,5-Dihydro-1H-imidazol-2-yl)phenyl)-5-(4-ethylphenyl)-1,2,4-oxadiazole (**3h**)

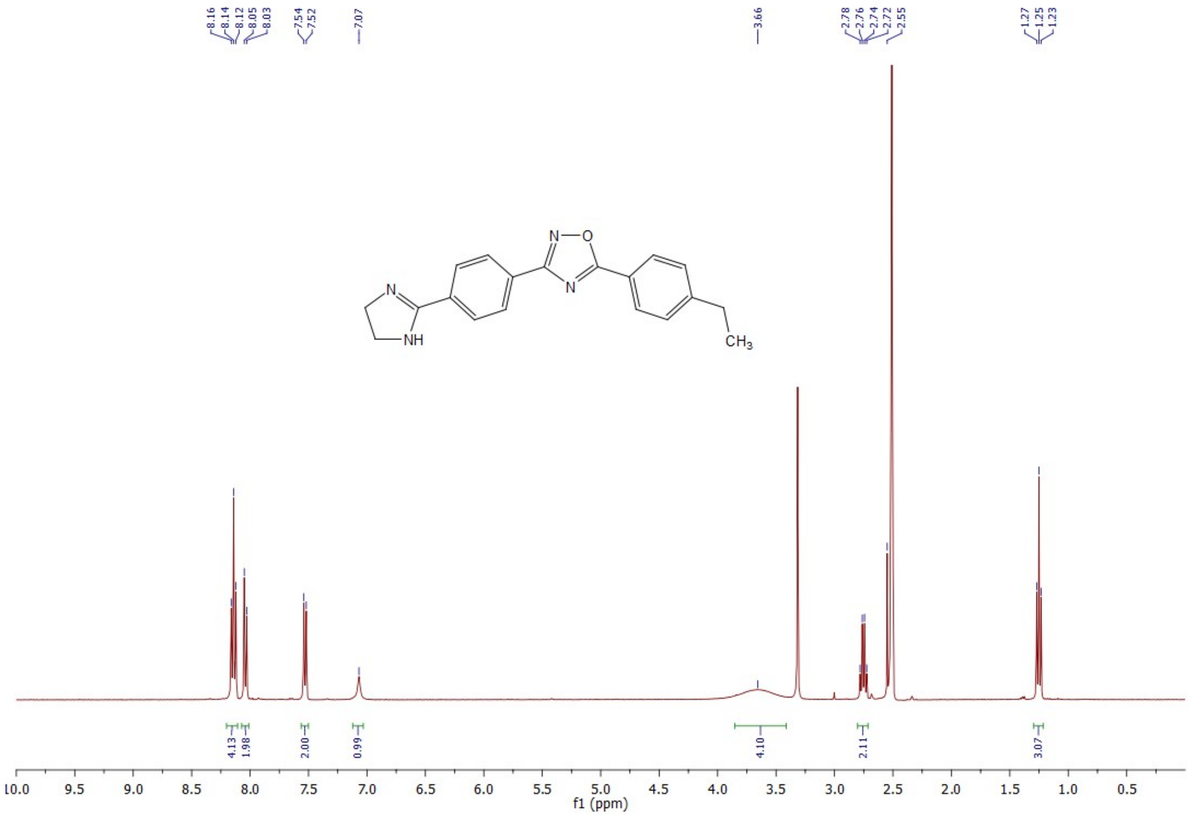

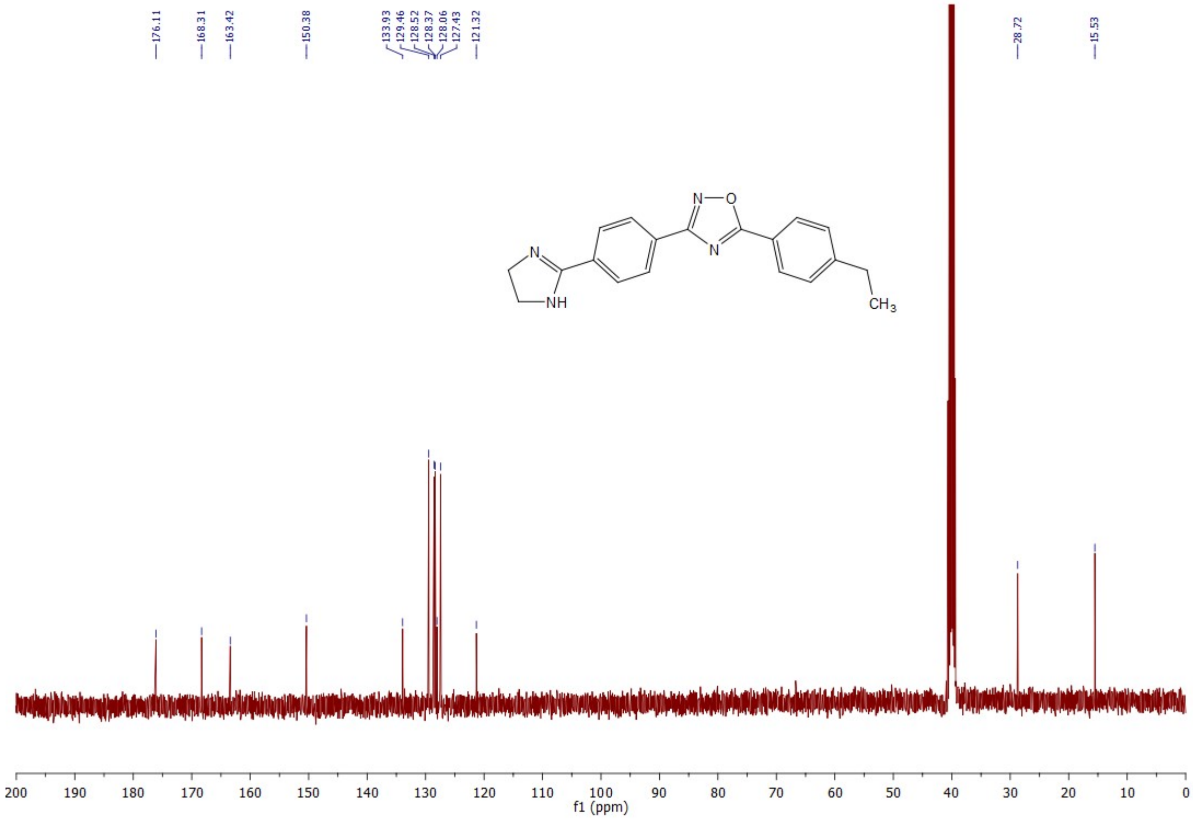

^{1}H and ^{13}C NMR spectra of 2-((5-(4-(4,5-dihydro-1*H*-imidazol-2-yl)phenyl)-1,2,4-oxadiazol-3-yl)methyl)-6-phenylpyridazin-3(2*H*)-one (**5a**)

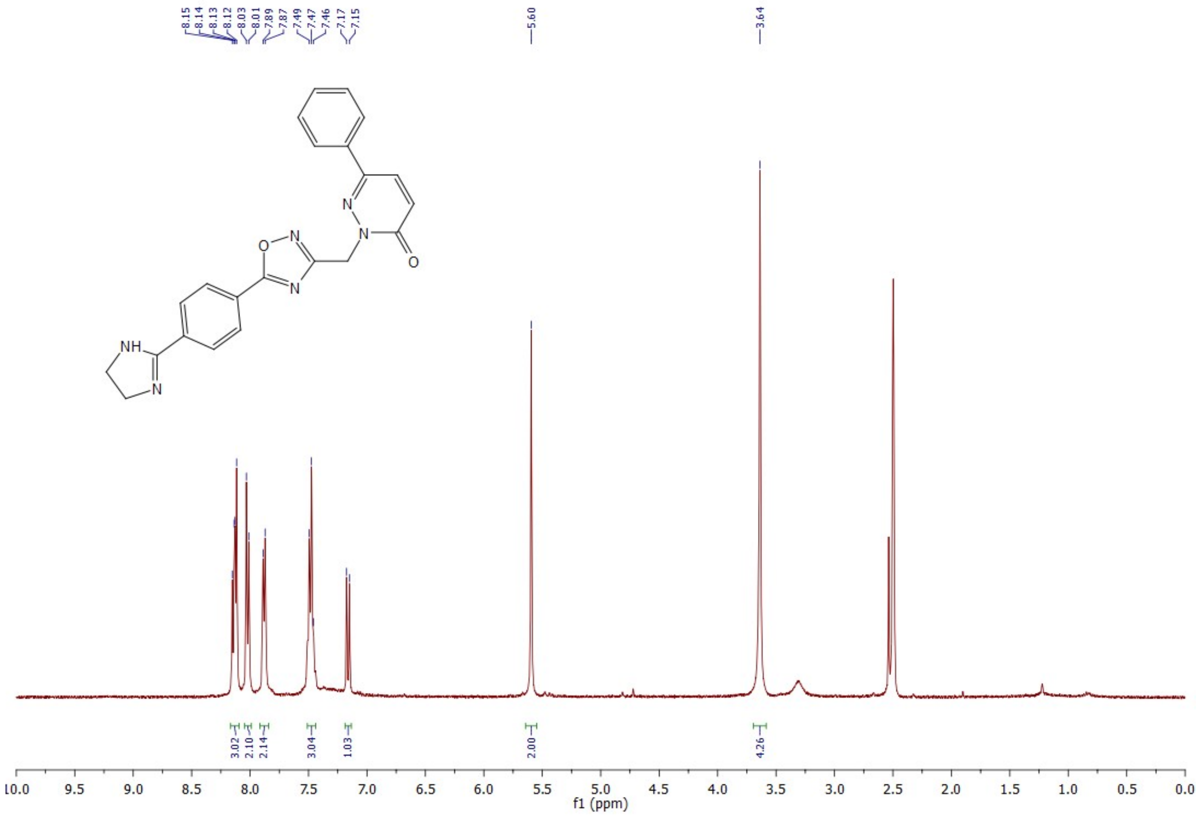

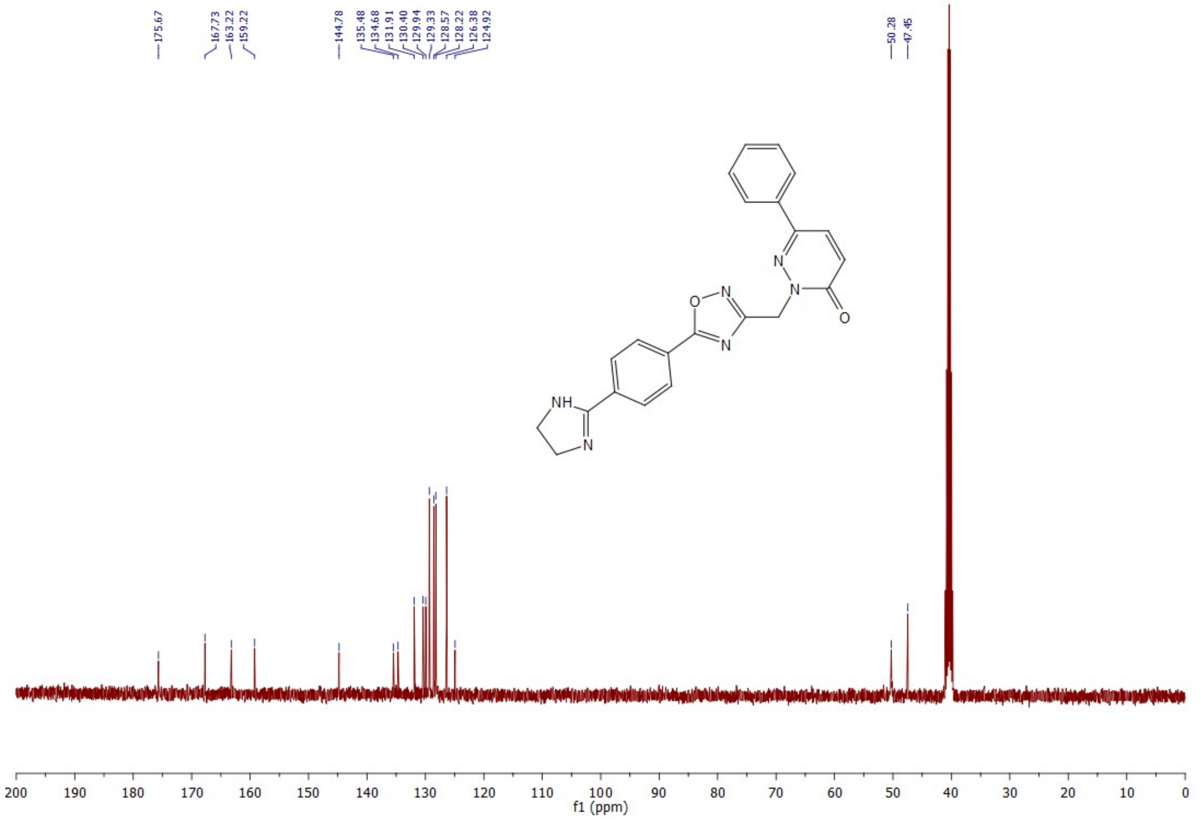

^{1}H and ^{13}C NMR spectra of 5-(4-(4,5-dihydro-1*H*-imidazol-2-yl)phenyl)-3-isopropyl-1,2,4-oxadiazole (**5b**)

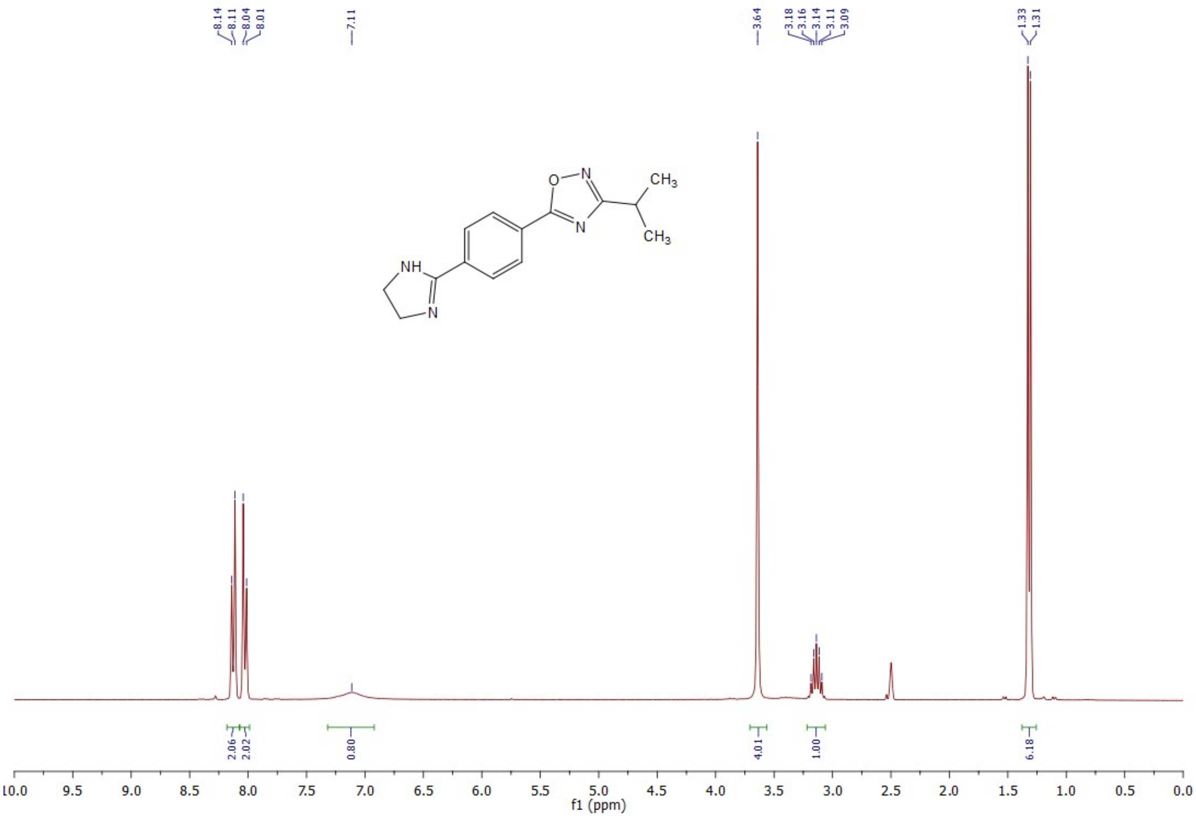

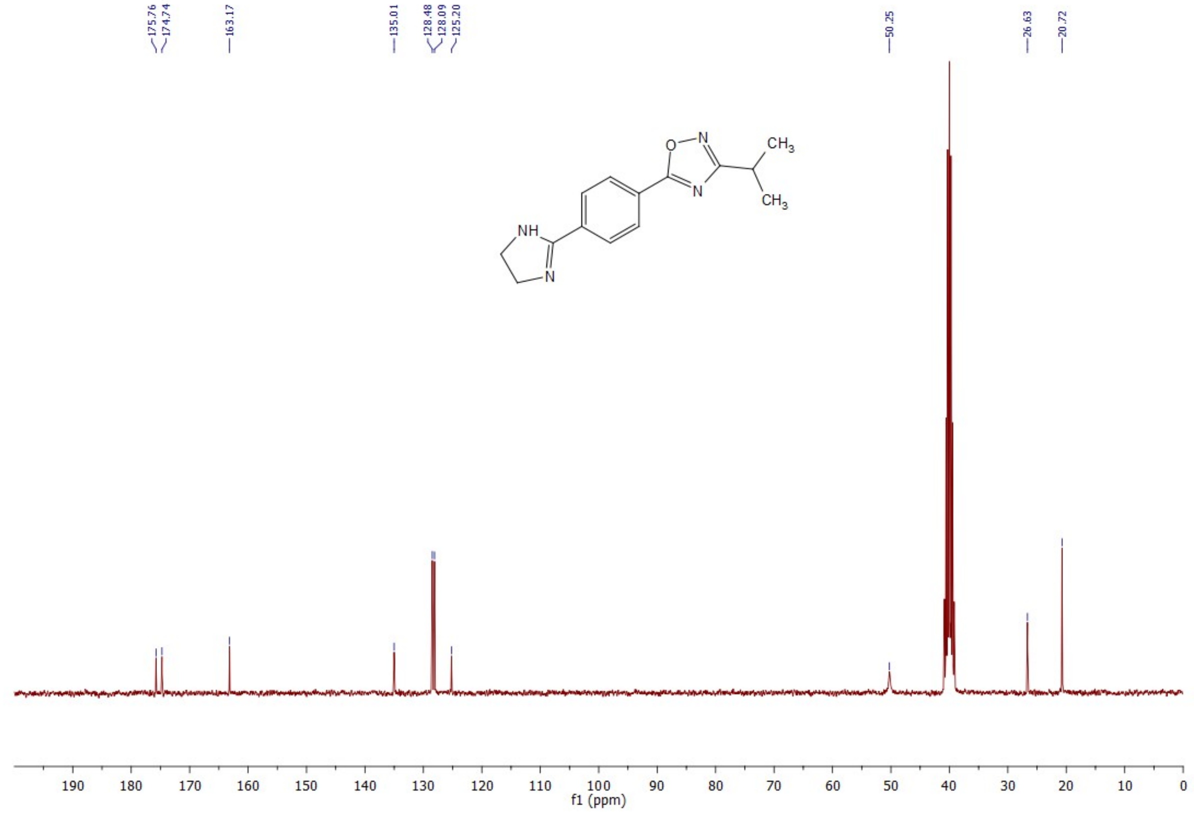

^{1}H and ^{13}C NMR spectra of 5-(4-(4,5-dihydro-1*H*-imidazol-2-yl)phenyl)-3-(phenoxymethyl)-1,2,4-oxadiazole (**5c**)

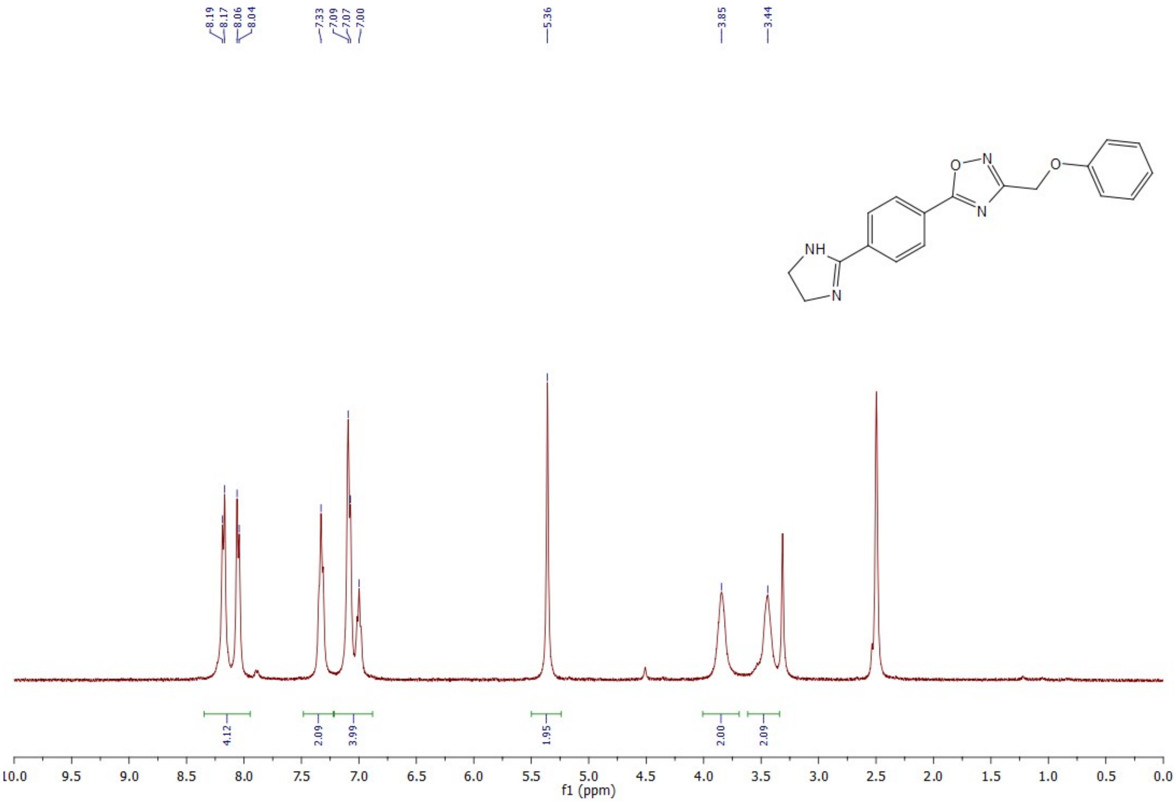

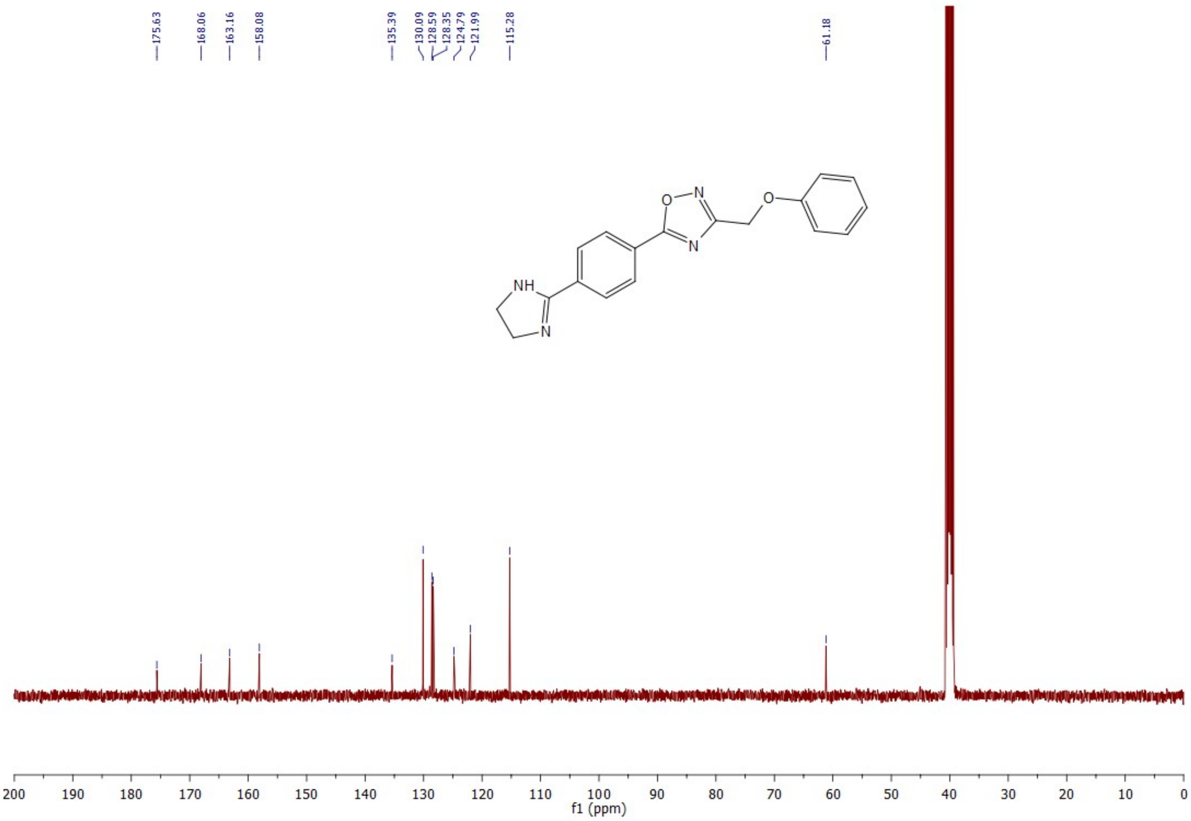

^{1}H and ^{13}C NMR spectra of 3-(4-*tert*-butylphenyl)-5-(4-(4,5-dihydro-1*H*-imidazol-2-yl)phenyl)-1,2,4-oxadiazole (**5d**)

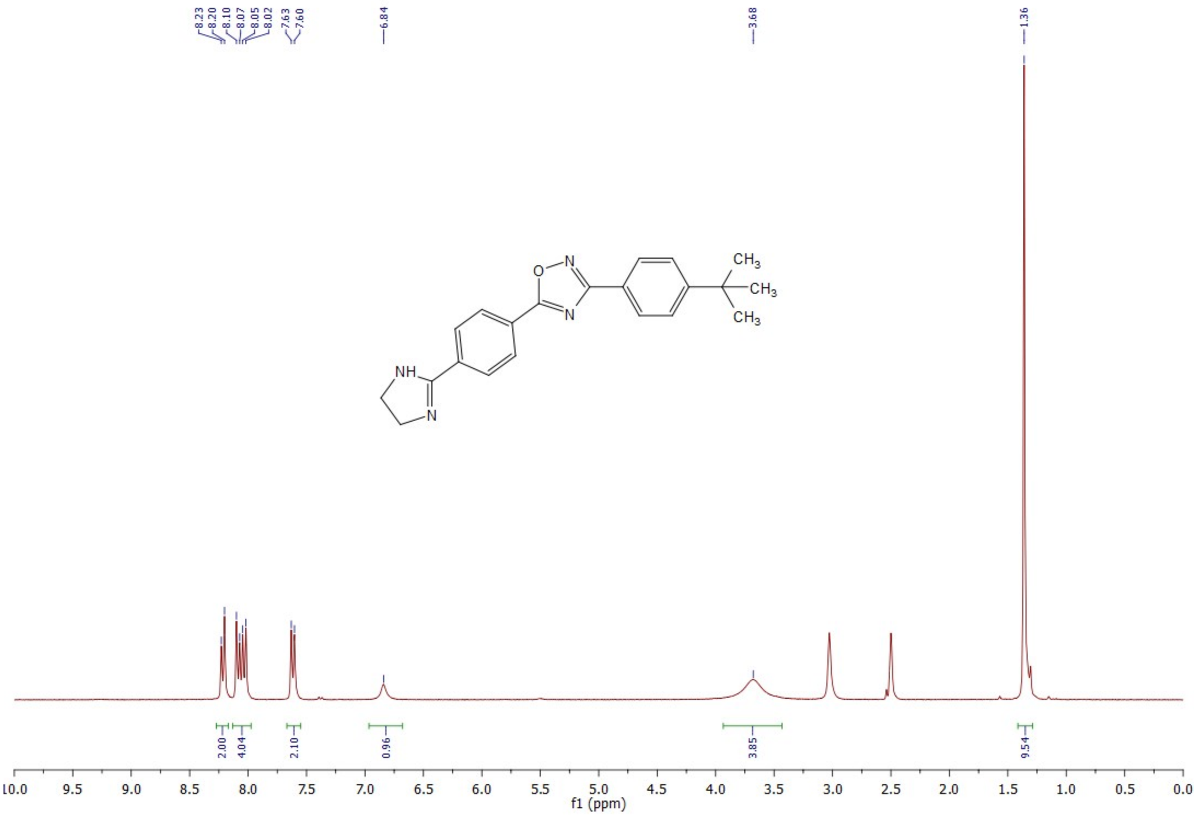

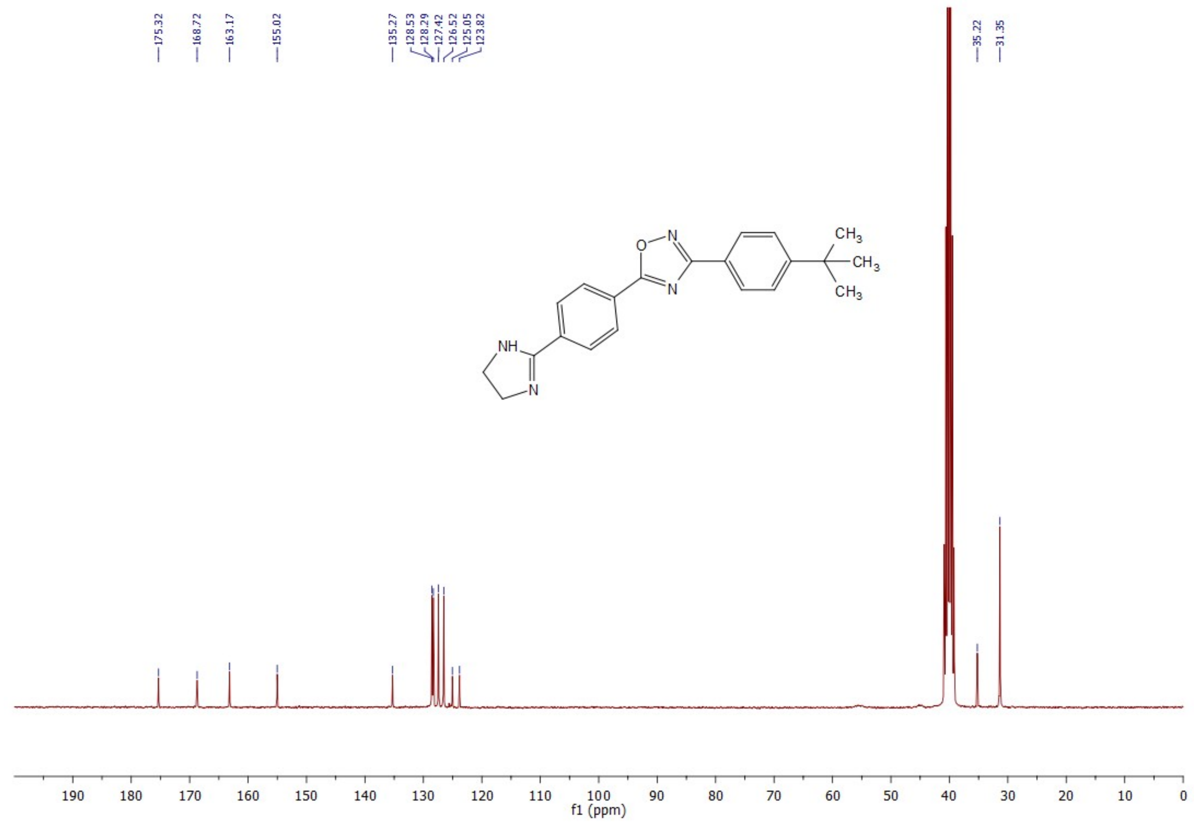

^{1}H and ^{13}C NMR spectra of 5-(4-(4,5-dihydro-1*H*-imidazol-2-yl)phenyl)-3-(4-phenoxyphenyl)-1,2,4-oxadiazole (**5e**)

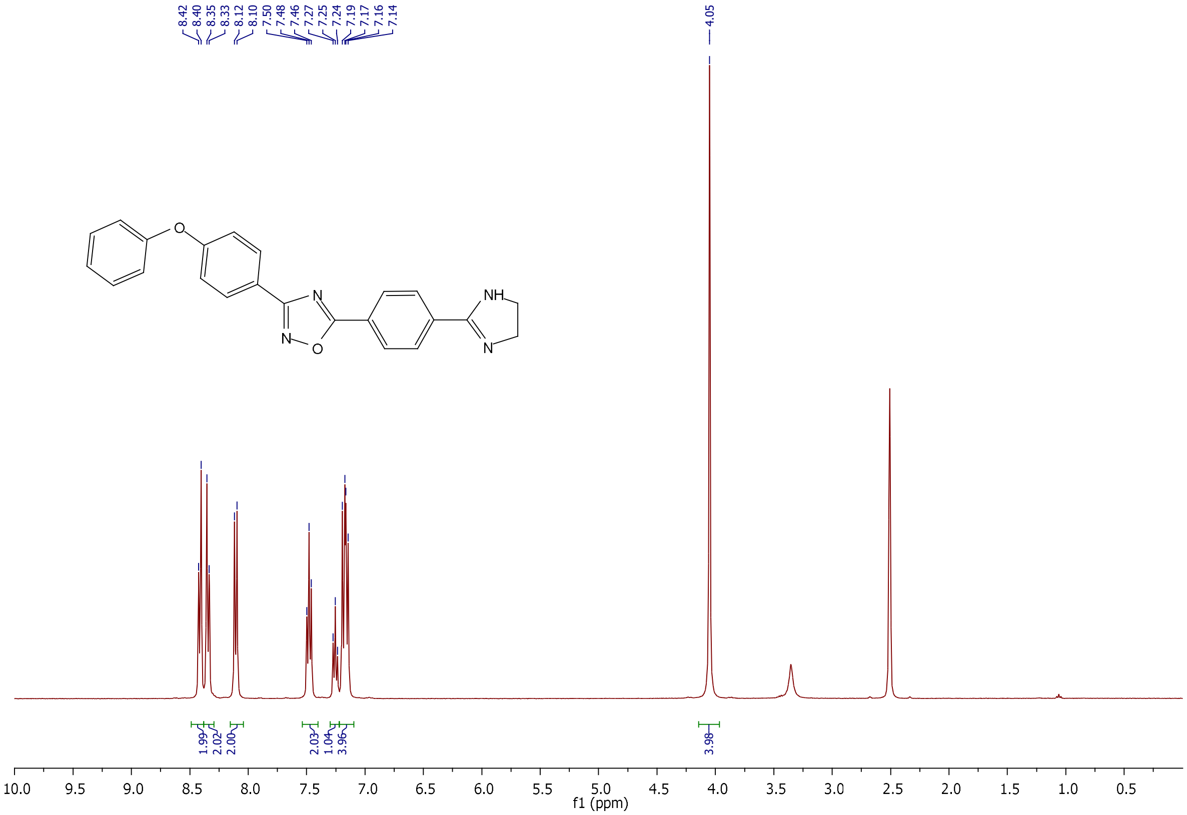

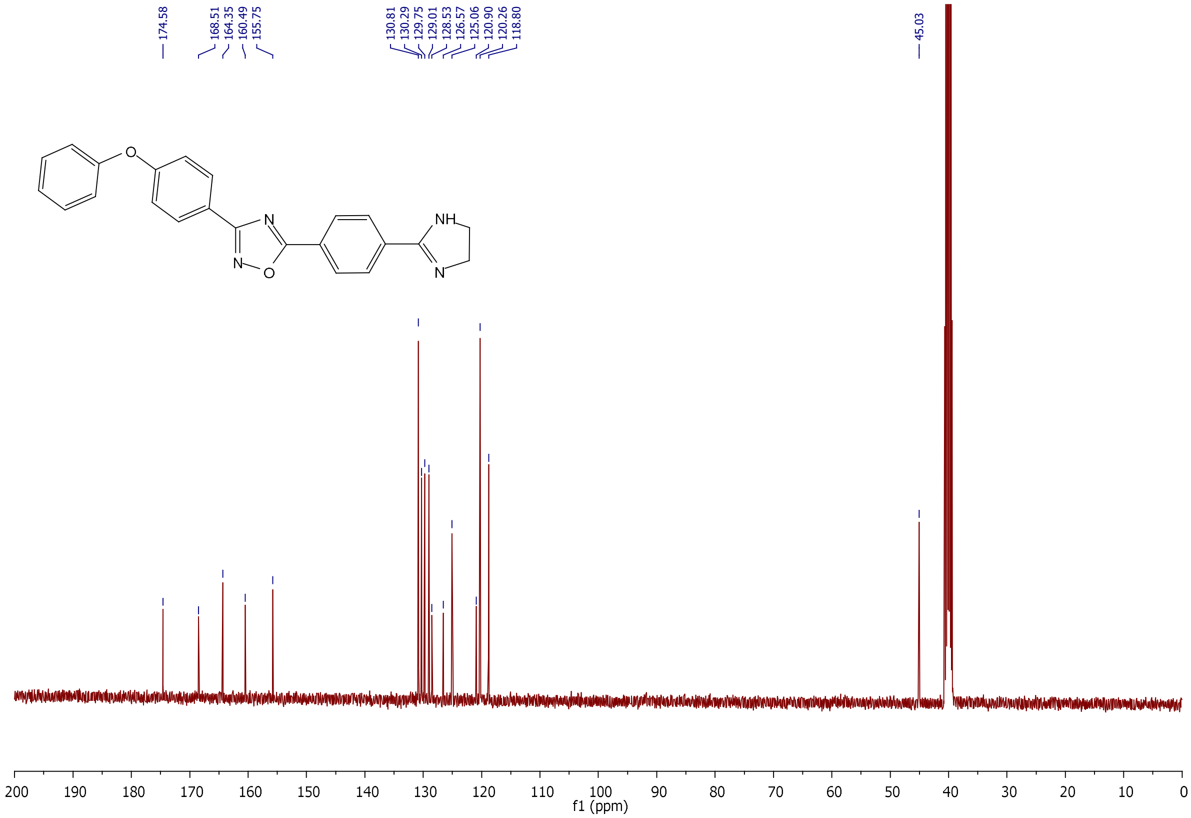

^{1}H and ^{13}C NMR spectra of 5-(4-(4,5-dihydro-1*H*-imidazol-2-yl)phenyl)-3-(thiophen-2-yl)-1,2,4-oxadiazole (**5f**)

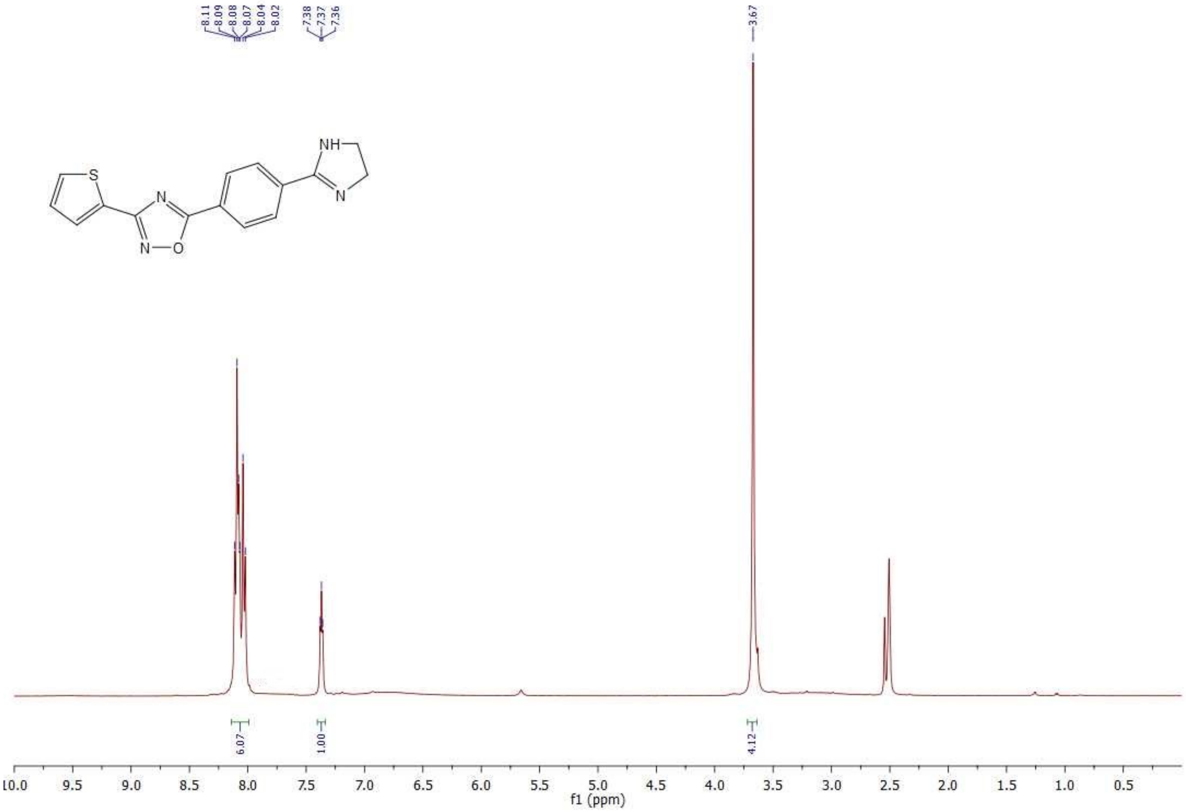

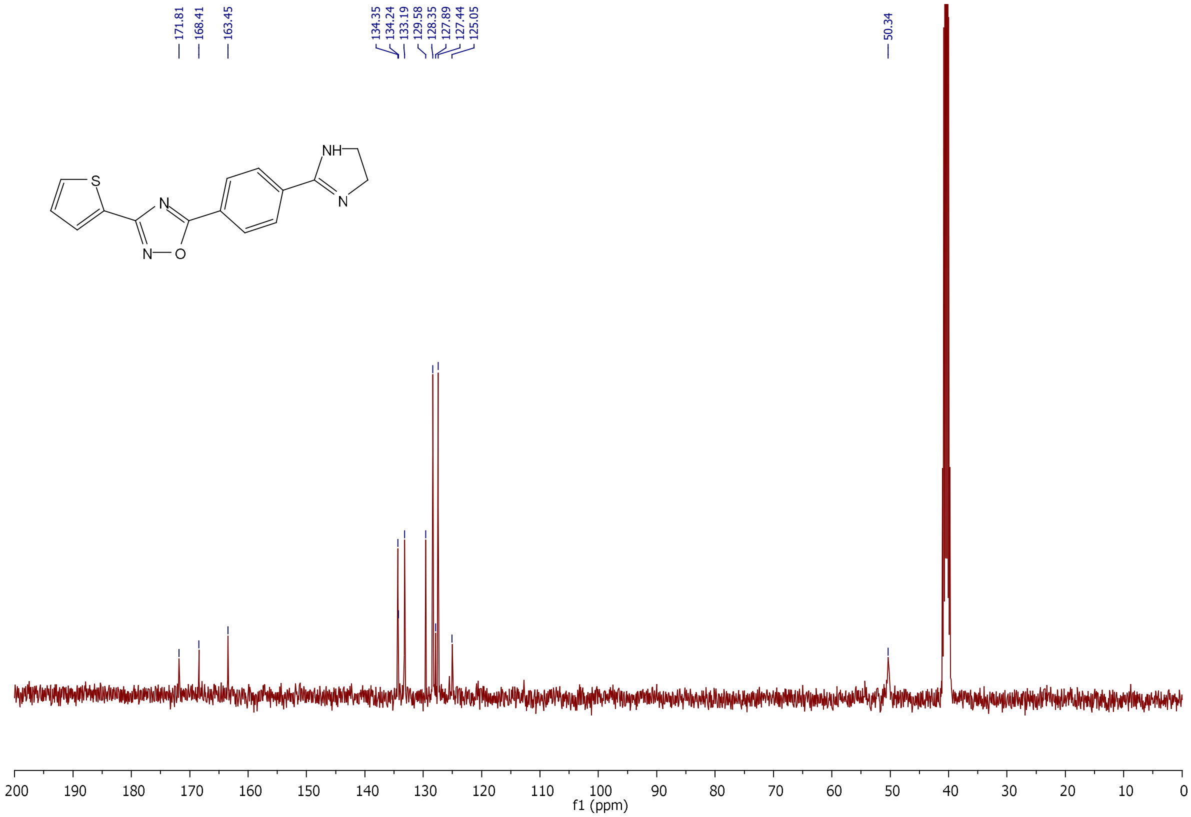

^{1}H and ^{13}C NMR spectra of 3-(3-chlorophenyl)-5-(4-(4,5-dihydro-1*H*-imidazol-2-yl)phenyl)-1,2,4-oxadiazole (**5g**)

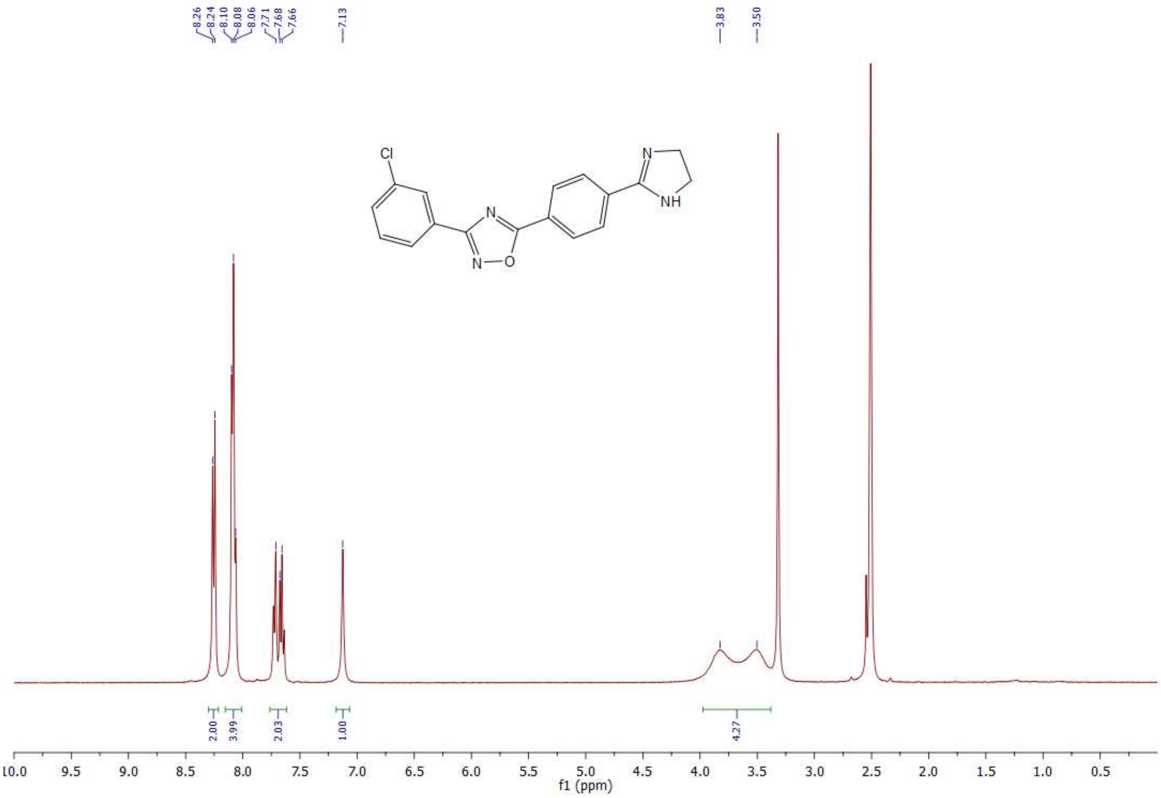

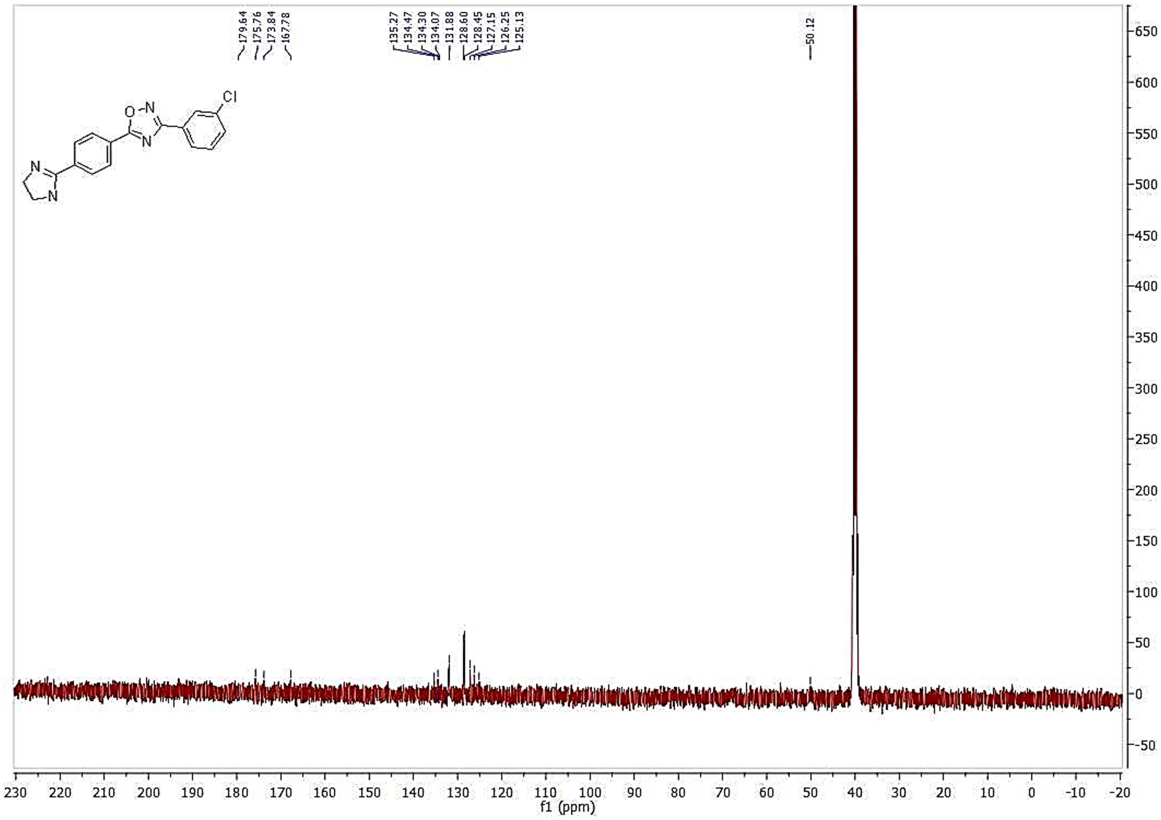

^{1}H and ^{13}C NMR spectra of 3-(2-chlorophenyl)-5-(4-(4,5-dihydro-1*H*-imidazol-2-yl)phenyl)-1,2,4-oxadiazole (**5h**)

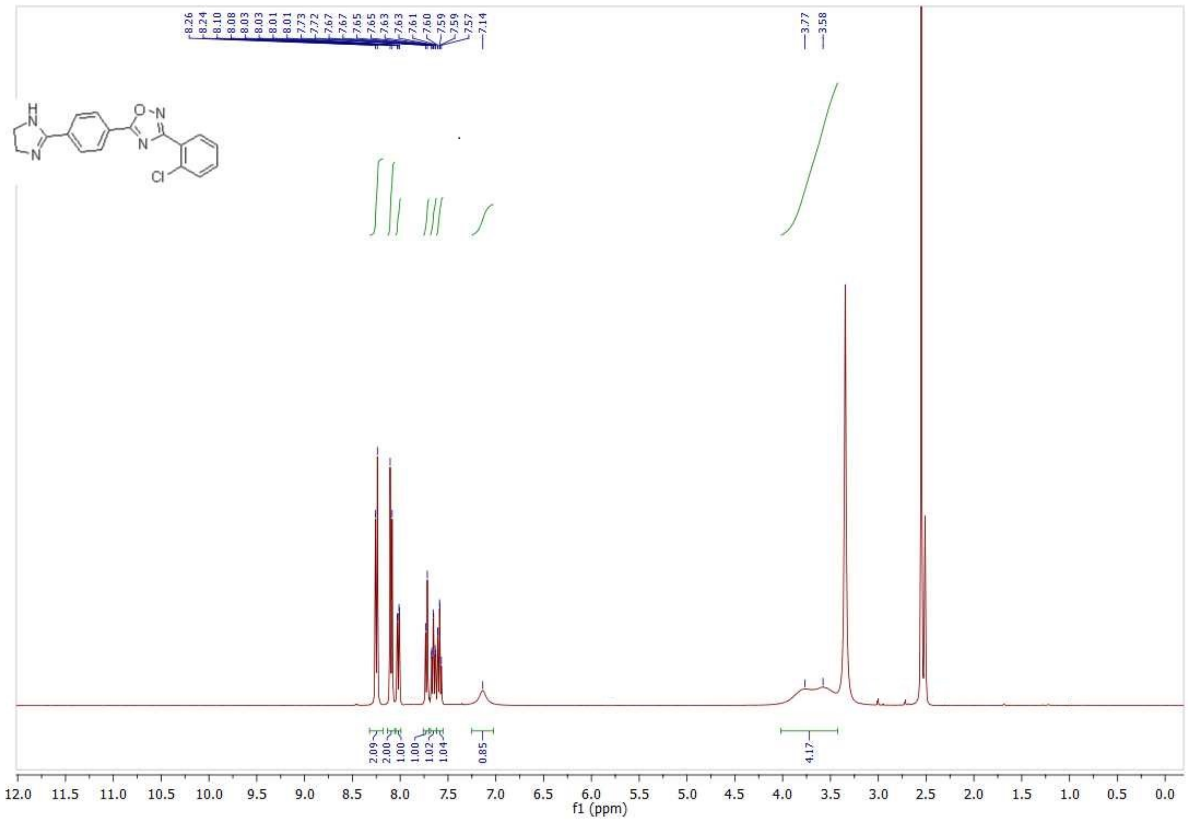

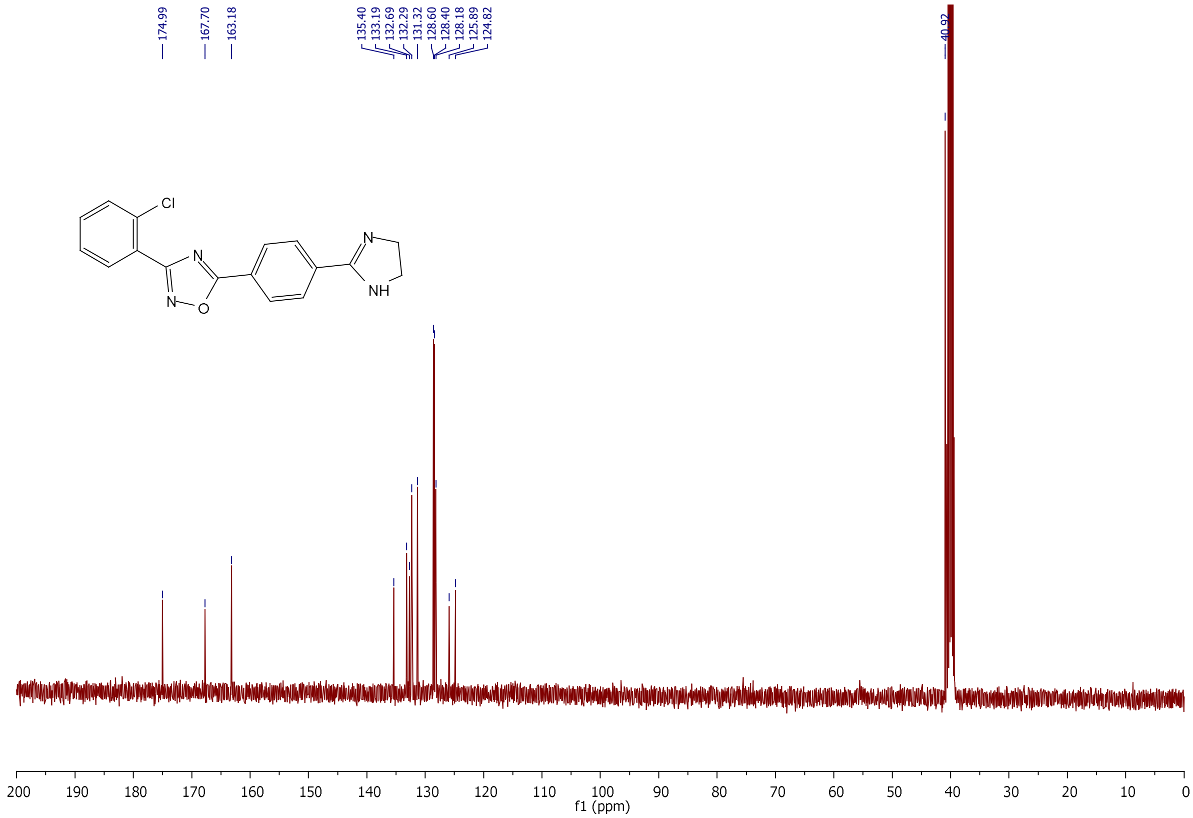

^{1}H and ^{13}C NMR spectra of 2-((5-(4-(4,5-dihydro-1*H*-imidazol-2-yl)phenyl)-1,2,4-oxadiazol-3-yl)methyl)phthalazin-1(2*H*)-one (**5i**)

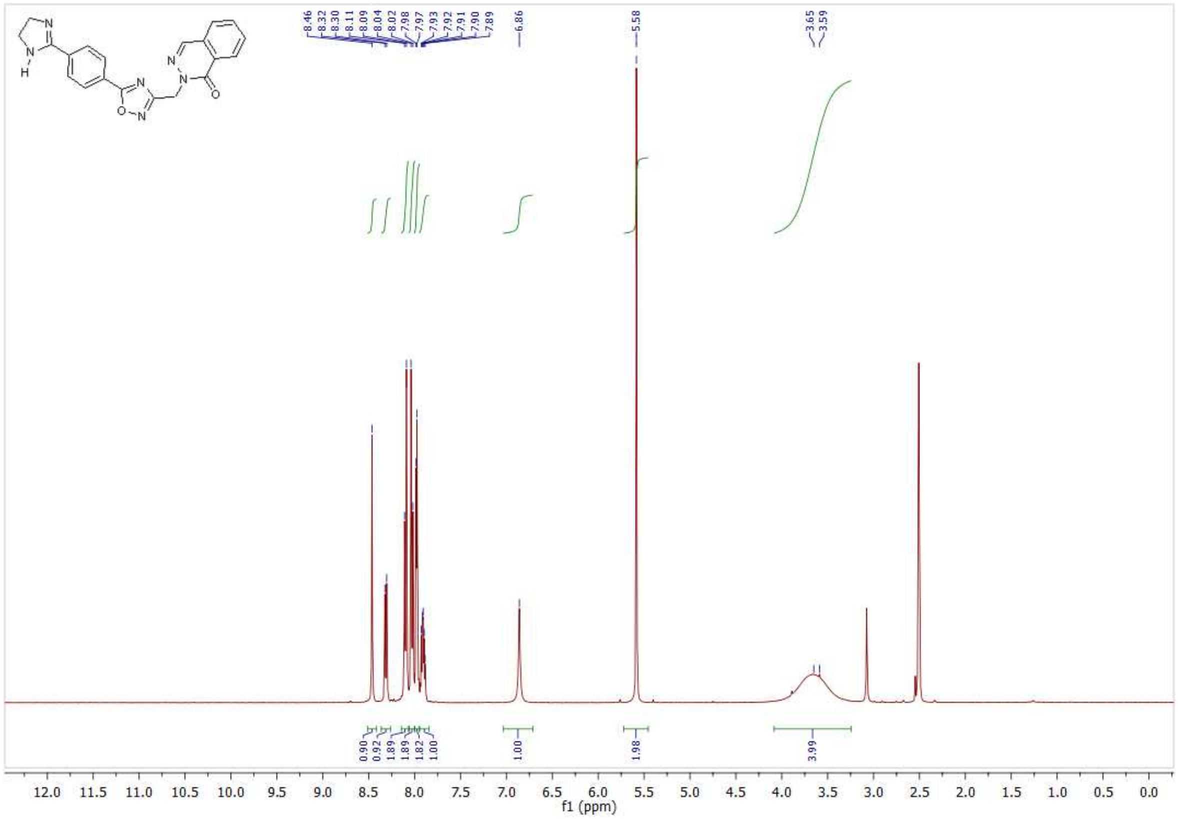

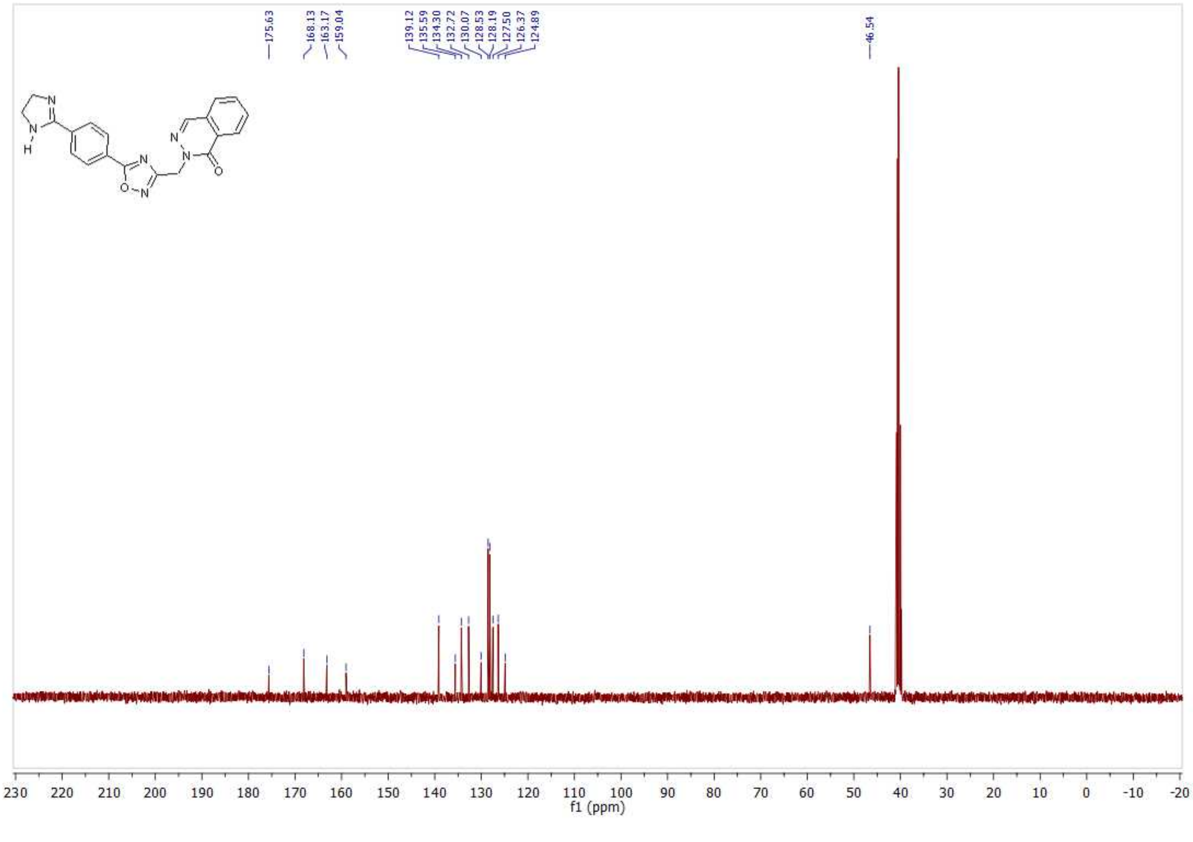

^{1}H and ^{13}C NMR spectra 5-(4-(4,5-dihydro-1*H*-imidazol-2-yl)phenyl)-3-(3,4-dimethoxyphenyl)-1,2,4-oxadiazole (**5j**)

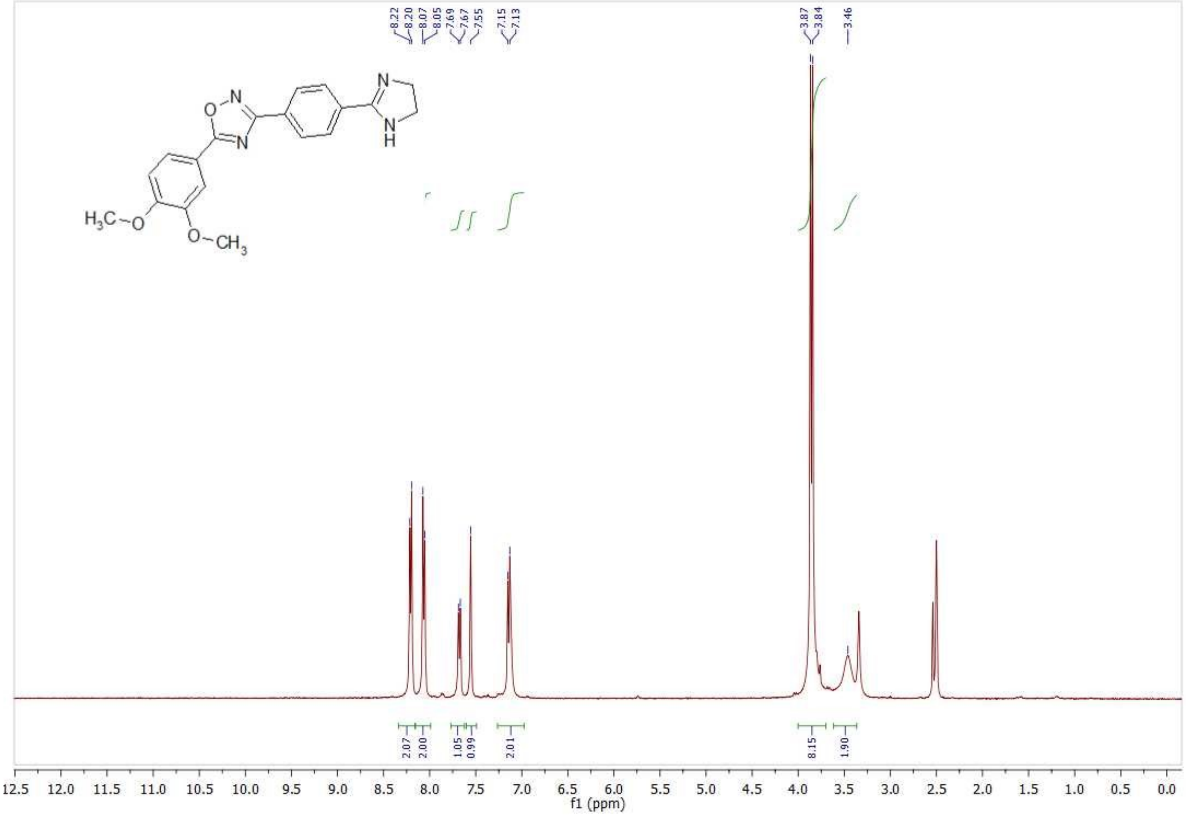

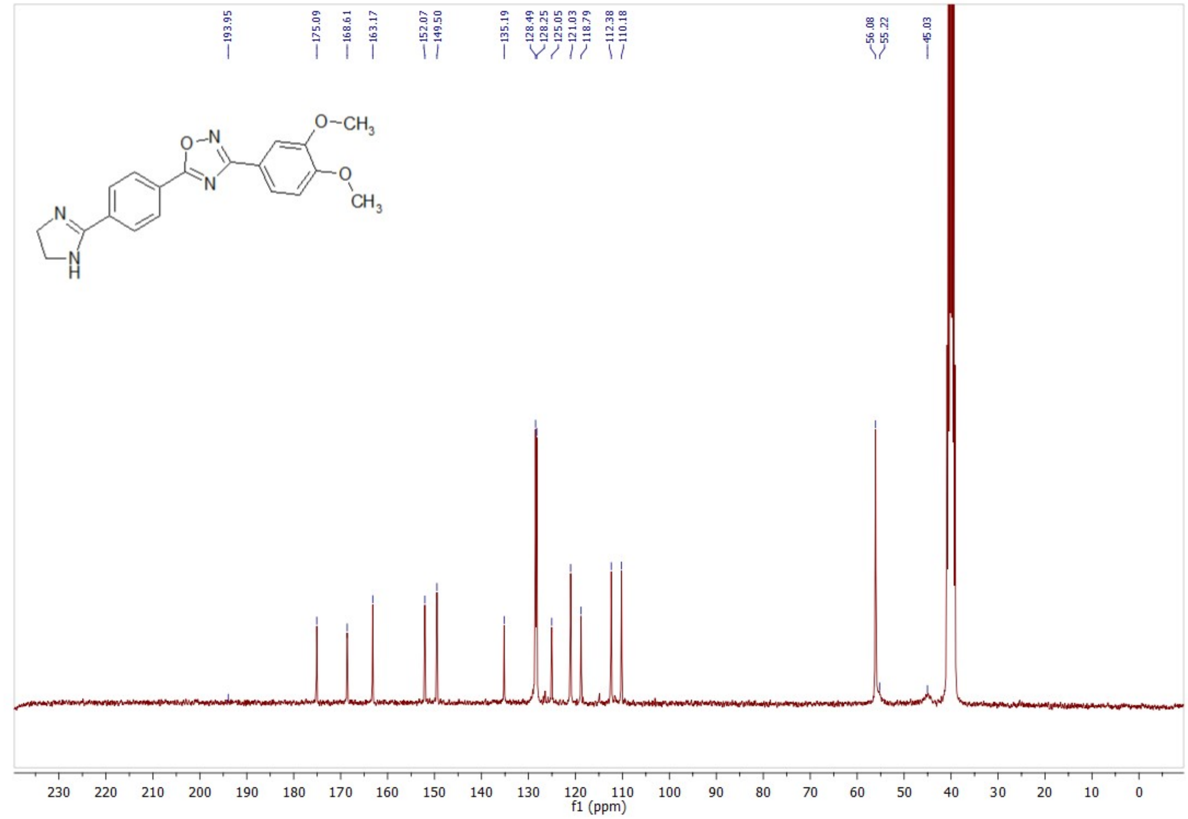

^{1}H and ^{13}C NMR spectra of 5-(4-(4,5-dihydro-1*H*-imidazol-2-yl)phenyl)-3-(3-methoxyphenyl)-1,2,4-oxadiazole (**5k**)

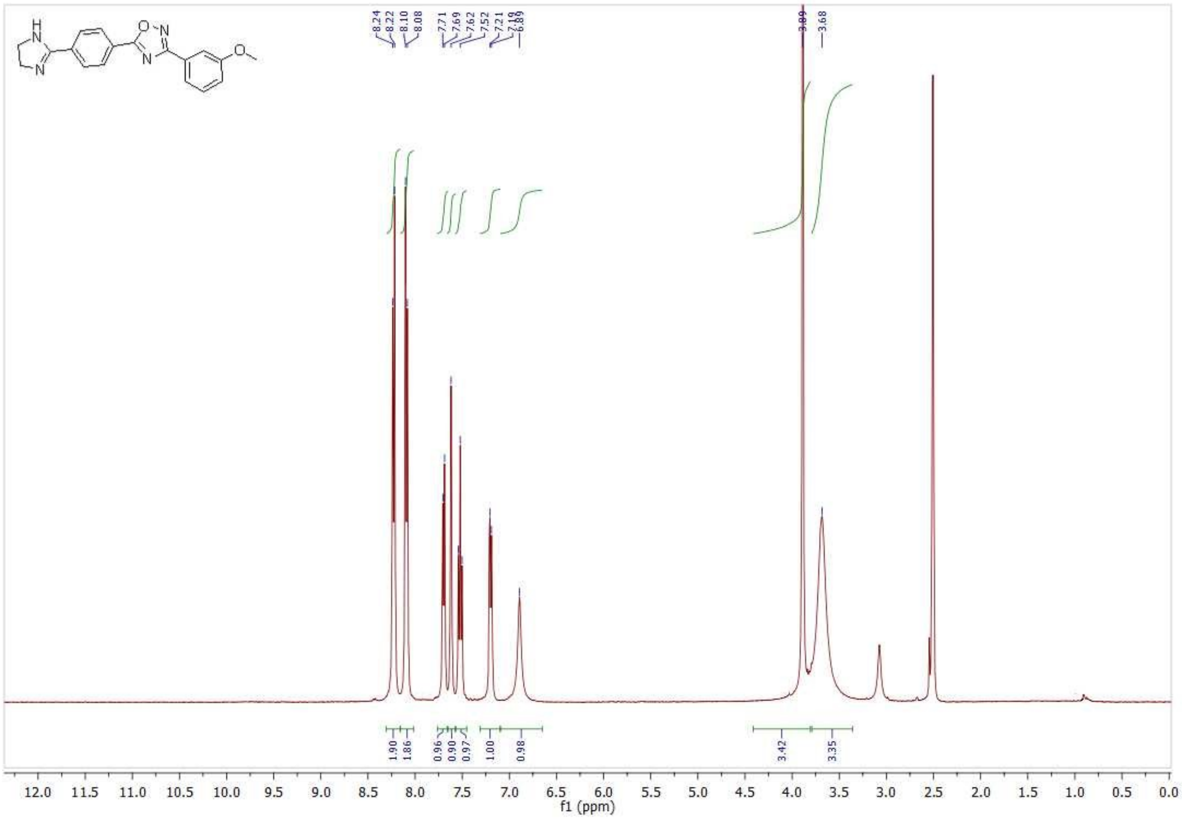

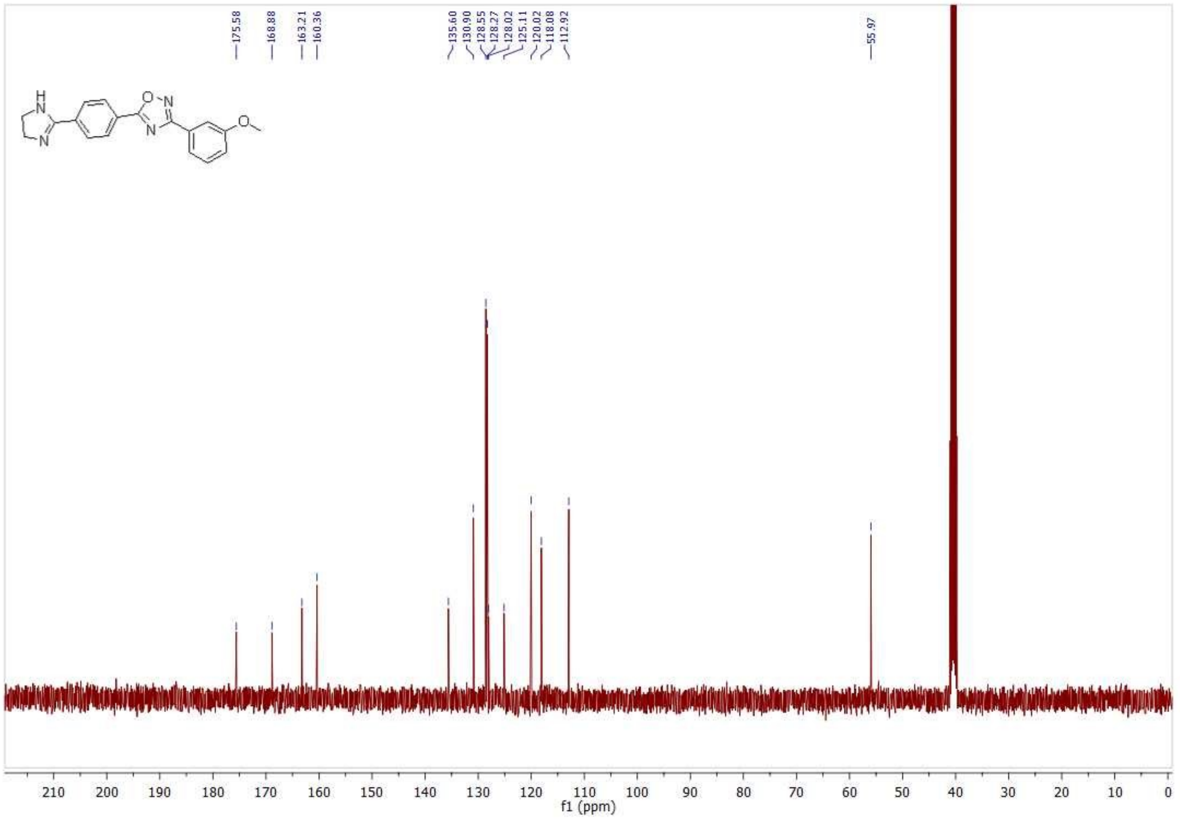

^{1}H and ^{13}C NMR spectra of 3-(4-((1*H*-1,2,4-triazol-1-yl)methyl)phenyl)-5-(4-(4,5-dihydro-1*H*-imidazol-2-yl)phenyl)-1,2,4-oxadiazole (**5l**)

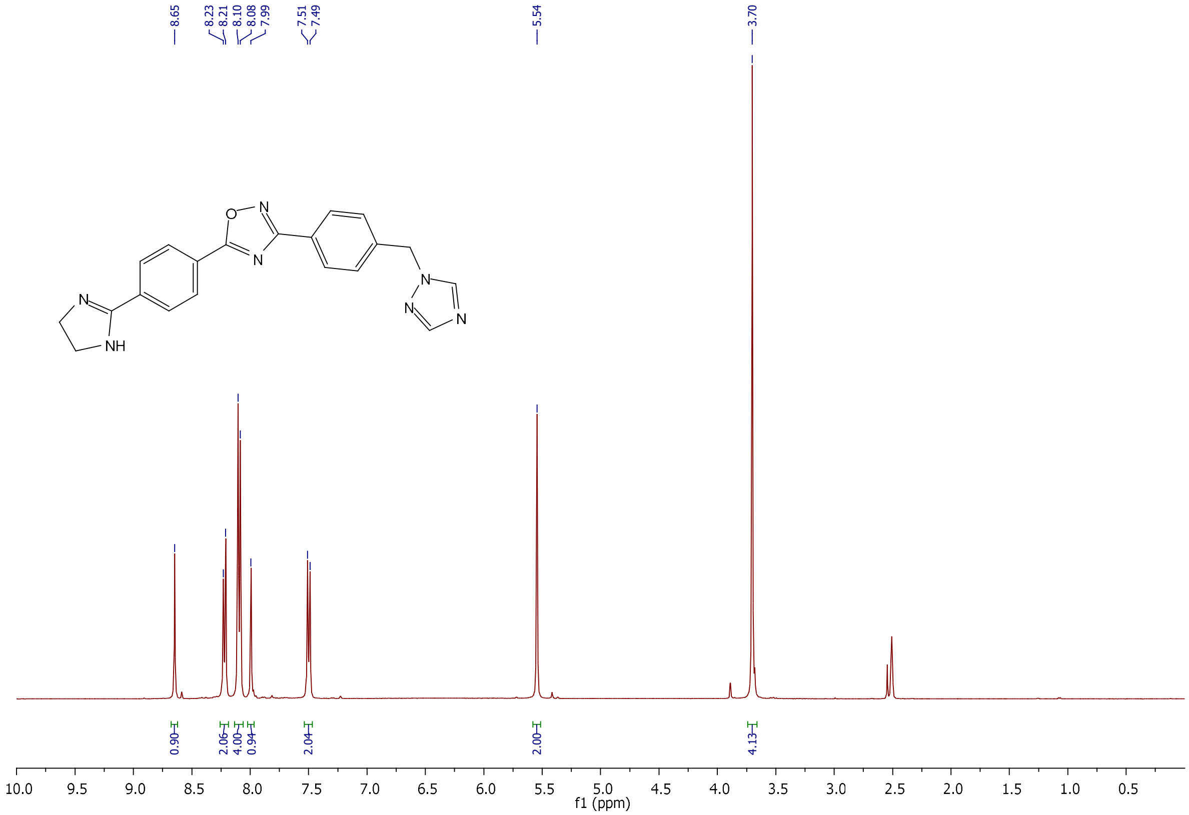

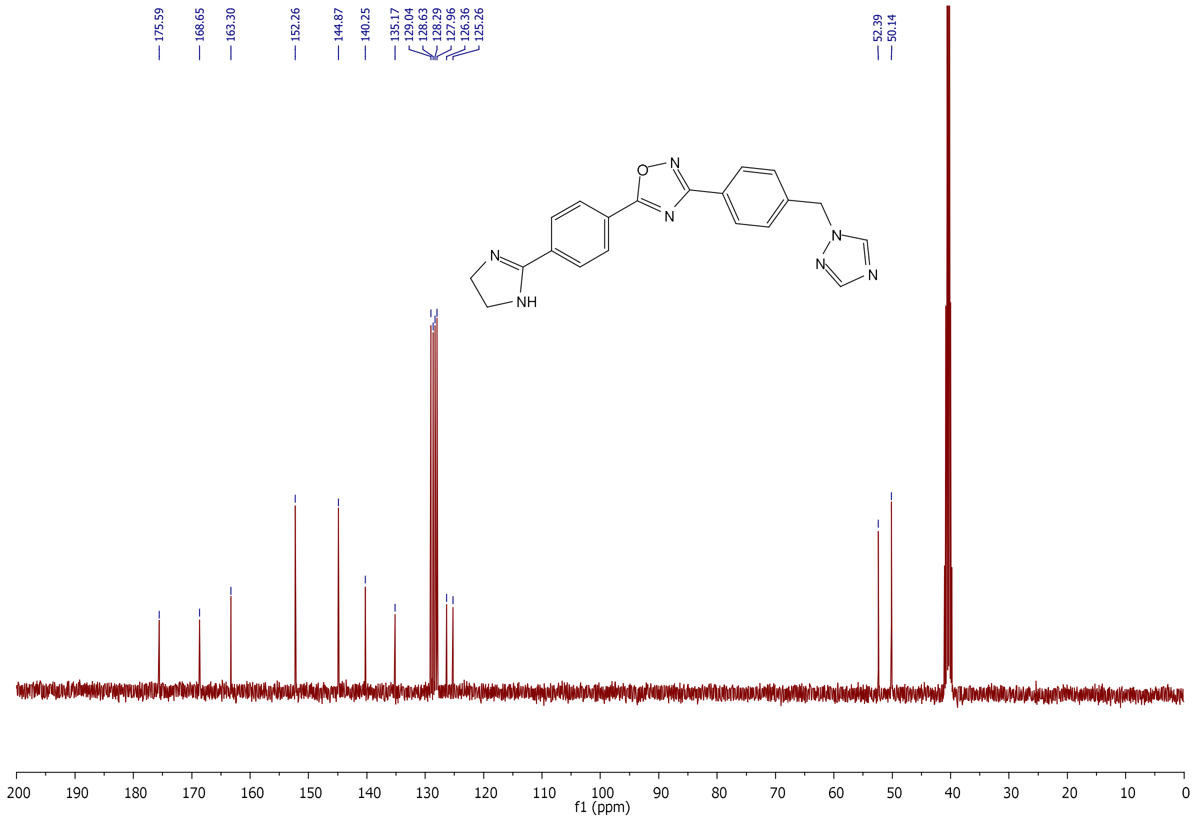

^1H and ^{13}C NMR spectra of 5-(4-(4,5-dihydro-1*H*-imidazol-2-yl)phenyl)-3-(4-methylbenzyl)-1,2,4-oxadiazole (**5m**)

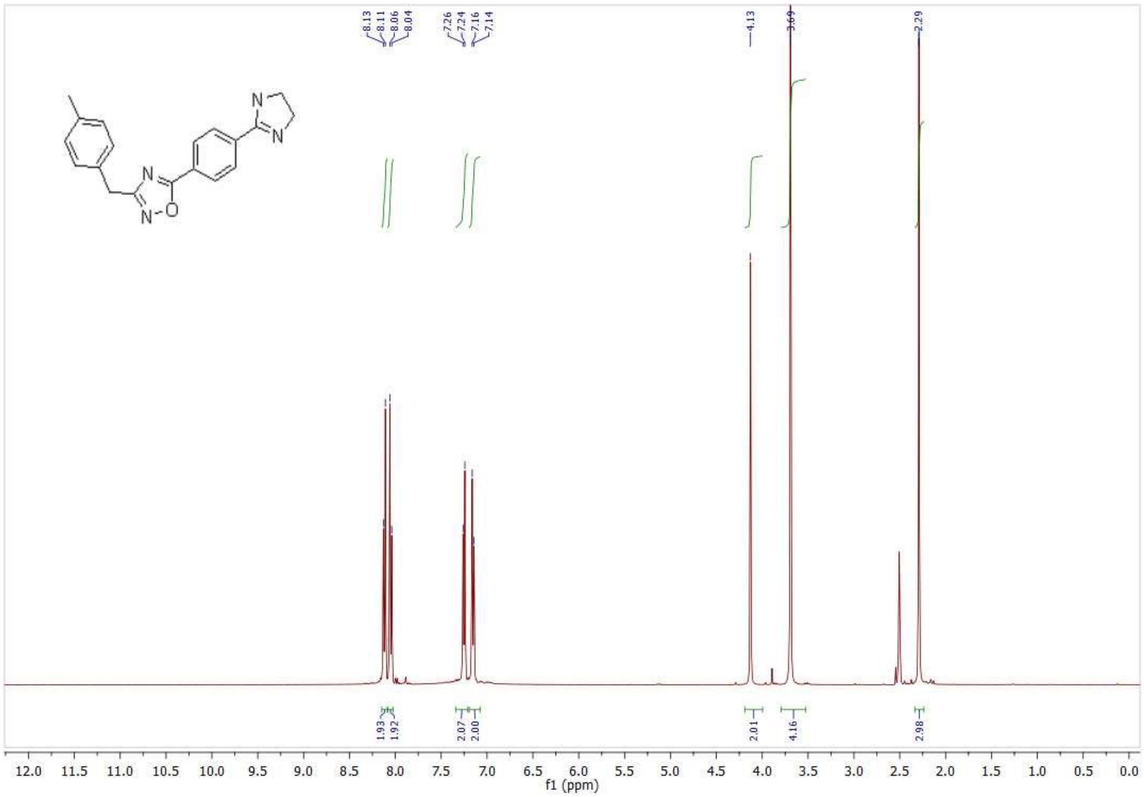

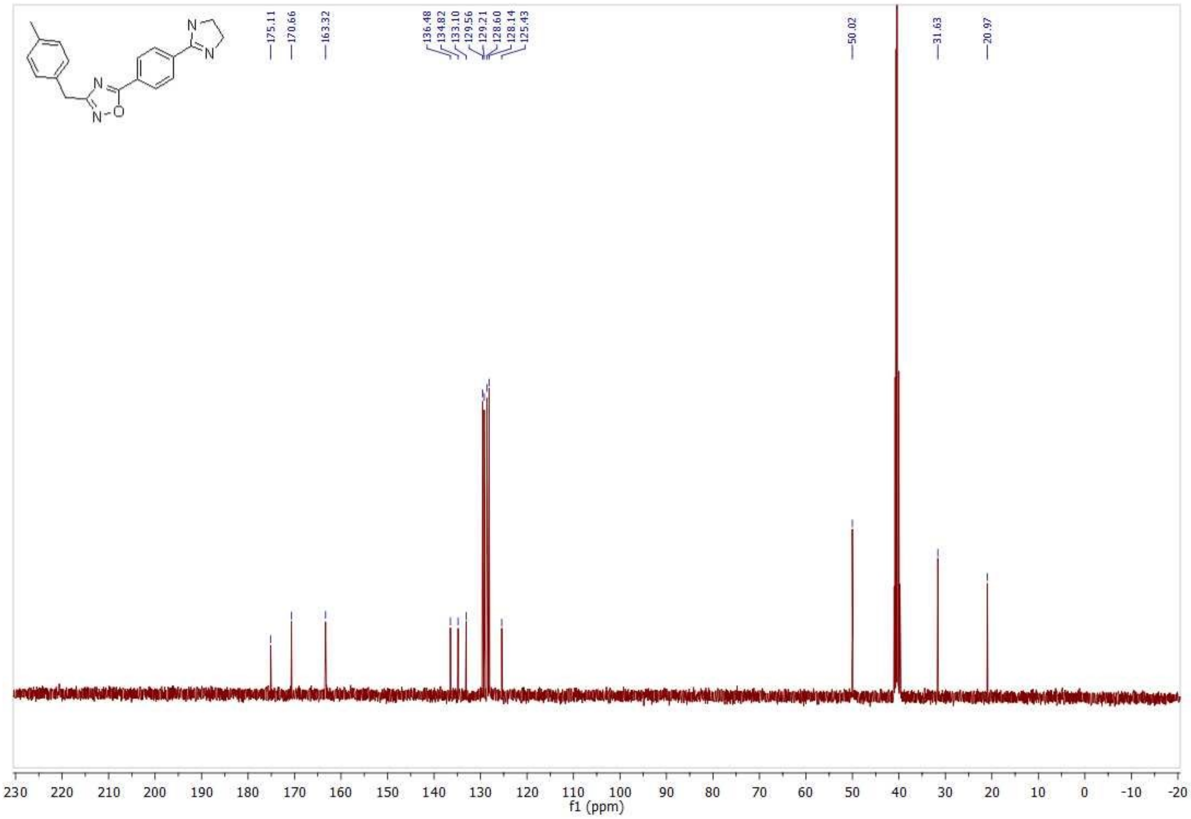

^{1}H and ^{13}C NMR spectra of 5-([1,1'-biphenyl]-4-yl)-3-(4-(4,5-dihydro-1*H*-imidazol-2-yl)phenyl)-1,2,4-oxadiazole (**5n**)

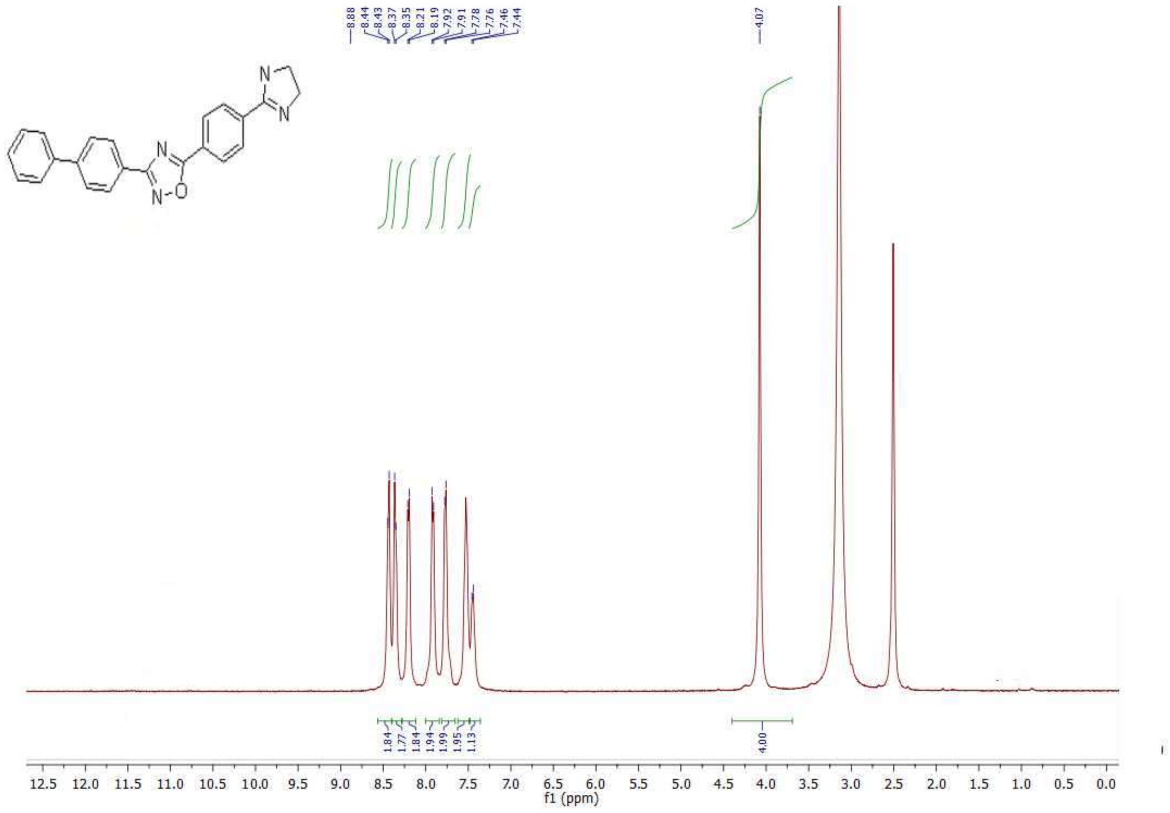

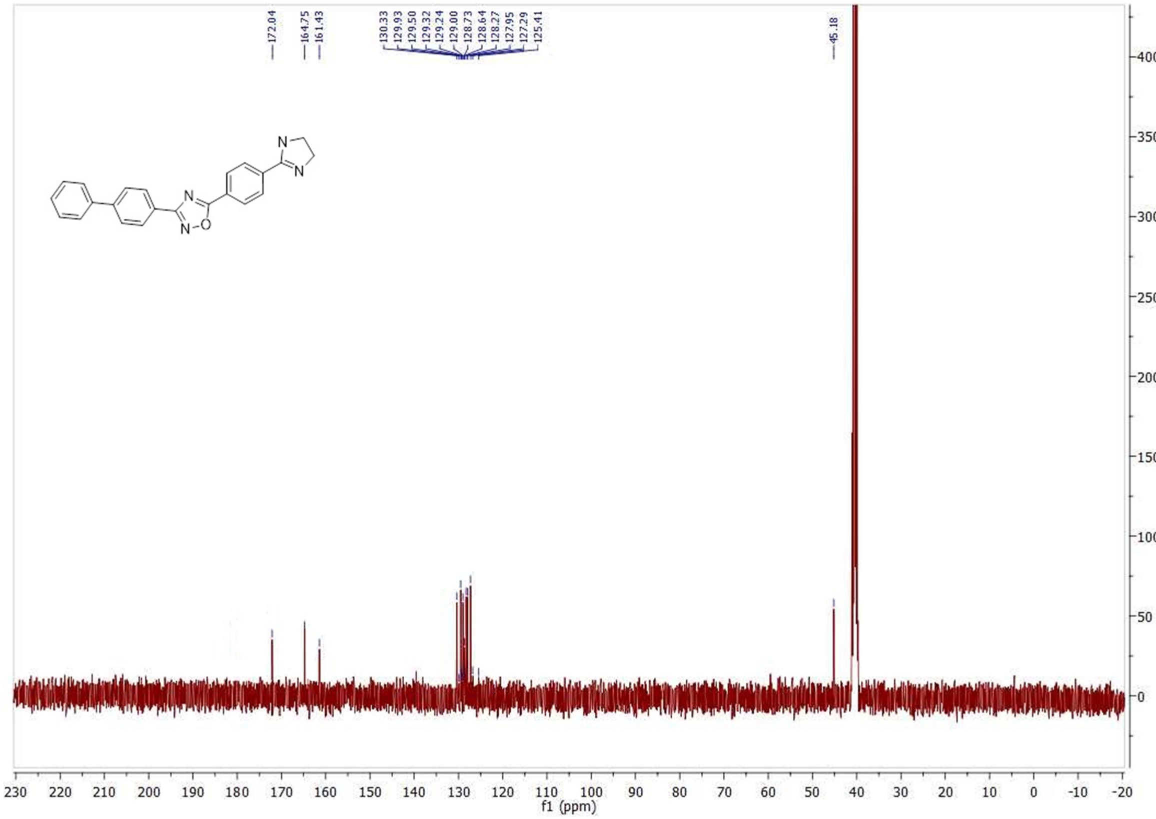

^{1}H and ^{13}C NMR spectra of 5-(4-(4,5-dihydro-1*H*-imidazol-2-yl)phenyl)-3-(4-(((1-methyl-1*H*-imidazol-2-yl)thio)methyl)phenyl)-1,2,4-oxadiazole (**5o**)

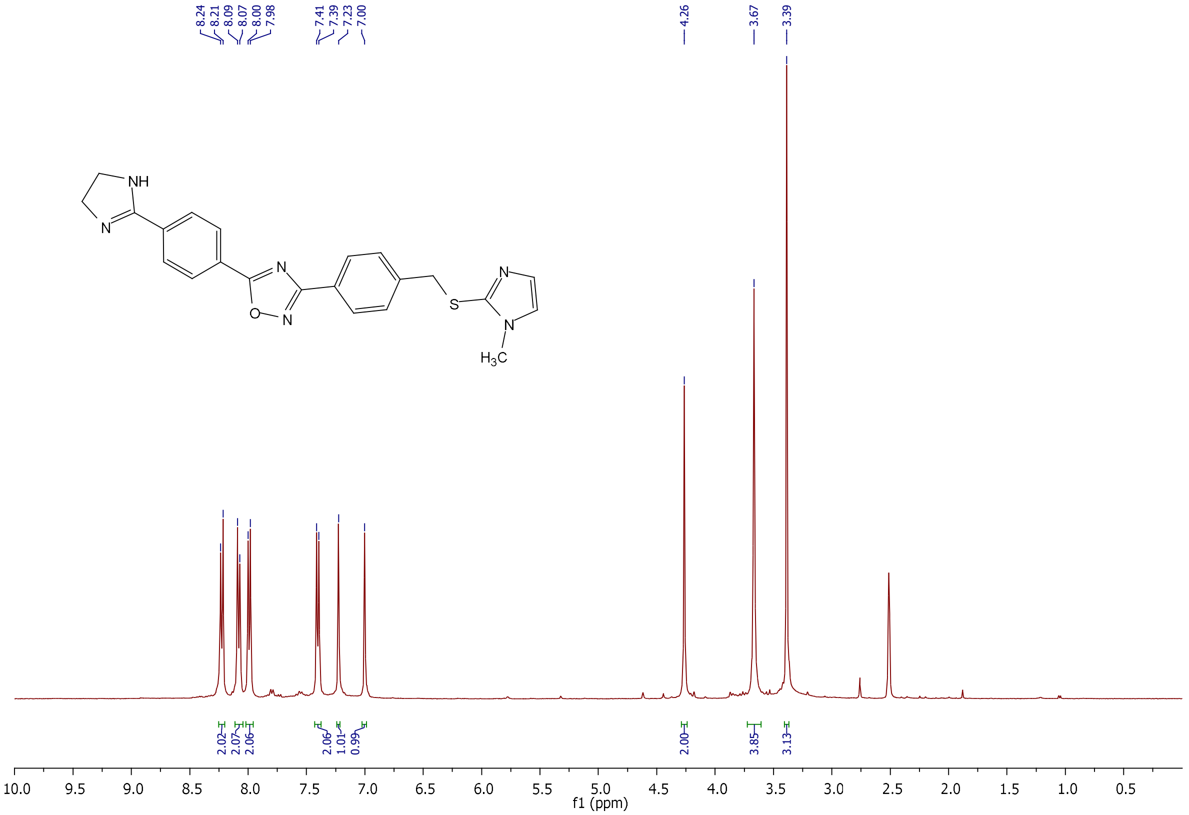

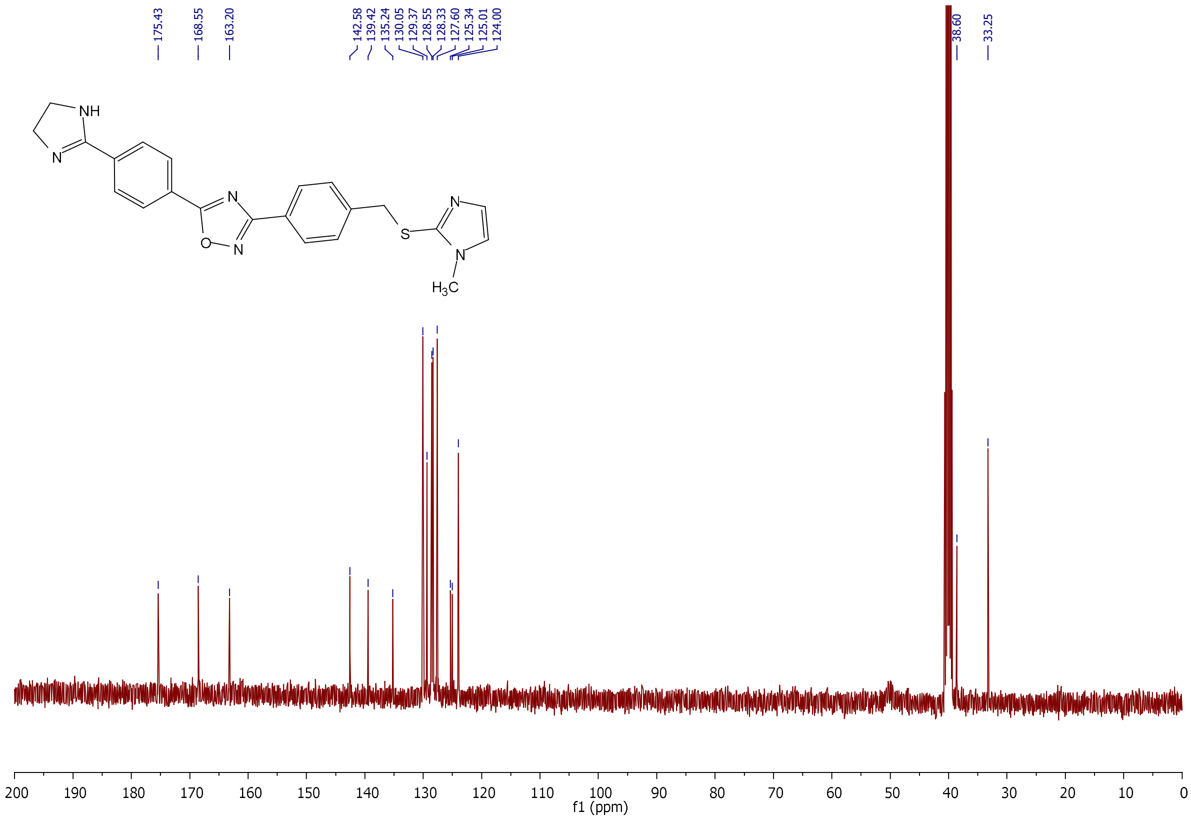

^{1}H and ^{13}C NMR spectra of 5-(3,4-dichlorophenyl)-3-(4-(4,5-dihydro-1*H*-imidazol-2-yl)phenyl)-1,2,4-oxadiazole (**5p**)

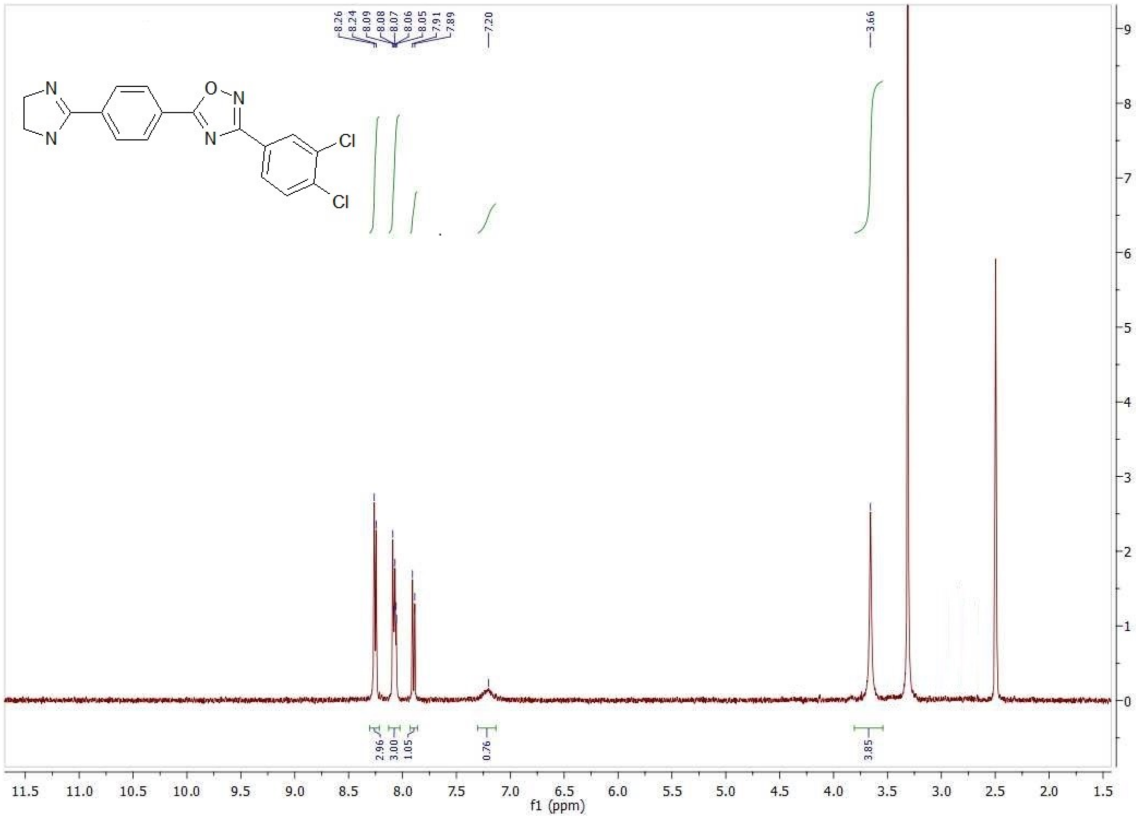

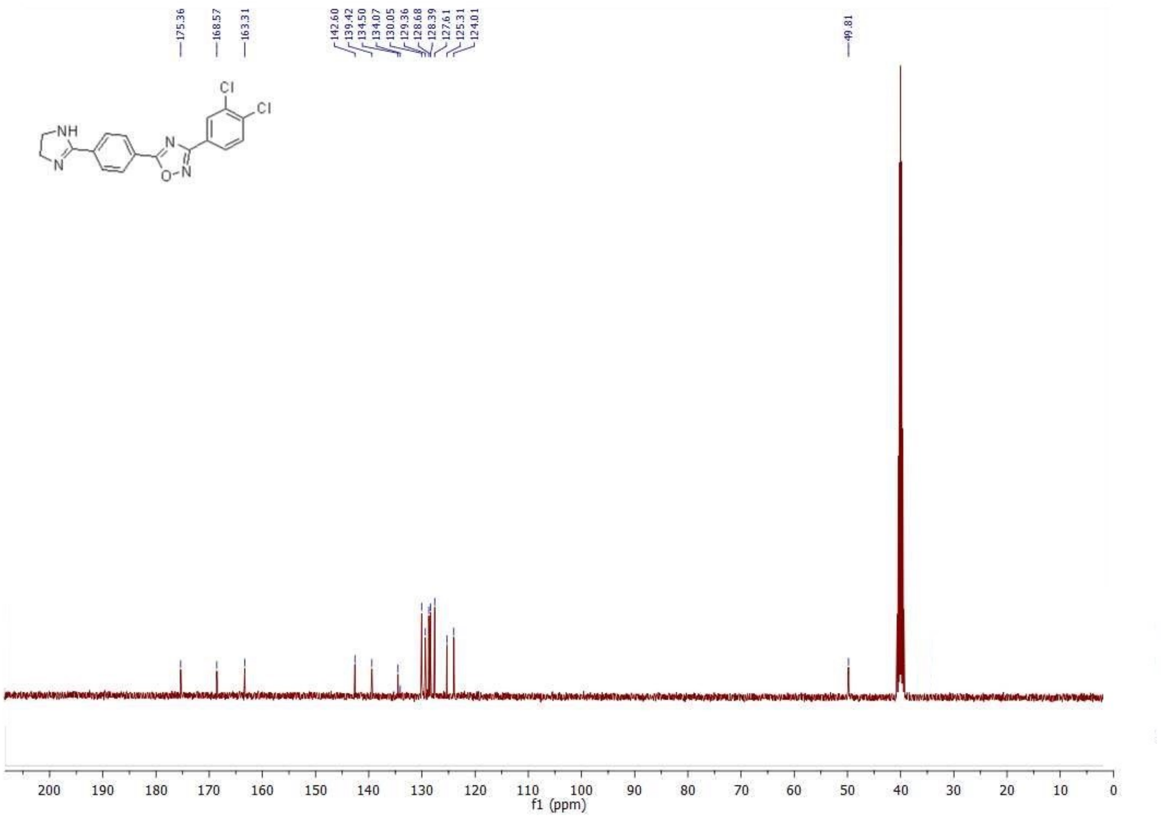

^{1}H and ^{13}C NMR spectra of 5-(4-(5-methyl-4,5-dihydro-1*H*-imidazol-2-yl)phenyl)-3-(5-methylthiophen-2-yl)-1,2,4-oxadiazole (**5q**)

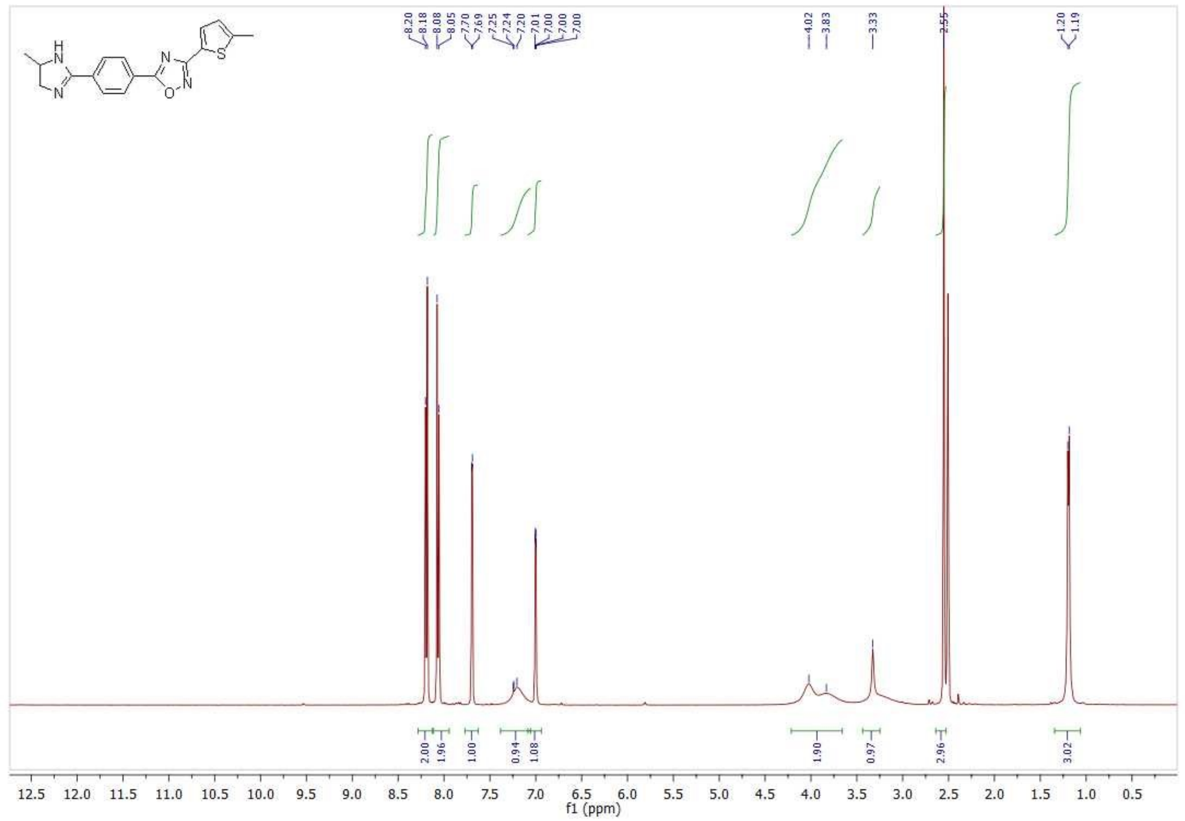

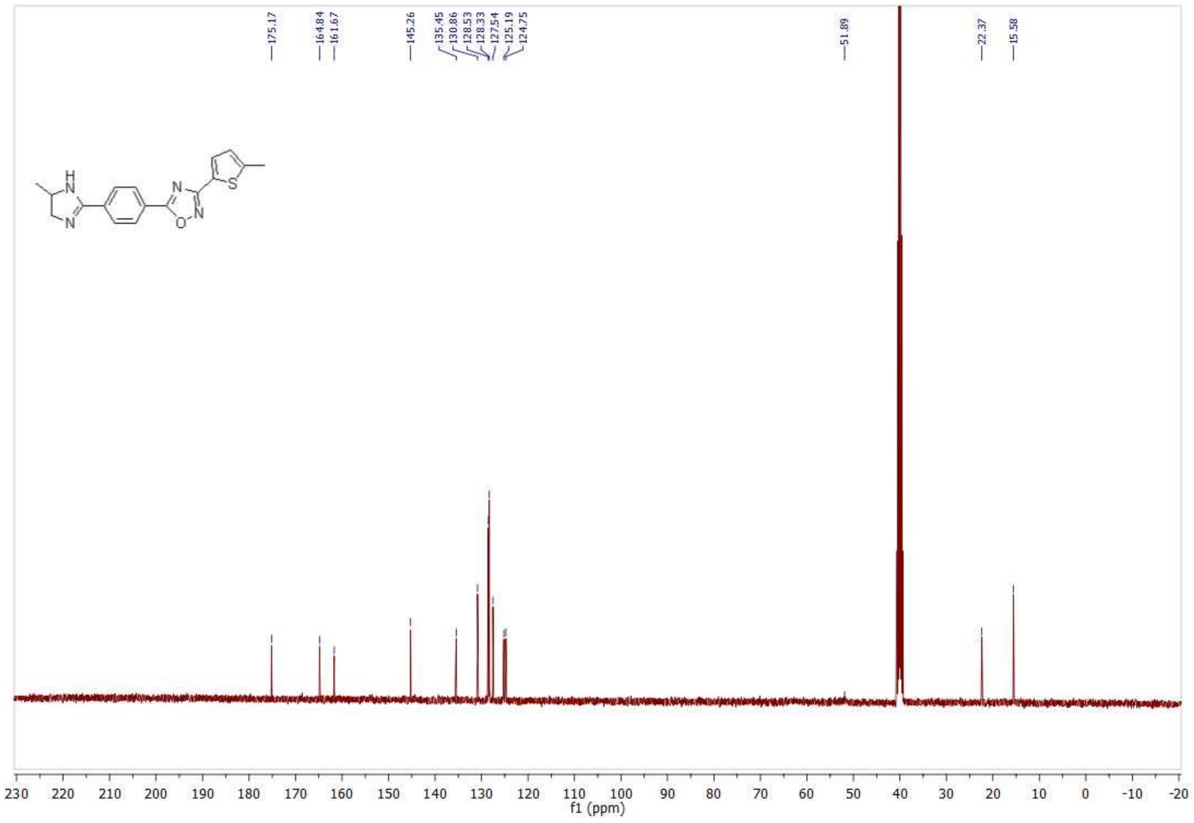

^{1}H and ^{13}C NMR spectra of 5-(4-(5-Methyl-4,5-dihydro-1H-imidazol-2-yl)phenyl)-3-(p-tolyl)-1,2,4-oxadiazole (**5r**)

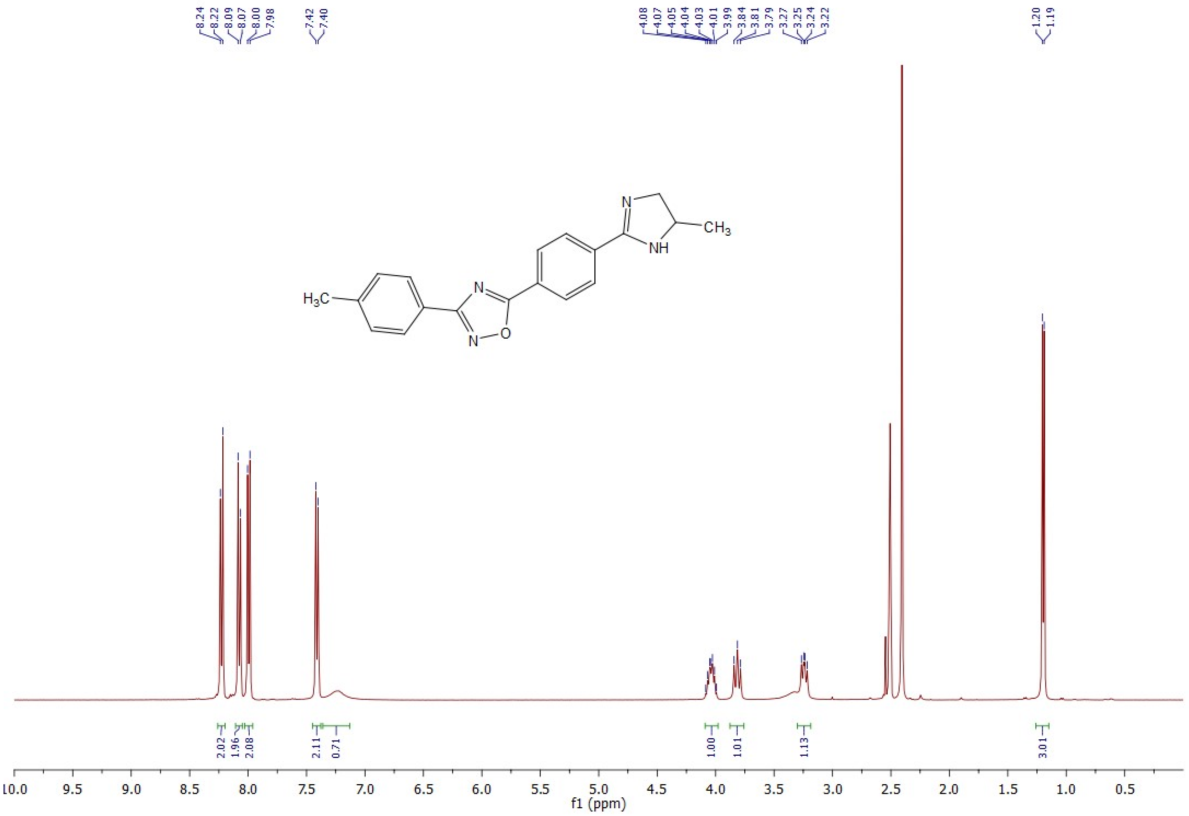

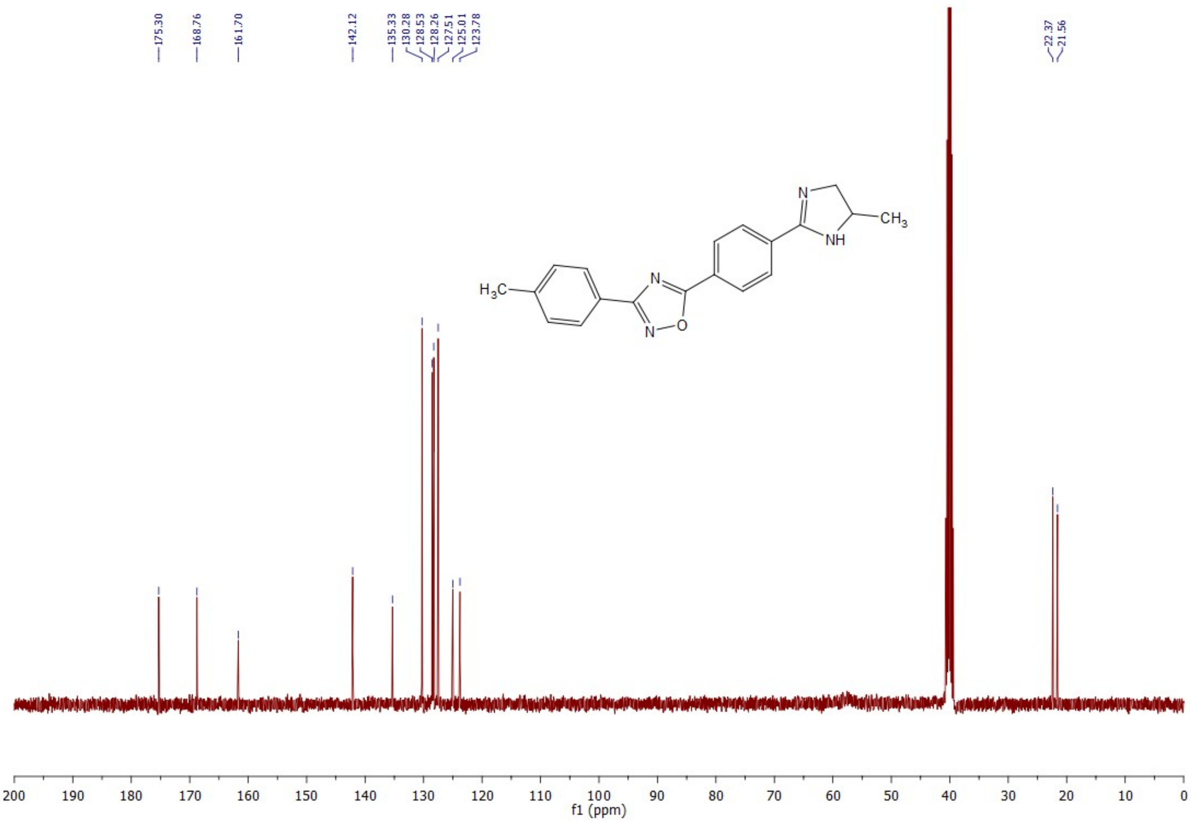

^{1}H and ^{13}C NMR spectra of 3-(3-chlorophenyl)-5-(4-(5-methyl-4,5-dihydro-1H-imidazol-2-yl)phenyl)-1,2,4-oxadiazole (**5s**)

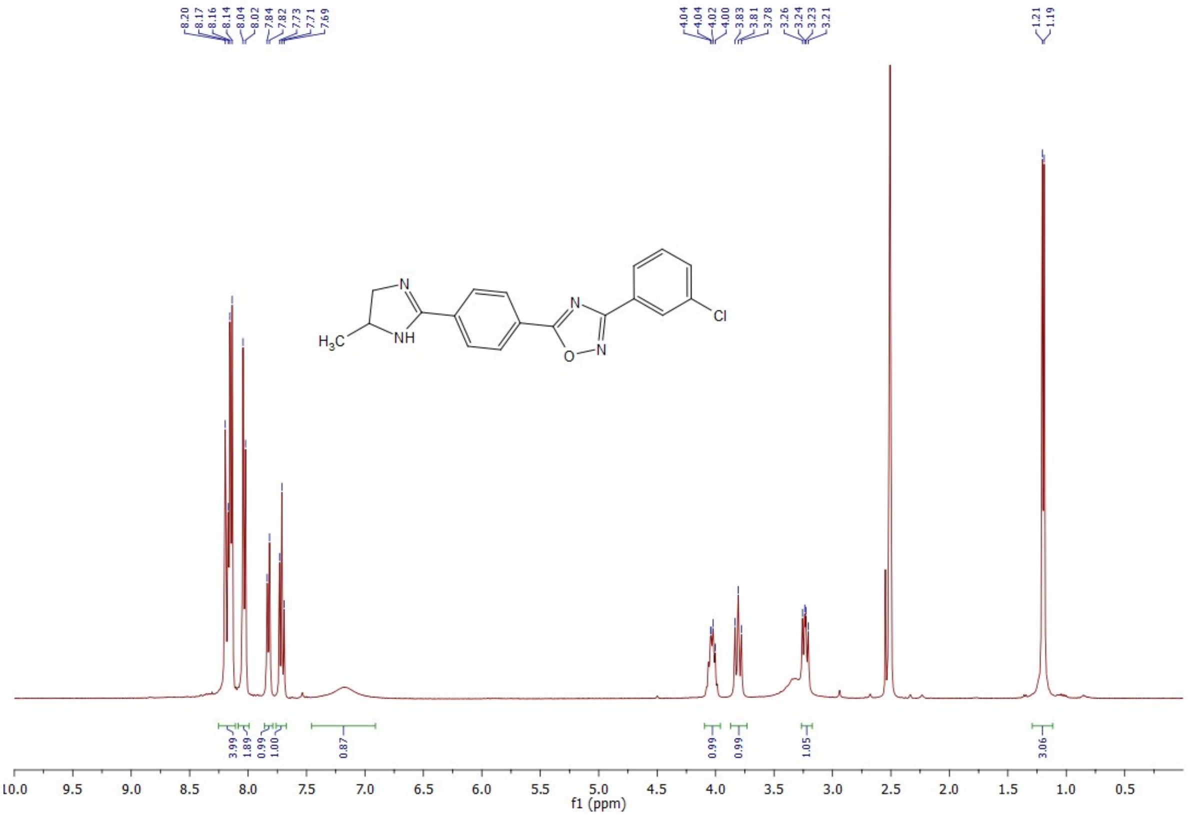

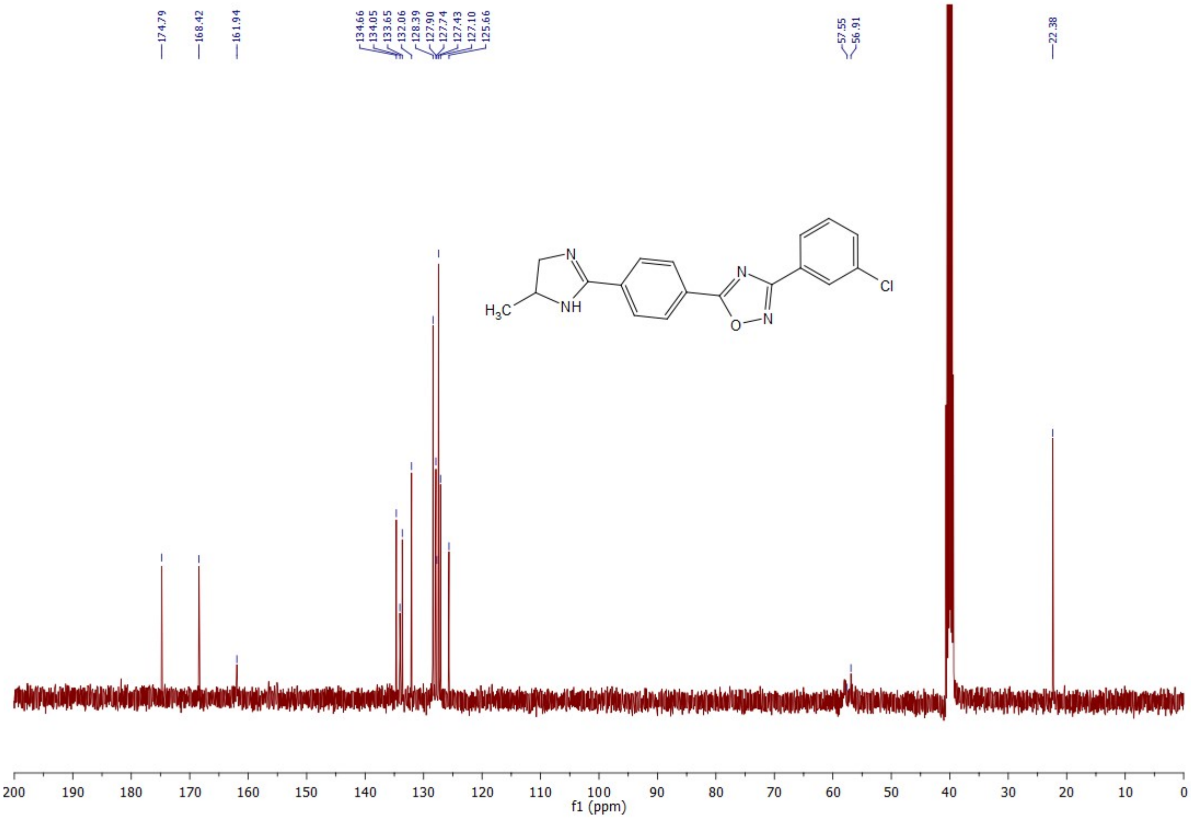

^{1}H and ^{13}C NMR spectra of 5-(4-(4,5-dihydro-1H-imidazol-2-yl)phenyl)-3-(p-tolyl)-1,2,4-oxadiazole (**5t**)

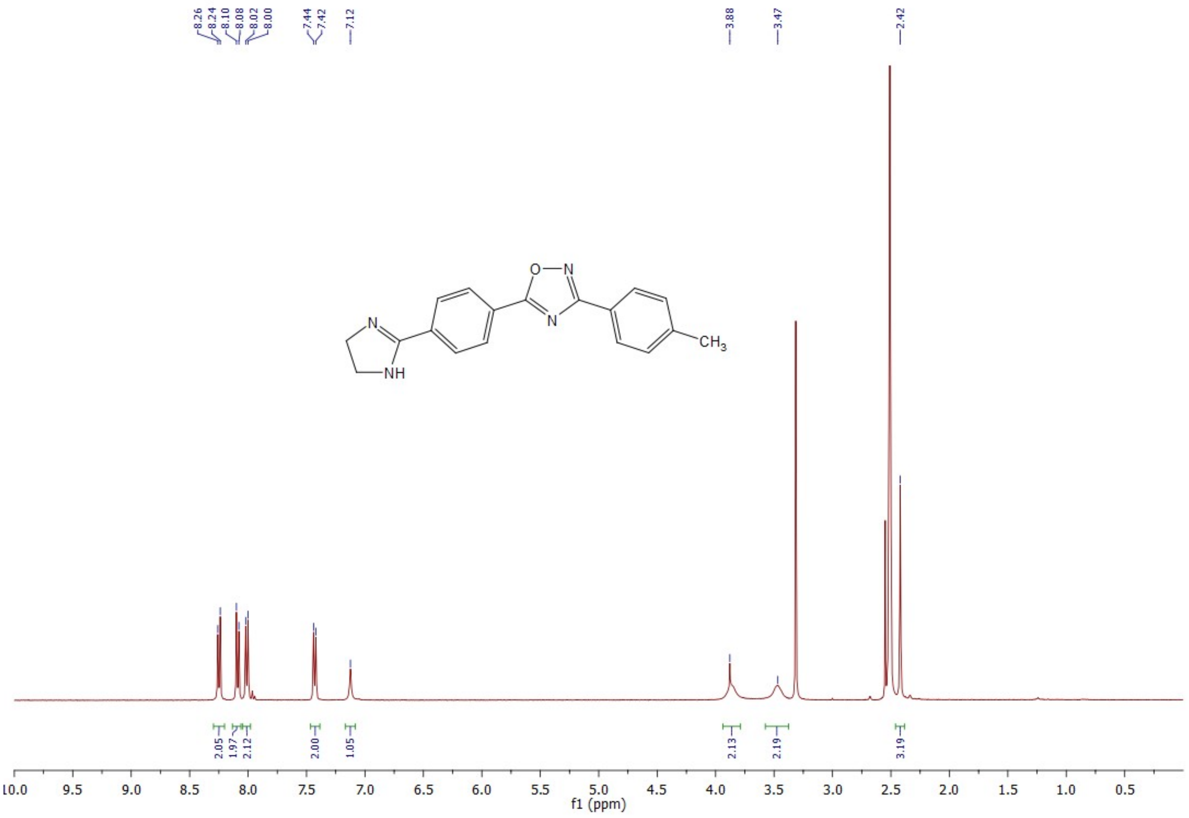

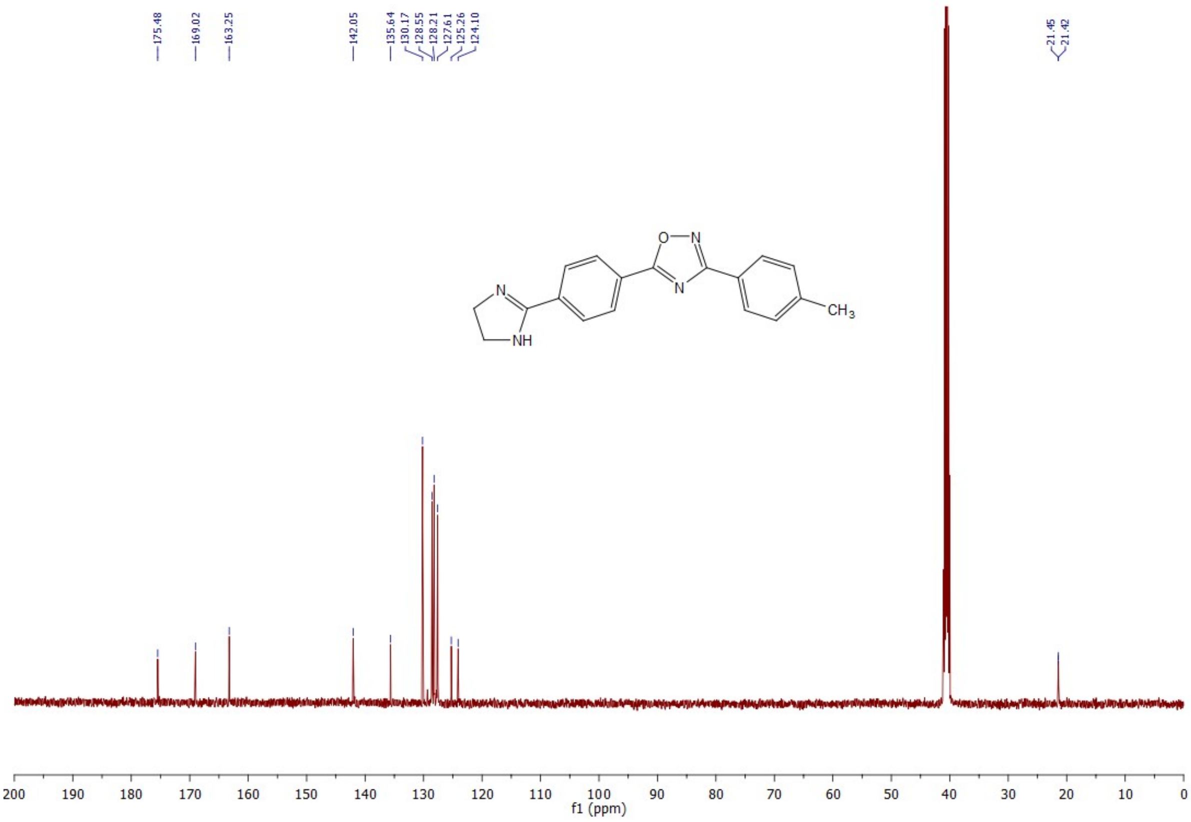

Supplement: Supplementary file 1 [file ijms-20-01699-s001.pdf]
